# The Pancreatic Cancer-Initiating Cell Marker CD44v6 Affects Transcription, Translation, and Signaling: Consequences for Exosome Composition and Delivery

**DOI:** 10.1155/2019/3516973

**Published:** 2019-08-07

**Authors:** Hanxue Sun, Sanyukta Rana, Zhe Wang, Kun Zhao, Martina Schnölzer, Jan Provaznik, Thilo Hackert, Qingjie Lv, Margot Zöller

**Affiliations:** ^1^Tumor Cell Biology, University Hospital of Surgery, Heidelberg, Germany; ^2^Functional Proteome Analysis, German Cancer Research Center, Heidelberg, Germany; ^3^Gene Core Unit, EMBL Heidelberg, Germany; ^4^Section of Pancreas Research, University Hospital of Surgery, Heidelberg, Germany; ^5^Department of Pathology, Shengjing Hospital of China Medical University, Shenyang, China

## Abstract

Pancreatic cancer-initiating cells (PaCIC) express CD44v6 and Tspan8. A knockdown (kd) of these markers hinders the metastatic capacity, which can be rescued, if the cells are exposed to CIC-exosomes (TEX). Additional evidence that CD44v6 regulates Tspan8 expression prompted us to explore the impact of these PaCIC markers on nonmetastatic PaCa and PaCIC-TEX. We performed proteome, miRNA, and mRNA deep sequencing analyses on wild-type, CD44v6kd, and Tspan8kd human PaCIC and TEX. Database comparative analyses were controlled by qRT-PCR, Western blot, flow cytometry, and confocal microscopy. Transcriptome analysis of CD44 versus CD44v6 coimmunoprecipitating proteins in cells and TEX revealed that Tspan8, several signal-transducing molecules including RTK, EMT-related transcription factors, and proteins engaged in mRNA processing selectively associate with CD44v6 and that the membrane-attached CD44 intracytoplasmic tail supports Tspan8 and NOTCH transcription. Deep sequencing uncovered a CD44v6 contribution to miRNA processing. Due to the association of CD44v6 with Tspan8 in internalization prone tetraspanin-enriched membrane domains (TEM) and the engagement of Tspan8 in exosome biogenesis, most CD44v6-dependent changes were transferred into TEX such that the input of CD44v6 to TEX activities becomes largely waved in both a CD44v6kd and a Tspan8kd. Few differences between CD44v6kd- and Tspan8kd-TEX rely on CD44v6 being also recovered in non-TEM derived TEX, highlighting distinct TEX delivery from individual cells that jointly account for TEX-promoted target modulation. This leads us to propose a model in which CD44v6 strongly supports tumor progression by cooperating with signaling molecules, altering transcription of key molecules, and through its association with the mRNA processing machinery. The association of CD44v6 with Tspan8, which plays a crucial role in vesicle biogenesis, promotes metastases by transferring CD44v6 activities into TEM and TEM-independently derived TEX. Further investigations of the lead position of CD44v6 in shifting metastasis-promoting activities into CIC-TEX may offer a means of targeting TEX-CD44v6 in therapeutic applications.

## 1. Introduction

Current models attribute most cancer-related mortality to subpopulations of cancer-initiating cells (CIC), which make up a small proportion of the total mass of most solid tumors [1]. CIC produce exosomes (TEX) containing molecules that can confer tumorigenic properties into cells that would otherwise be benign (non-CIC) [2, 3]. Several CIC-markers, which are recovered in TEX [4], are known to contribute to tumor cell dissemination and metastatic settlement. However, whether these markers are capable of transferring tumorigenicity through TEX is unknown [5]. Neither the mechanisms by which CIC markers are recruited into TEX nor their functions in the formation of TEX have been comprehensively explored.

We approached these questions for two pancreatic CIC (PaCIC) markers, CD44v6 and Tspan8 [6]. PaCa were chosen because of the early metastatic spread [7]. The choice of the markers was based on strong evidence for metastasis-promoting activities of CD44v6 [8], the enrichment of tetraspanins in exosomes [9], and our recent observation on an engagement of CD44v6 in Tspan8 transcription [10].

CD44v6 might contribute to tumor progression in many ways. CD44v6 is associated with receptor tyrosine kinases (RTK) [11] and is engaged in Wnt signaling via associated LRP6 (LDL receptor related protein 6), which promotes *β*-catenin pathway activation [12]. It is involved via NOTCH and Nanog in gene activation responsible for the epithelial-mesenchymal transition (EMT) [13, 14]. CD44v6 also plays a role in apoptosis resistance, partly through its impact on drug efflux [15, 16]. It contributes to matrix remodeling and degradation via the activation of protease transcription and protease proform cleavage [17]. Finally, CD44/CD44v6 can regulate miRNA processing [18, 19].

The metastasis-promoting tetraspanin Tspan8 associates with integrins and proteases, which promotes a migratory phenotype and opens a path for tumor cell egress [20, 21]. Tetraspanins are located in glycolipid-enriched membrane domains (TEM), prone for internalization [22]. TEM complexes remain stable during invagination and exosome (Exo) biogenesis [23]. The association between Tspan8 and CD44v6 within TEM implies that they should be recovered together in TEX. Since CD44v6 has been shown to affect Tspan8 expression [10], this would functionally link two PaCIC markers in a platform that could contribute to tumor progression via TEX.

Exosomes are a subpopulation of small vesicles with a lipid bilayer membrane and incorporated proteins. The protein content, partly determined by proteins located in internalization-prone membrane domains including TEM [22], is reflected by highly enriched tetraspanin recovery in Exo [24]. The small cytoplasm is loaded with proteins and coding and noncoding RNA and DNA [25] during vesicle invagination into multivesicular bodies (MVB) in a selective manner. Protein sorting is facilitated by monoubiquitination, acylation, or myristoylation, higher-order oligomerization, and ceramide-forming sphingolipids [26]. Annexin-II plays a role in RNA sorting into Exo by binding specific RNA [27]. miRNA recruitment is guided by a sequence within the 3′-UTR and by the coupling of the RISC (RNA-induced silencing complex) to components of the sorting complex. A specific EXOmotif (GGAG) binds to the heterogeneous ribonucleoprotein A2B1, which binds to an RNA transport signal (A2RE) [28].

Exo are the body's most powerful resource of cell-cell contact-independent message transfer. By the efficacy of Exo in target modulation/reprogramming, TEX might be a point of attack for novel forms of therapy [7]. This would require a clearer view of TEX biogenesis and function. We used the CD44v6 knockdown (kd) and Tspan8kd PaCa lines to determine the manner in which CD44v6 contributes to Tspan8 transcription. Proteome and deep sequencing (DS) analyses revealed that CD44v6 plays a role in EMT and the processing of miRNA. CD44v6-promoted activities become prominent in TEX due to the CD44v6-Tspan8 association and the engagement of Tspan8 in Exo biogenesis.

## 2. Methods

*Tumor Lines*. The human PaCa lines A818.4, Capan-1, and AsPC1 were kindly provided by the Department of Pathology, University of Kiel, Germany. Frozen early passages were used for transfection. CD44v6- or Tspan8-shRNA (Qiagen, Hilden, Germany) transfected lines are described [10]. A818.4 cells were transiently transfected with the CD44 intracellular domain (ICD). Primers are listed in Table S1. Cells were cultured in RPMI1640/10%FCS/pyruvate/L-glutamine/antibiotics, adding 0.5mg/ml G418 for the kd lines in a humidified atmosphere at 37°C, 5%CO_2_ in air. Cells were regularly checked for mycoplasma contamination. 

*CIC Enrichment and TEX Collection.* Capan1-CIC were enriched by spheroid growth and A818.4 CIC by holoclone formation [10]. After 3 rounds of cloning, spheres/holoclones were seeded at subconfluent density in 250ml flasks and were cultured for 48h in 15ml FCS-free RPMI1640 for harvesting TEX. After 24h recovery (RPMI1640 with 10% Exo-depleted FCS), cells were cultured for an additional 48h in 15ml FCS-free RPMI1640 for TEX collection. Thereafter CIC-enriched cells were discarded. Cell viability of ~98% at the beginning of the collection procedure remained unaltered. 

*Antibodies.* Antibodies are listed in Table S2. 

*TEX Preparation*. Tumor cell supernatants were cleared (2x10min, 500g, 1x20min, 2000g, and 1x30min, 10000g, 4°C), filtered (0.22*μ*m), and centrifuged (Beckman Coulter ultracentrifuge, Type 45 Ti rotor, 50ml, 120min, 100000g, 4°C). The pellet was resuspended and washed (PBS, 120min, 100000g, 4°C). The same procedure was used to deplete FCS from exosomes, collecting the supernatants. After washing the pellet, it was resuspended in 0.8ml HEPES and mixed with 0.8ml 80% sucrose and layered at the bottom of 4ml ultracentrifugation tubes. The 40% sucrose was overlaid with 1.6ml 30% and 0.8ml 5% sucrose gradient and centrifuged (Beckman Coulter ultracentrifuge, SW41Ti rotor, 4ml tubes, 16h, 100000g, 4°C), collecting 12 fractions of 320*μ*l, with TEX being enriched in fractions 1-4 (light density fractions, d: 1.15-1.56g/ml). Protein concentrations were determined by Bradford. Where indicated, TEX were labeled with SP-DioC_18_(3) (3,3′-dioctadecyl-5,5′-di(4-sulfophenyl)oxacarbocyanine). After quenching (15ml Exo-depleted FCS) and washing (Beckman Coulter ultracentrifuge, SW41Ti rotor, 4ml tubes, 2x120min, 100000g, 4°C), the pelleted TEX were collected in 200*μ*l PBS. Alternatively, for further depletion of free dye, TEX were suspended in 30ml PBS layered over 10ml 40% sucrose and centrifuged (Beckman Coulter ultracentrifuge, Type 45 Ti rotor, 50ml tubes, 120min, 100000g, 4°C), collecting the TEX-pellet at the bottom in 200*μ*l PBS. TEX preparations either were stored at -80°C or were used immediately for mass spectrometry and mRNA/miRNA sequencing [29]. 

*Protein Elution, Tryptic Digestion, Mass Spectrometry, and Database Searches.* Cell and TEX lysates and dissolved immunoprecipitates were separated by 1D SDS gel electrophoresis on a NuPAGE 4-12% Bis-Tris gradient gel (8 cm x 8 cm, Invitrogen, Carlsbad, USA) using a MOPS-buffer system. After staining with colloidal Coomassie, entire lanes including the dye front were cut into ten slices of 0.8 cm each. Proteins in the individual gel slices were reduced with DTT, alkylated with iodoacetamide, and in-gel digested with trypsin (Promega, Mannheim, Germany) overnight. Tryptic peptides were extracted from the gel pieces, evaporated to dryness in a speed-vac concentrator, and dissolved in 5*μ*l 0.1% TFA/2.5% hexafluoro-2-propanol prior to analysis by Nano-LC-ESI-MS/MS [30].

Peptide mixtures were separated using a nanoACQUITY UPLC system. Peptides were trapped on a C18 precolumn (180*μ*m × 20mm) with a particle size of 5*μ*m (Waters GmbH, Eschborn, Germany). Liquid chromatography separation was performed on a BEH130 C18 main-column (100*μ*m × 100mm) with a particle size of 1.7*μ*m (Waters GmbH, Eschborn, Germany). Peptide mixtures were loaded on the trap column at a flow rate of 5*μ*l/min and were eluted with a gradient at a flow rate of 400nl/min. Chromatography was carried out using a 1h gradient of solvent A (98.9% water, 1% acetonitrile, 0.1 % formic acid) and solvent B (99.9% acetonitrile and 0.1% *μ*l formic acid) in the following sequence: from 0 to 4% B in 1 min, from 4 to 40% B in 40 min, from 40 to 60% B in 5 min, from 60 to 85% B in 0.1 min, 6 min at 85% B, from 85 to 0% B in 0.1 min, and 9 min at 0% B. The nanoUPLC system was coupled online to an LTQ-Orbitrap XL mass spectrometer (Thermo Scientific, Bremen, Germany). The mass spectrometer was operated in data-dependent mode to automatically measure MS1 and MS2. Data were acquired by scan cycles of one FTMS scan with a resolution of 60000 at* m/z* 400 and a range from 300 to 2000* m/z* in parallel with six MS/MS scans in the linear ion trap of the most abundant precursor ions [30].

The mgf-files generated by Xcalibur software (Thermo Scientific, Bremen, Germany) were used for database searches with the MASCOT search engine (version 2.4.1, Matrix Science, London, UK) against the SwissProt database (SwissProt 2017_01 (553474 sequences; 198069095 residues) with taxonomy human (20246 sequences). Each slice was analyzed separately by MS and MS/MS. Data of each slice were merged into one single file prior to protein database search. Peptide mass tolerance for database searches was set to 5ppm and fragment mass tolerance to 0.4 Da. Carbamidomethylation of C was set as fixed modification. Variable modifications included oxidation of M and deamidation of NQ. One missed cleavage site in case of incomplete trypsin hydrolysis was allowed. Furthermore, proteins were considered as identified if more than one unique peptide had an individual ion score exceeding the MASCOT identity threshold [30]. Samples are deposited at functional proteome analysis, German Cancer Research Center, Heidelberg (files: ZW2612, ZW2484, and SH2726). 

*mRNA and miRNA.* TEX were pretreated with RNAse to eliminate unspecifically attached RNA. Both cell and TEX mRNA and miRNA were extracted using mRNA and miRNA extraction kits according to the supplier's suggestion (Qiagen, Hildesheim, Germany). Transient transfection with CD44ICD followed standard protocols (Qiagen, Hildesheim, Germany). Where indicated, cells were pretreated by anti-CD44v6 crosslinking (10*μ*g/ml, 1h, 4°C) or PMA (10^−8^M, overnight, 37°C) or a *γ*-secretase inhibitor (5*μ*M, 24h, 37°C).

*Microarray miRNA Analysis.* DS mRNA and miRNA analyses were performed at the Core Facility of EMBL, Heidelberg (ENA database, accession no.: PRJEB25446). The alignment software used was STAR aligner version 2.5.2a, reference hg19. Mean values of normalized data were compared. Differential recovery was defined by ≥1.5- or ≥2-fold changes in mean signal strength of normalized data. 

*Proteome and miRNA Analysis.* The following databases were used: PANTHER (http://pantherdb.org), KEGG (http://www.kegg.jp), Reactome (https://reactome.org), and STRING (http://string-db.org). IPA was used for correlating miRNA with protein expression according to mRNA predictions (http://www.microrna.org, http://diana.imis.athena-innovation.gr/DianaTools/index.php, and http://www.targetscan.org). For heatmap analysis, targeted pathways cluster/heatmap was used, which indicates all significantly targeted pathways with p values under the selected threshold and 1 otherwise (http://diana.imis.athena-innovation.gr/DianaTools/index.php, mirPath v.3) [31]. 

*Real-Time PCR (qRT-PCR).* Real-time polymerase chain reaction (PCR) was performed using a standard TaqMan PCR kit protocol on an Applied Biosystems 7900HT Sequence Detection System (Applied Biosystems). The 10*μ*l PCR included 0.67*μ*l of reverse transcriptase product, 1x TaqMan Universal PCR Master Mix (Applied Biosystems), 0.2*μ*M TaqMan probe, 1.5*μ*M forward primer, and 0.7*μ*M reverse primer. The reactions were incubated in a 384-well plate at 95°C for 10min, followed by 40 cycles of 95°C for 15sec and 60°C for 1min. All reactions were run in triplicate [32]. Small nuclear snRNA U6 was used as internal control for miRNA. Primers are listed in Table S1B. The threshold cycle (*C*_T_) is defined as the fractional cycle number at which the fluorescence passes the fixed threshold. TaqMan *C*_T_ values were converted into absolute copy numbers using a standard curve from synthetic lin-4 miRNA. Statistical analysis was done by the Δ*C*_T_ method (^Δ^*C*_T_ = *C*_T_ test gene - *C*_T_ endogenous control; ^ΔΔ^*C*_T_ = ^Δ^*C*_T_ sample - Δ*C*_T_ calibrator). For RQ (relative quantification/fold change) wt cells or TEX were used as reference. 

*Flow Cytometry.* TEX (10–15 *μ*g) were incubated with 1*μ*l of aldehyde-sulfate latex beads (4*μ*m) (Invitrogen) in PBS/1%BSA (90min, 20°C, shaking). After centrifugation, free binding sites on the beads were blocked by incubation with 100mM glycine in PBS (1h). After washing two times with PBS/1%BSA, TEX-coated beads (corresponding to 1*μ*l beads/well) were distributed in 96-well plates. Coated LB and cells (2x10^5^/well) were incubated on ice with 30*μ*l primary antibody in PBS/1%BSA for 30min. The concentration of the primary antibody was evaluated in advance and varied from 0.1 to 50*μ*g/ml. LB/cells were washed 3 times with 200*μ*l cold PBS/1%BSA and were incubated for 30min on ice with dye-labeled secondary antibody at predetermined concentrations. LB/cells were washed 3 times and resuspended in 200*μ*l PBL/1%BSA. For intracellular staining, cells/TEX were fixed and permeabilized [29]. Samples were analyzed in a FACSCalibur using the CellQuest program. 

*Immunoprecipitation (IP) and Western Blot (WB).* Lysates (IP: cell lysate 500*μ*g, TEX lysate: 100*μ*g; WB: cell lysate 30*μ*g, TEX lysate: 10*μ*g) (30min, 4°C, HEPES buffer, 1% Lubrol or 1% TritonX-100, 1mM PMSF, 1mM NaVO_4_, 10mM NaF, protease inhibitor mix) were centrifuged (13000g, 10min, 4°C), mixed with antibody (1h, 4°C), and incubated with ProteinG-Sepharose (1h). Washed complexes/lysates, dissolved in Laemmli buffer, were resolved on 10%-12% SDS-PAGE. After protein transfer, blocking, and blotting with antibodies, blots were developed with enhanced chemiluminescence (ECL) WB-detection-reagent. 

*Sucrose Density Gradient of Cell Lysates.* Sucrose density gradient of cell lysates followed the protocol described for TEX. 

*Statistics.* IBM SPSS software (IBM, New York, NY, USA) was used for statistical evaluation. qRT-PCR samples were run in triplicates and repeated 2 to 3 times. Flow cytometry analysis, WB, IP, and sucrose density gradients were repeated at least three times. P values are derived from two-tailed Student's* t*-test and analysis of variance. If not indicated otherwise,* p* values <0.05 were considered significant. 

*Data Accessibility.* Proteome analysis (files nos. ZW2612, ZW2484, and SH2726) is available at Functional Proteome Analysis, DKFZ, Heidelberg, Im Neuenheimer Feld 280, D69120 Heidelberg, Germany (Dr. Martina Schnölzer, e-mail: m.schnoelzer@dkfz-heidelberg.de). MicroRNA microarray data are deposited at GEO (http://www.ncbi.nlm.nih.gov/geo/query/acc.cgi?acc=GSE119031, GSE119032, and GSE11903) and DS miRNA and RNA analysis at ENA database (accession no.: PRJEB25446).

## 3. Results

CD44v6 and Tspan8 are PaCIC-biomarkers [6], with message delivery by CIC-TEX being expected to convert nonmetastasizing tumor cells into CIC [33]. CD44v6 cooperates in cells and TEX with metastasis-promoting signaling molecules and transcription factors [34], contributes to Tspan8 expression [10], and is suggested to be involved in miRNA regulation. Tspan8 is engaged in TEX biogenesis and binding [29]. Unraveling the link between CD44v6 and Tspan8 and the contribution of these PaCIC-biomarkers to TEX biogenesis and assembly might provide a means to attack CIC-TEX. We here approached an answer using wt and CD44v6kd or Tspan8kd human PaCa lines and TEX for proteome, miRNA, and mRNA analyses. In advance, we controlled for the impact of CD44v6 on Tspan8 transcription and the impact of CD44v6 and Tspan8 on TEX delivery and uptake.

### 3.1. Tspan8 Transcription Is Regulated by CD44v6

**Figure 1:**
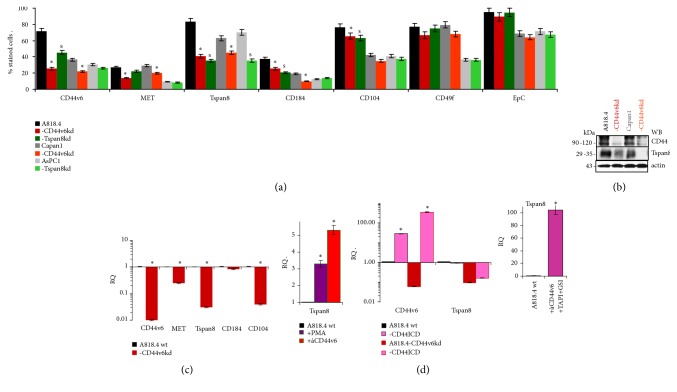
*A CD44v6kd affects Tspan8 transcription*. (a) Flow cytometry of PaCIC markers in CD44v6kd and Tspan8kd cells; mean % stained cells ± SD (3 experiments); significant differences between wt and CD44v6kd cells: *∗*, significant differences between wt and Tspan8kd cells: s. (b) Representative WB (one of 3 experiments) of CD44v6 and Tspan8 in wt and CD44v6kd lines. (c) RQ±SD (triplicates, 2 repetitions) of CD44v6, MET, Tspan8, CD184, and CD104 RNA as revealed by a qRT-PCR; significant differences to wt cells: *∗*. (d) RQ of CD44v6 and Tspan8 after PMA stimulation, CD44v6 crosslinking, or CD44ICD transfection or in the presence of a *γ*-secretase inhibitor (GSI); RQ±SD of triplicates (2 repetitions); significant differences to untreated A818.4 wt cells: *∗*. A CD44v6kd affects additional CIC marker expression, most prominently Tspan8 that transcription is prohibited and can be partly rescued by CD44v6 activation/crosslinking, which requires CD44ICD-initiated signal transduction.

CIC-marker expression was evaluated by flow cytometry in A818.4, Capan1, and AsPC1 cells. A CD44v6kd is accompanied by reduced CD44v6 and Tspan8, less pronounced MET, and slightly mitigated CD184 and CD104 expression. A Tspan8kd affects Tspan8 and weakly CD44v6, CD184, and CD104 expression. WB and qRT-PCR confirmed the impact of CD44v6 on Tspan8, MET, and CD104 expression (Figures 1(a)–1(c)).

CD44ICD is a potential cotranscription factor that beside others supports CD44 transcription [14]. Being particularly interested in the suggested link between CD44v6 and Tspan8 in TEX biogenesis and delivery, we first searched whether the CD44ICD accounts for Tspan8 transcription, the Tspan8 promoter availing on a binding site. A818.4 stimulation by PMA or CD44v6 crosslinking promoted Tspan8 transcription. However, transfection of A818.4-CD44v6kd cells with the CD44ICD rescued CD44 but not Tspan8 expression. Instead, Tspan8 expression became increased, when the liberation of the CD44ICD was prevented by a *γ*-secretase inhibitor (Figure 1(d)).

These findings confirm the CD44v6 contribution to Tspan8 transcription and indicate a requirement for the membrane-attached CD44ICD but exclude CD44ICD activity as a cotranscription factor. The signaling pathway from the CD44ICD to the Tspan8 promoter remains to be explored.

### 3.2. The Impact of CD44v6 on Tspan8 in TEX Delivery and Uptake

Tspan8 is engaged in TEX-biogenesis [29]. Thus, the impact of CD44v6 on Tspan8 transcription might have bearing on TEX-delivery.

**Figure 2:**
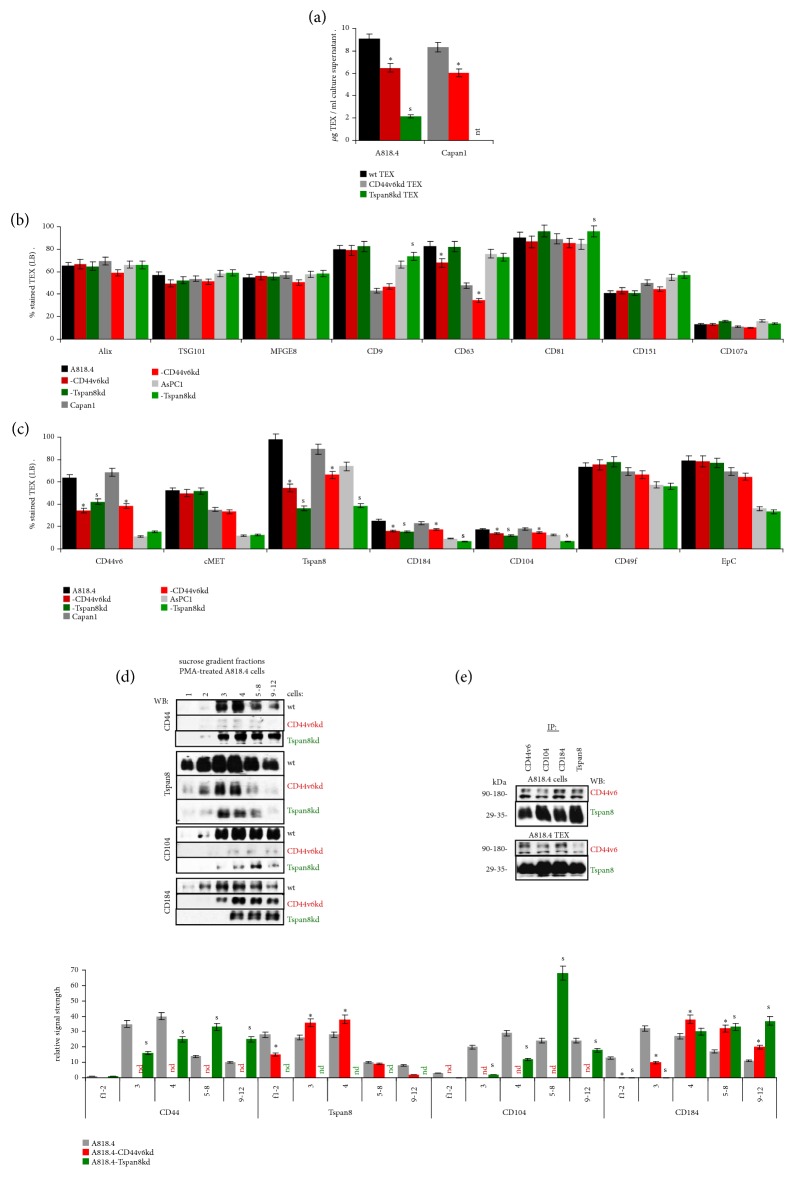
*CD44v6 and Tspan8 affect recovery of CIC markers in TEX*. (a) TEX protein concentrations were determined by Bradford in culture supernatants of wt, CD44v6kd, and Tspan8kd lines; flow cytometry of (b) constitutive Exo marker and (c) CIC marker expression in wt, CD44v6kd, and Tspan8kd TEX; (a-c) mean ± SD of 3 experiments are shown; significant differences between wt and CD44v6kd cells/TEX: *∗*, significant differences between wt and Tspan8kd cells/TEX: s; (d) WB of CIC markers in sucrose gradient fractions of PMA-treated wt, CD44v6kd, and Tspan8kd cells. A representative example and the mean ± SD (3 experiments) of relative signal strength evaluated by ImageJ quantification are shown; significant differences between wt and CD44v6kd cells: *∗*, significant differences between wt and Tspan8kd cells: s; (e) WB of CD44v6 and Tspan8 in immunoprecipitates with the indicated antibodies in A818.4 wt cell and TEX lysates. A representative example of 3 independently performed experiments is shown (nd: not detected). A CD44v6kd and more strongly a Tspan8kd affect TEX delivery. Expression of constitutive Exo markers is not affected. However, expression of CD44v6- and Tspan8-associated molecules is impaired in CD44v6kd- and Tspan8kd-TEX.

TEX-delivery is significantly impaired in CD44v6kd and Tspan8kd cells (Figure 2(a)). However, expression of constitutive Exo-markers (Alix <PDCD6IP, programmed cell death 6 interacting protein>, TSG101 <Tumor susceptibility gene 101 protein>, Lamp1 <lysosomal associated membrane protein 1>, MFGE8 <Lactadherin>) and of tetraspanins (CD9, CD81) was not and that of CD63 was only slightly affected in a CD44v6kd. A tendency towards compensatory overexpression of CD9 and CD81 was noted in Tspan8kd TEX (Figure 2(b)). Reduced Tspan8, CD104, and CD184 expression in CD44v6kd cells was maintained in TEX. Distinct to cells, a Tspan8kd also affected CD44v6 and more strongly CD104 and CD184 expression in TEX (Figure 2(c)). This could be a sequel of CD184 and CD104 associating with Tspan8 during TEM internalization [6, 29].

The latter suggestion was controlled by the recovery of Tspan8, CD44, CD104, and CD184 in light sucrose gradient fractions of PMA stimulated A818.4-wt, -CD44v6kd, and –Tspan8kd cells. Irrespective of a CD44v6kd, Tspan8 was recovered in light density fractions 1-4, yet with a slight shift from fractions 1 and 2 to fractions 3 and 4. In contrast, CD44, CD104, and CD184 were shifted to heavier fractions in Tspan8kd cells. CD184 also shifted towards heavier fractions in CD44v6kd lysates. A representative WB and the mean intensity of the signal strength evaluated by ImageJ of 3 independently performed experiments are shown (Figure 2(d)). Furthermore, anti-CD44v6, -CD104, and -CD184 efficiently coimmunoprecipitated Tspan8 in wt cells and TEX, whereas CD44v6 was less efficiently precipitated by anti-CD104 in cells and TEX and by anti-Tspan8 in TEX (Figure 2(e)). Thus, due to the engagement of CD44v6 in Tspan8 transcription and the contribution of Tspan8 to Exo-biogenesis, a CD44v6kd is accompanied by reduced TEX delivery. Though the recovery of several constitutive TEX markers is not significantly affected, the recovery of CD184 and CD104 from TEX is impaired. These findings were a first indication that TEX assembly is partly dictated by CD44v6- and/or Tspan8-associated proteins.

The binding and/or uptake of CIC-TEX are prerequisites for promoting tumor cell dissemination and metastasis. Thus, it became important confirming highly CD44v6 and Tspan8 expressing PaCIC-TEX actually targeting the CD44v6kd and Tspan8kd cells.

**Figure 3:**
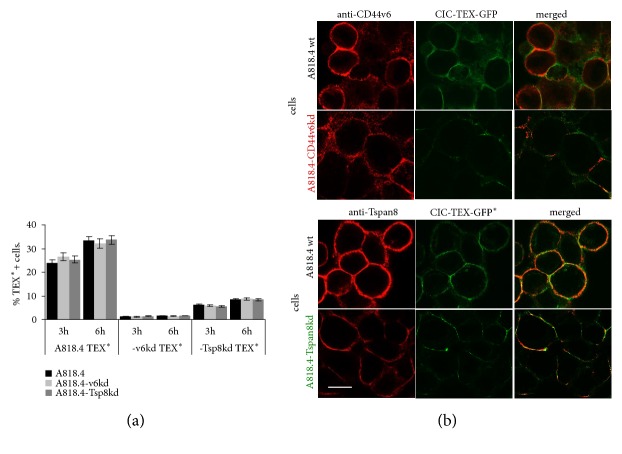
*The impact of CD44v6 and Tspan8 on TEX binding and uptake*. (a) Dio-labeled A818.4-wt, -CD44v6kd and -Tspan8kd TEX were incubated with A818.4 wt, -CD44v6kd, and -Tspan8kd cells for 3h and 6h at 37°C. After washing, uptake of Dio-labeled dye was evaluated by flow cytometry; mean ± SD of dye-labeled cells in 3 experiments is shown; (b) uptake of CIC-TEX (holoclone-derived) from GFP transfected A818.4 cells (GFP-TEX) was evaluated by confocal microscopy after counterstaining with anti-CD44v6 or anti-Tspan8. The experiment was repeated 3 times. Representative examples are shown (scale bar: 10*μ*m). A818.4-wt TEX are taken up by -wt, -CD44v6kd, and -Tspan8kd cells; instead A818.4-CD44v6kd and -Tspan8kd TEX are poorly taken up, indicating that CD44v6kd and Tspan8kd cells express ligands for A818.4-wt TEX binding, but CD44v6kd- and Tspan8kd-TEX miss receptors/receptor complexes. Uptaken TEX preferentially colocalize with Tspan8.

After dye-labeled A818.4 TEX were incubated for 3h or 6h with A818.4-wt, -CD44v6kd, and -Tspan8kd cells, uptake was evaluated defining the percent of dye-labeled cells by flow cytometry. A818-wt, -CD44v6kd, and -Tspan8kd cells took up wt-TEX with comparable efficacy. Instead, A818.4-CD44v6kd- and A818-Tspan8kd-TEX are not or poorly ingested (Figure 3(a)). To avoid potential skewing of the results due to TEX labeling, the experiment was repeated with TEX derived from GFP transfected A818.4 holoclones. Confocal microscopy confirmed unimpaired integration of “GFP-TEX” in wt, CD44v6kd, and Tspan8kd cells with a notable colocalization of GFP with Tspan8 (Figure 3(b)). These findings imply TEX CD44v6 and Tspan8 expression being a prerequisite for efficient binding/uptake but not for target cell uptake, although target cell Tspan8 expression may be advantageous. Importantly, unimpaired binding/uptake of CIC-TEX by CD44v6kd and Tspan8kd cells reassures CIC-TEX crosstalking with “Non-CIC” targets.

Having affirmed the impact of CD44v6 on Tspan8 transcription and the engagement of both CIC-markers on TEX delivery and target cell binding/uptake, we evaluated the impact of CD44v6, Tspan8, and associated molecules on the TEX composition.

### 3.3. CD44v6-Linked Protein Recovery in Cells and TEX

CD44v6 is associated with a wide range of transmembrane molecules and via the cytoplasmic tail with cytoskeleton linker proteins and nonreceptor signaling molecules [8]. Part of CD44v6 is Tspan8-associated and is recovered in internalization-prone TEM [6]. A proteome analysis of anti-CD44 precipitates in wt and CD44v6kd cells versus TEX could elucidate selective CD44v6 contributions to the TEX proteome profile.

A818.4-wt and -CD44v6kd cell and TEX were mildly lysed to avoid destroying loose associations. Lysates were precipitated with anti-CD44 and analyzed by Nano-LC-ESI-MS/MS spectrometry. Anti-CD44 precipitated 258 and 278, respectively, proteins in A818.4-wt cells and TEX, from which 99 and 75, respectively, were also recovered in CD44v6kd cells and TEX. Similar results were obtained with Capan1, with 65 from 202 precipitates in wt cells being recovered in CD44v6kd cells and 80 from 248 wt TEX precipitates in CD44v6kd TEX (Tables S3A-S3C, Figure S1A). The finding indicated an unexpectedly high number of proteins being CD44v6-associated in cells and TEX. However, the relative contribution of CD44v6-associated proteins not differing between cells and TEX argues against CD44v6 being actively engaged in the transfer of associated proteins.

Panther pathway analysis of molecular functions of CD44- versus CD44v6-associated molecules in cells revealed no major differences, only molecular function regulating and structural proteins being slightly increased in CD44v6 precipitates. In TEX, molecular function regulating and transport proteins coimmunoprecipitated preferentially with CD44v6. Furthermore, catalytic active proteins coimmunoprecipitated more and structural molecules less frequently with CD44v6 than CD44 in A818.4 and Capan1 cells and TEX (Figure S1B). Finally, analyzing coimmunoprecipitating molecules according to selected protein classes revealed nucleic acid binding proteins coimmunoprecipitating less frequently and signaling molecules more abundantly with CD44v6 than CD44 (Figure S1C).

The Panther pathway analysis overview confirmed the comparability of CD44-/CD44v6-associated proteins in two PaCa lines and indicated subtle differences between cells and TEX as well as between CD44v6- versus CD44-associated proteins. IPA-based Reactome enrichment analysis indicated, in addition, a striking overrepresentation of transport, signal transduction, and splicing engaged proteins in CD44v6 coimmunoprecipitates. These features are particularly relevant in TEX biogenesis or are central in gaining metastatic capacity. This led us to conduct a detailed analysis of traffic-, signal transduction-/EMT-engaged and RNA-processing coimmunoprecipitating proteins.

**Figure 4:**
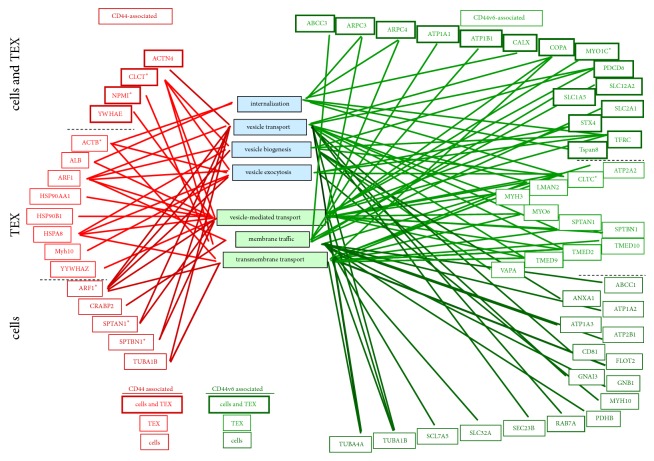
*Assignment of CD44- and CD44v6-associated molecules to traffic engagement.* Coimmunoprecipitating molecules in cells and TEX were grouped according to proteins engaged in vesicle biogenesis and transport (Panther/Reactome): CD44-associated in cells (dark red), TEX (red), cells, and TEX (red, dark red framed) and CD44v6-associated in cells (dark green), TEX (green), cells, and TEX (green, dark green framed). Full names of synonyms: Table S6. With the exception of vesicle exocytosis, traffic-associated molecules are more frequently associated with CD44v6 than CD44 in cells and TEX.

We started with proteins implicated in trafficking, which play a major role in TEX biogenesis. Proteins were categorized according to functions in internalization, vesicle-transport, -biogenesis, -exocytosis, vesicle-mediated transport, membrane traffic, and transmembrane transport. Molecules engaged in all of these processes were associated with CD44v6 to a significantly higher degree than with CD44, with the exception of vesicle exocytosis. There is no hint for a selective association of CD44/CD44v6 with traffic-engaged proteins in TEX compared to cells (Figure 4). We interpret the finding indicating the preferred association of traffic-engaged molecules with CD44v6 to be a sequel of the association of CD44v6 with Tspan8 in TEM and during EE traffic. The exception of no enrichment of exocytosis-engaged proteins in the CD44v6 precipitates is not opposing the assumption. To our knowledge, Tspan8 does not contribute to the traffic of MVB towards the cell membrane and the vesicle release (MZ, unpublished).

**Figure 5:**
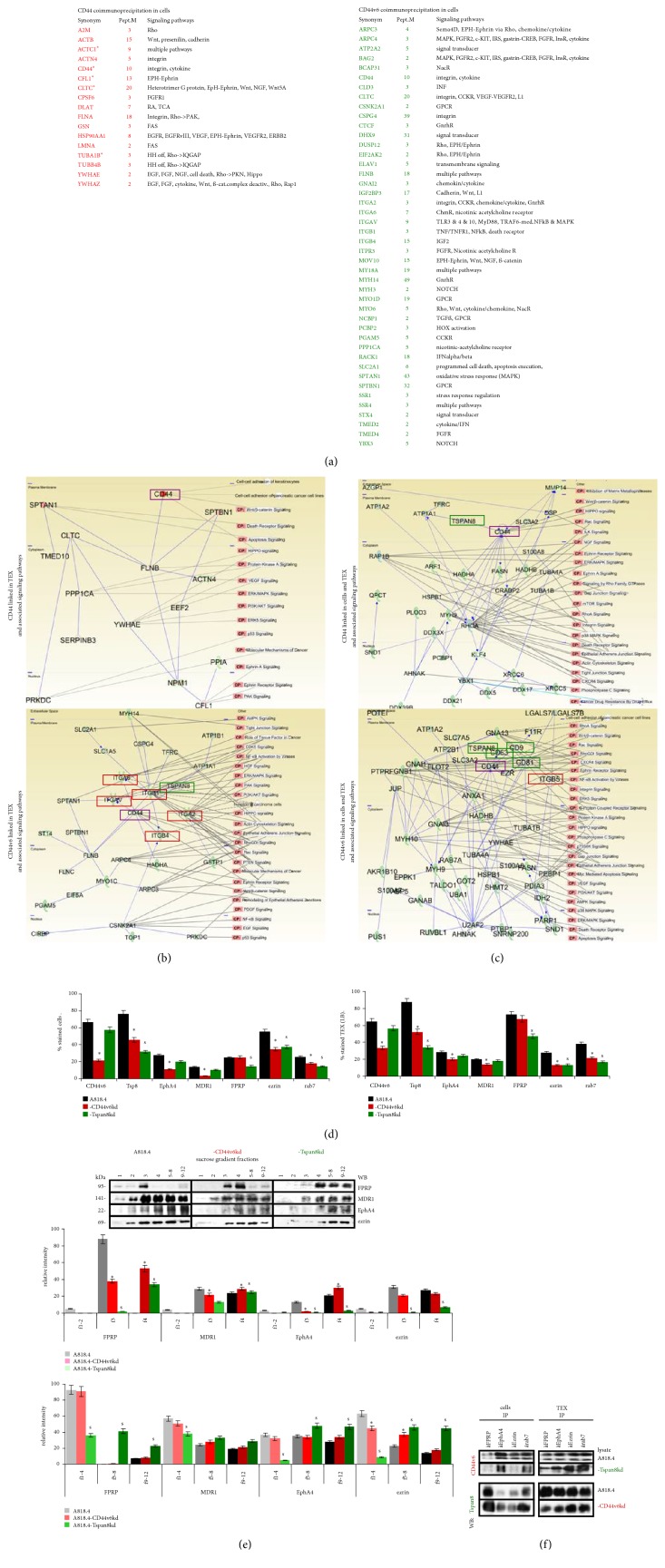
*Assignment of signaling molecules associated with CD44 and CD44v6 in cells and TEX.* (a) List of signal transduction-engaged proteins in cells coimmunoprecipitating with CD44 (red) or CD44v6 (green) (Reactome analysis); STRING pathway analysis of CD44- and CD44v6-associated proteins in (b) TEX and (c) cells and TEX (CD44 framed violet, integrins: framed green, tetraspanins: framed red; full names of synonyms: Table S6). (d-f) Tspan8-dependence of CD44v6-associated molecules on recovery in TEM and transfer into TEX. (d) Flow cytometry analysis of CD44v6- and/or Tspan8-associated molecules in cells and TEX; mean ± SD of 3 experiments; significant differences between wt and CD44v6kd cells/TEX: *∗*, significant differences between wt and Tspan8kd cells/TEX: s. (e) WB of wt, CD44v6kd, and Tspan8kd cell lysates after sucrose gradient fractionation and blotting with the indicated antibodies; a representative example and relative band intensity ± SD of 3 experiments evaluated by ImageJ are shown; significant differences between wt and CD44v6kd lysates: *∗*, significant differences between wt and Tspan8kd lysates: s. (f) WB of wt, CD44v6kd, and Tspan8kd cell and TEX lysates with anti-CD44 and anti-Tspan8 after coimmunoprecipitation with the indicated antibodies. A representative example of 3 assays is shown. A significantly higher number of signaling-engaged proteins coimunoprecipitated with CD44v6 than CD44 in cells and TEX. Dominant in CD44v6-precipitates are components of integrin, EPH-Ephrin, cytokine, GPCR, and NOTCH signaling pathways. The majority of proteins, particularly those recovered in TEX, also associate with Tspan8. This suggests that the association with CD44v6 partly relies on Tspan8-mediated TEM recruitment.

Coimmunoprecipitation of signaling molecules with CD44 and CD44v6 was analyzed by Reactome for precipitates in cells; only the coimmunoprecipitating proteins and their involvement in signaling pathways are shown (Figure 5(a)). STRING pathway analysis was used for the analysis of CD44/CD44v6-coimmunoprecipitating proteins in TEX or TEX and cells and includes pathway components not directly coprecipitating with CD44/CD44v6 (Figures 5(b) and 5(c)). These analyses uncovered a significantly higher number of signaling pathway-engaged proteins associated with CD44v6 than CD44 in cells and this difference is even more pronounced in TEX. Dominant in CD44v6-precipitates are components of integrin, EPH (Ephrin receptor)-Ephrin, cytokine, GPCR (G protein-coupled receptor), and NOTCH signaling pathways. Though engagement of these pathways is also seen in CD44 precipitates, here mostly 1 or 2 precipitating proteins are involved, whereas CD44v6 precipitates frequently reveal 10 or more proteins.

This led us to wonder whether the abundance of CD44v6-coprecipitating signal transduction-engaged molecules in TEX is due to their recruitment into TEM. To answer this question, we compared the recovery of directly Tspan8-associated FPRP (prostaglandin F2 receptor negative regulator), CD44-associated MDR1 (multidrug resistance 1) and EphrinB4, and both Tspan8- and CD44-associated ezrin and vesicle-traffic-engaged rab7 [16, 22, 24, 35] by flow cytometry in cells and TEX. Flow cytometry confirmed reduced EphA4 and MDR1 expression in CD44v6kd cells and less efficiently TEX. Tspan8-linked FPRP expression was downregulated in Tspan8kd cells and TEX. The expression of ezrin and rab7 was reduced in A818.4-CD44v6kd and -Tspan8kd cells and TEX (Figure 5(d)). Analyzing the recovery in sucrose gradient density fractions of wt, CD44v6kd and Tspan8kd cells revealed a strong shift of FPRP but also of ezrin towards heavier fractions in Tspan8kd cells and a slight shift of MDR1 and EphA4 towards higher density fractions in CD44v6kd and more pronounced Tspan8kd cells (Figure 5(e)). Finally, CD44v6 was reduced in anti-FPRP, -ezrin, and slightly -EphA4 precipitates of Tspan8kd cell lysates. Tspan8 recovery was also reduced in anti-EphA4 and -ezrin precipitates of wt and CD44v6kd cells, which might be a consequence of the CD44v6-association with Tspan8. Differences in coimmunoprecipitation in TEX were less pronounced. This was mostly apparent in the coprecipitation of CD44v6 and Tspan8 with anti-FPRP and anti-EphA4 precipitates, which displayed weakened signals with CD44v6 and Tspan8, respectively, in Tspan8kd and CD44v6kd TEX lysates (Figure 5(f)). These results indicate that (i) the strongly pronounced coimmunoprecipitation of signaling molecules with CD44v6 in TEX relies, at least partly, on the association of CD44v6 with Tspan8 in TEM and (ii) the impact of CD44v6 on Tspan8 transcription affects the recruitment of CD44v6-associated signaling molecules into TEX.

**Figure 6:**
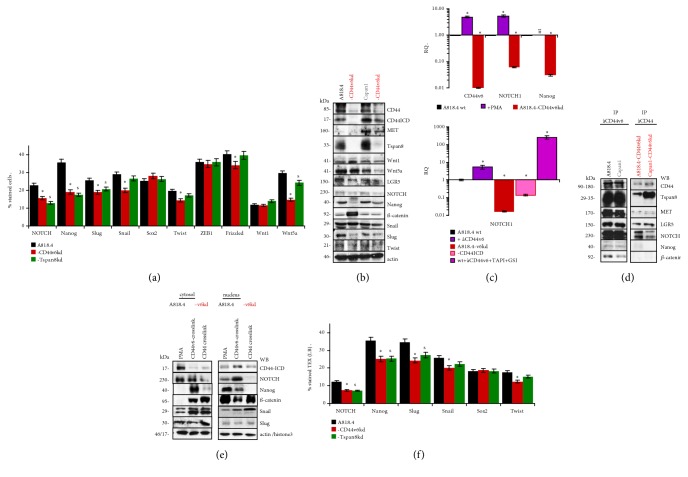
*The association of EMT-related transcription factors with CD44 and CD44v6*. (a) Flow cytometry analysis of EMT-related transcription factors in A818.4 wt, -v6kd, and Tspan8kd cells; mean ± SD of 3 experiments; significant differences between wt and CD44v6kd cells: *∗*, significant differences between wt and Tspan8 cells: s; (b) WB of EMT transcription factors in wt and CD44v6kd cells; a representative example of 3 repetitions is shown; (c) qRT-PCR of CD44v6, NOTCH, and Nanog in A818.4-wt and -CD44v6kd cells with dependence on stimulation by PMA or CD44v6-crosslinking or CD44ICD transfection or in the presence of TAPI and GSI to block CD44ICD liberation; RQ values (mean ± SD of 3 triplicates, 2 repetitions); significant differences to unstimulated wt cell mRNA: *∗*, nt: not tested; (d) WB of EMT transcription factors coimmunoprecipitating with CD44v6 in wt cells and with CD44 in CD44v6kd cells; (E) WB of EMT-related transcription factors in the cytosolic and the nuclear fractions of wt and CD44v6kd cells, which were stimulated by PMA or CD44v6- (wt cells)/CD44 (CD44v6kd cells)-crosslinking; (e and d) representative examples of 3 repetitions; (f) Flow cytometry of EMT-related transcription factor expression in wt, CD44v6kd, and Tspan8kd TEX; mean ± SD (3 assays) of stained LB; significant differences between wt and CD44v6kd TEX: *∗*, significant differences between wt and Tspan8 TEX: s. Several EMT-related transcription factors are associated with CD44v6 in cells and TEX, where particularly NOTCH expression becomes upregulated by CD44v6 stimulation. CD44v6 stimulation also supports nuclear transfer of associated EMT transcription factors.

CD44 was described to be associated with LGR5 (leucine rich repeat containing G protein-coupled receptor 5) that supports Wnt signaling [36] and to contribute to NOTCH and Nanog transcription [37]. With EMT playing a major role in tumor progression [13], we screened for a selective contribution of CD44v6. Flow cytometry revealed reduced expression of the EMT-related transcription factors NOTCH, Nanog, Slug, and Wnt5a in CD44v6kd and Tspan8kd cells. The expression of Snail, Twist, and Frizzle was lower in CD44v6kd cells (Figure 6(a)). WB confirmed downregulation of NOTCH and Nanog but increased *β*-catenin (*β*-cat) expression in CD44v6kd cells (Figure 6(b)). qRT-PCR also pointed towards reduced Nanog and NOTCH mRNA recovery in CD44v6kd cells and upregulated CD44v6 and NOTCH expression in PMA-treated wt cells. As CD44 was reported to promote NOTCH transcription [38] and NOTCH mRNA became upregulated by PMA-treatment, we searched for cotranscription factor activity of the CD44ICD. As described above for Tspan8, NOTCH transcription was supported by membrane anchored but not nuclear CD44ICD (Figure 6(c)). Co-IP with anti-CD44v6 in A818.4- and Capan1-wt cell lysates and with anti-CD44 in A818.4- and Capan1-CD44v6kd lysates showed MET, Tspan8, LGR5, and NOTCH being preferentially CD44v6-associated. Nanog and *β*-cat weakly coimmunoprecipitated with CD44v6 but not CD44 (Figure 6(d)). Slug, Snail, Sox2, Twist, and ZEB1 did not coimmunoprecipitate with CD44v6 or CD44 (data not shown). Thus, the reduction of EMT-related transcription factors in CD44v6kd cells involves those associated with CD44v6. This has consequences on the nuclear transfer. Snail and Slug were recovered in the nuclear fraction independent of CD44 crosslinking. Nuclear recovery of *β*-cat increased after PMA stimulation and CD44 crosslinking, which indicates that it is CD44v6-independent. A translocation of NOTCH and Nanog is only supported by CD44v6-crosslinking (Figure 6(e)). The impact of CD44v6 on EMT gene recovery was relevant for TEX. NOTCH, Nanog, and Slug expression was impaired in CD44v6kd and Tspan8kd TEX (Figure 6(f)).

**Figure 7:**
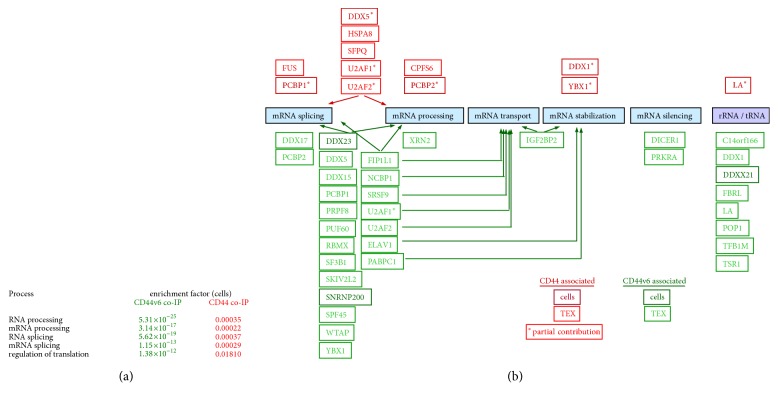
*CD44v6 and mRNA processing*. (a) Enrichment factors for some proteins engaged in RNA-related processes in CD44v6 versus CD44 coimmunoprecipitating cell lysates. (b) Reactome analysis of CD44s-associated (cells: dark red, TEX: red) and CD44v6-associated (cells: dark green, TEX: green) mRNA processing proteins grouped according to splicing, processing, transport, stabilization, and silencing or rRNA and tRNA processing is shown (full names of synonyms: Table S6). Components of the mRNA processing machinery are most abundantly associated with CD44v6. The seemingly stronger enrichment in TEX than cells would be in line with the associations being established during ILV loading.

We also detected an intriguing association between CD44/CD44v6 and RNA processing [39, 40], the enrichment factors for cell lysates in CD44v6 compared to CD44 coimmunoprecipitates differing strikingly (Figure 7(a)). According to Reactome analysis, CD44- or CD44v6-associated mRNA-regulating proteins were grouped into engagement in splicing, processing, transport, stabilization, and silencing. A last category comprised proteins engaged in rRNA and tRNA processing. With few exceptions, only CD44v6 coimmunoprecipitates with components of the RNA processing machinery. The majority of proteins were involved in splicing and processing and, less frequently, mRNA transport. Most prominent were coimmunoprecipitation with Dicer (Endoribonuclease Dicer) and several DEAD (DEAD-box helicase) box family members, coregulators of transcription, splicing, and RNA processing. Notably, many of these proteins were more prominently enriched in TEX, suggesting an active recruitment during ILV (intraluminal vesicle) loading and integration into MVB (Figure 7(b)).

In summary, CD44v6 associates with vesicle and vesicle-mediated trafficking molecules, in which associations apparently are linked to Tspan8. CD44 has a strong impact on signal transducing molecules including some EMT-related transcription factors and on RNA processing, the latter being CD44v6-dependent. The molecules being recovered primarily in TEX rather than cells indicate that the process likely takes place during vesicle integration into MVB. Thus, CD44v6 may well affect the miRNA profile.

### 3.4. CD44v6 and the miRNA Profile

The coimmunoprecipitation of CD44v6 with Dicer and several translation engaged proteins suggests that CD44 plays a role in miRNA processing, a finding in line with previous reports that CD44v6 affects the generation of miRNA [30, 39, 40]. This was evaluated by DS of miRNA in A818.4-wt, -CD44v6kd, and -Tspan8kd cells and TEX and was controlled through a DS mRNA analysis based on the recovery of predicted target mRNA in the corresponding populations.

In advance, we ranked the most abundant miRNA in the two PaCa lines A818.4- and Capan1-wt cells and TEX to obtain a first overview of PaCa miRNA profiles. With few exceptions, A818.4 and Capan1 cells and TEX share the 26 most prominent miRNA (Table S4); only 3 miRNA (miR-27b-3p, miR-10a-5p, and miR-1273g-3p) differ significantly in signal strength between Capan1- and A818.4-wt cells. No significant differences were seen in the 26 most abundant miRNA between A818.4- and Capan1-wt TEX (Figures S2A and S2B). However, miRNA recovery differs between cells and TEX as demonstrated for A818.4-wt cells versus TEX. The most prominent reductions in TEX compared to cells were observed for miR-4284, miR-499a-5p, miR-8072, and miR-150-5p. miR-1246, miR-192-5p, miR-1290, miR-215-5p, and miR-7107-5p were strongly enriched in wt TEX compared to cells. We confirmed this for selected miRNA by qRT-PCR (Figures S2C-S2E).

Further pursuing whether abundant miRNA in A818.4-wt TEX can be expected to affect PaCa-relevant mRNA, predicted targets were searched for by the microrna.org, DianaTools, and targetscan.org programs and selected by IPA for being targets of miRNA with an impact on PaCa. Six miRNA were recovered in cells and TEX and 4 in either cells or TEX that had high fidelity targets engaged in PaCa growth and progression, an impact on proliferation being dominating in cells and TEX. miRNA affecting motility were only recovered in cells and miRNA regulating apoptosis only in TEX (Figure S2F). The finding indicates selective recruitment of miRNA into TEX. To test this assumption, we selected predicted targets for miRNA distinctly expressed in cells versus TEX and used IPA to query whether they were involved in functions relevant to PaCa. Only two PaCa-related miRNA were more abundant in cells than TEX; both of these targeted the transcription factor GLI1 (GLI family zinc finger 1). However, 10 miRNA, which predominantly regulate proliferation and apoptosis, were recovered at a higher level in TEX than cells. Notably, predicted targets included signal transducing molecules and EMT-related transcription factors (Figure S2G).

This prescreening indicated that several miRNA enriched in PaCa cells or TEX might have relevance for PaCa progression. This raised the question whether Tspan8 and/or CD44v6 contribute to miRNA maturation/recruitment into TEX. We approached this by comparing the miRNA profiles of wt versus CD44v6kd and Tspan8kd cells and TEX. Though the latter may be skewed by the concomitant impact of CD44v6 on Tspan8 transcription, the Tspan8kd might indicate whether changes were truly related to Tspan8.

**Figure 8:**
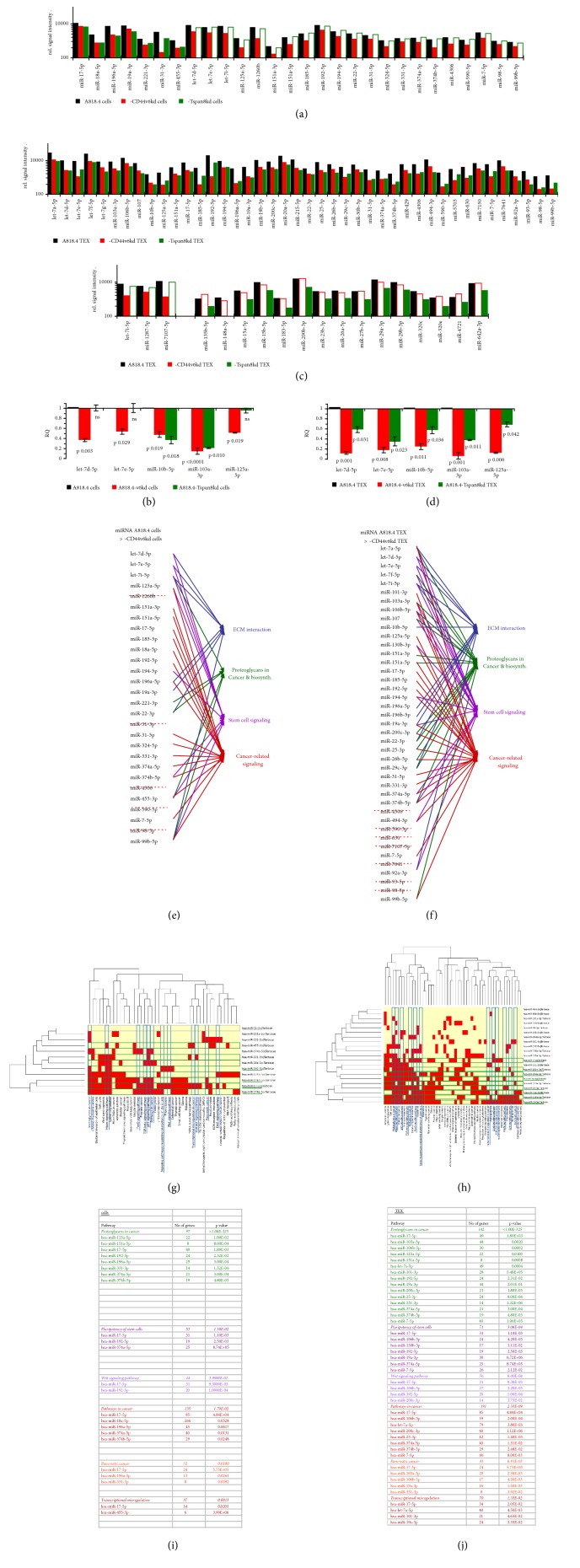
*Analysis of miRNA significantly differing between wt and CD44v6kd or Tspan8kd PaCa cells and TEX*. (a) miRNA that is significantly higher in A818.4-wt than -CD44v6kd and/or -Tspan8kd cells and (c) miRNA that is significantly higher in A818.4-wt than -CD44v6kd and/or -Tspan8kd TEX; no significant differences: empty bar; (b, d) qRT-PCR examples of miRNA differing between wt and CD44v6kd and/or Tspan8kd cells or TEX (RQ values ± SD of 3 replicates; p values are indicated; the experiments were repeated 2 times). (e, f) IPA-based Reactome analysis was used for correlating miRNA with protein expression according to mRNA predictions by the miRNA, DIANA, and target scan databases. (e) miRNA higher in A818.4-wt than -CD44v6kd cells and their engagement in ECM interactions, proteoglycans in cancer, stem cell signaling, and cancer-related signaling are shown. (f) miRNA more abundant in wt than CD44v6kd TEX was analyzed as in (e); (e, f) crossed miRNA (4 miRNA higher in wt than CD44v6kd cells, 7 miRNA higher in wt than CD44v6kd TEX) are not engaged in the listed cancer-related activities. (g, h) Clustered heatmap analysis (p values under the selected threshold and 1) of the potential impact of miRNA that is reduced in CD44v6kd cells (g) and TEX (h) on predicted targets engaged in cancer relevant activities; pathway of interest is framed and underlined in blue; most frequently engaged miRNA are framed and underlined in green; in (g) miR-31-3p (framed and underlined in grey) was included as a negative control not affecting the pathways of interest. (i, j) Number of genes and significance of overrepresentation of predicted mRNA of selected CD44v6-dependent miRNA in cells (i) and TEX (j). Only CD44v6 affects miRNA processing and/or recruitment into ILV. The majority of miRNA distinctly processed with dependence on CD44v6 have predicted targets in cancer-related proteoglycans as well as in maintaining stemness and cancer-related signaling. Statistical evaluation of predicted genes overrepresentation confirmed a stronger impact on TEX than on cell miRNA.

The CD44v6kd line exhibited a significant reduction of 28 miRNA, 7 of which were also reduced in the Tspan8kd line. There was no selective reduction in the Tspan8kd line, confirmed by qRT-PCR (Table S5A, Figures 8(a) and 8(b)). Instead, 41 miRNA were recovered at a reduced level in CD44v6kd- and Tspan8kd-TEX; 15 miRNA were reduced only in Tspan8kd-TEX and 3 only in CD44v6kd-TEX, with qRT-PCR confirming the DS analysis for selected examples (Table S5B, Figures 8(c) and 8(d)). This indicates that only cellular CD44v6 is engaged in miRNA processing, while Tspan8 likely contributes to the recruitment into TEX.

To determine whether reduced miRNA recovery in CD44v6kd cells and TEX affects CIC-relevant features, we categorized the miRNA into clusters of cancer-related activities. These comprised ECM interactions, proteoglycans in cancer, signaling in stem cells, and cancer-related signaling. Of 28 miRNA whose expression was higher in A818.4-wt than -CD44v6kd/-Tspan8kd cells, 23 miRNA had targets affecting at least one cancer-related activity (Figure 8(e)). Stem cell and cancer-related signaling were affected the most frequently. This suggests that CD44v6 might preferentially process miRNA related to the regulation of signaling, the predicted targets with relevance for cancer-related signaling being listed (Figure S3A). Of the 41 miRNA reduced in CD44v6kd and Tspan8kd TEX, 34 had predicted targets engaged in cancer-related activities, predominantly in proteoglycan expression and biosynthesis and in stem cell and cancer-related signaling (Figure 8(f)). Because of the abundance of signal transduction engaged predicted targets, we restricted the search to predicted targets involved in EMT-induction, which is promoted by CIC-TEX in nonmetastasizing cancer cells [41]. Despite this restriction, the CD44v6-dependent miRNA enriched in TEX covered a large range of predicted targets (Figure S3B). We used IPA-based KEGG analysis to assign them to specific signaling pathways. The predicted targets were engaged in multiple pathways with no obvious prevalence. This accounted for targets of CD44v6-dependent miRNA in cells and TEX. Nonetheless, and despite some overlap, a miRNA reduction affected a far denser network of predicted signaling molecules in CD44v6kd TEX than in cells (Figures S3C and S3D). Restricting the analysis to the topics of particular interest as shown in Figures 8(e) and 8(f) unraveled miRNA 17-5p, followed by miR-192-5p, -196a-5p, and -374-5p to be particularly engaged in CD44v6-dependent miRNA regulation in mRNA signaling in cells (Figure 8(g)). In TEX, most prominently miR-17-5p followed by miR-7-5p, -19a-3p, -25-3p, -103a-3p, -106b-5p, -200c-3p, -374a-5p, and let-7a-5p are engaged in these processes (Figure 8(h)). KEGG-based network analysis for selected miRNA confirmed the higher connectivity in TEX than cells and disclosed a strong impact on proteoglycans and pathways in cancer (Figures 8(i) and 8(j)).

Reduced miRNA recovery in CD44v6kd cells is in line with several splicing factors and dicer coimmunoprecipitating with CD44v6. Due to the Tspan8 engagement in vesicle-biogenesis, CD44v6kd deficits are transferred into TEX. The analysis of predicted targets and their engagement in cancer related activities and signaling networks implies that the reduced recovery in CD44v6kd and Tspan8kd cells may well affect the tumorigenicity of CD44v6kd PaCa cells. Notably, proteoglycans were most frequently, but stem cell maintenance and signaling pathways in cancer are also frequently affected by CD44v6-dependent miRNA in cells and TEX.

These suggestions demand for a scrutinized evaluation of a correlation between the TEX miRNA profile and the availability of the predicted mRNA targets in (nonmetastatic) CD44v6kd and Tspan8kd cells, which remains to be approached. As a prerequisite, mRNA DS analyses were performed of wt, CD44v6kd, and Tspan8kd cells and CIC-TEX.

Briefly, DS showed over 6000 mRNA with a signal strength >1000 inA818.4-wt, -CD44v6kd, and –Tspan8kd cells as well as A818.4 TEX. An overview on molecular functions of RNA (signal strength >1000) revealed an abundance of binding and catalytic activity engaged RNA but no obviously significant differences between A818.4-wt, -CD44v6kd, and -Tspan8kd cells. This also accounted for the comparison between wt cells and TEX (Figure S4A). Searching for RNA with significant differences in signal strength indicated that compared to wt cells 7.3% of mRNA were significantly reduced and 2.2% of mRNA were significantly higher in CD44v6kd than wt cell lysates. A Tspan8kd was accompanied by 6.0% reduced and 2.2% upregulated RNA compared to wt cell RNA. Finally, we compared the two kd RNA profiles. The CD44v6kd exhibited a 7.8% upregulation and 10.2% downregulation in mRNA compared to the Tspan8kd. When mRNA recovery from cells was compared to that of TEX, 15.2% of mRNA were higher and 12.0% lower in cells (Figure S4B). Distinctly recovered mRNA showed a slight increase in binding and signal transducing molecules in CD44v6kd compared to wt cells and a higher percentage of catalytic RNA. The percentage of transcription/translation-engaged mRNA was lower in Tspan8kd than wt cells. Comparing the kd cells confirmed a higher recovery of signal transducing and transcription/translation-engaged mRNA but a lower recovery of transporters in CD44v6kd than Tspan8kd cells (Figure S4C). The comparison of molecular functions in cells versus TEX revealed a reduction in signal transducing, transcription regulating, and transporter mRNA and an increase in structural activity-engaged mRNA in TEX compared to cells, all differences being minor (Figure S4D).

**Figure 9:**
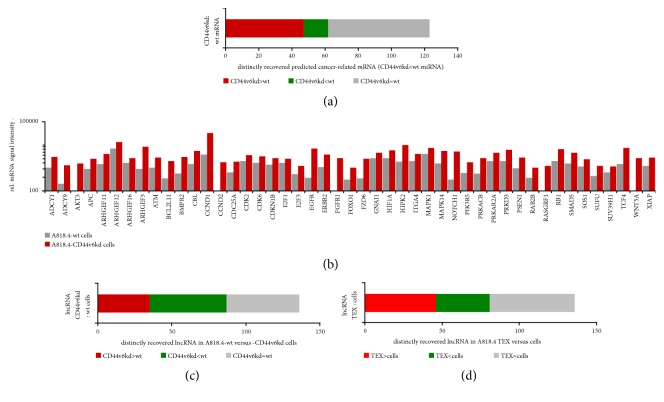
*Correlation of mRNA with miRNA and lncRNA recovery*. (a) Correlation of distinctly recovered mRNA in CD44v6kd versus wt cells (≥1.5-fold) with miRNA lower in CD44v6kd than in wt cells. (b) Signal strength of the 47 mRNA with higher recovery in CD44v6kd than in wt cells (full name of synonyms: Table S6). Comparison of lncRNA recovery in (c) wt versus CD44v6kd cells and (d) wt cells versus TEX, with distinct recovery being defined as ≥1.5-fold difference of the signal strength revealed by DS. Higher mRNA recovery in CD44v6kd cells displaying reduced miRNA recovery was only observed in 38% of the predicted targets, which might be interpreted as random. A potential impact of CD44v6 on lncRNA waits for elaboration. So far, we noted significant differences in lncRNA recovery between wt versus CD44v6kd cells as well as between wt cells versus TEX; the impact on miRNA targets remains to be explored.

Finally, in view of the abundance of miRNA being engaged in PaCa and stem cell signaling and the suggested importance of transferred TEX miRNA in transiently driving the malignancy of non-CIC targets, we expected to obtain a hint towards the efficacy of transferred miRNA by correlating miRNA higher in wt than CD44v6kd cells with the mRNA level of predicted targets outlined in Figure S3. Predicted mRNA (signal strength of at least one component >1000) was sorted according to ≥1.5-fold differences between wt and CD44v6kd cells. Higher mRNA recovery in CD44v6kd than wt cells was only seen in 38% of the predicted targets, the signal strength of these 47 mRNA in A818.4-CD44v6kd and -wt cells being shown. Instead, expression level of predicted mRNA mostly did not significantly differ between wt and CD44v6kd cells and 15 mRNA were opposing expectation lower in CD44v6kd than wt cells (Figures 9(a) and 9(b)).

Though the corresponding analysis for miRNA affecting distinct metastasis-related activities that are significantly upregulated or downregulated in dependence on CD44v6 remains to be evaluated, the random correlation between miRNA and predicted mRNA recovery was unexpected. It might point towards mRNA silencing requiring very high level expressed miRNA or a concomitant attack by several miRNA. The incorporation of miRNA in regulatory circuits could be an alternative, not mutually exclusive explanation. One such option could be lncRNA (ceRNA), which sponges cellular miRNA.

According to the present state of knowledge, we recovered 136 lncRNA in A818.4 cells; 35 of these were expressed at higher levels in CD44v6kd than wt cells and the expression of 52 was lower (Figure 9(c), Figures S5A and S5B). The recovery of lncRNA also differed significantly between A818.4-wt cells versus TEX; 46 lncRNA were detected at higher levels in cells than TEX; the recovery of 35 was lower (Figure 9(d), Figures S5C and S5D). This preliminary screen indicates a potential contribution of lncRNA to CIC-TEX-promoted tumor progression, but the finding awaits verification. LncRNA have been implicated in regulating chromosome structure as well as showing effects on DNA transcription and on RNA, miRNA, and proteins. However, functional activities of most lncRNA are unknown or only selected activities are described (Table S7). Thus, progress in unraveling the functional relevance of cellular and exosome lncRNA in cancer progression is urgently anticipated and may shed light on the correlation between miRNA and predicted target mRNA recovery.

Briefly, the analyses of CD44v6- and Tspan8-dependent changes in protein, miRNA, and mRNA recovery point towards a dominant role of the PaCIC marker CD44v6 in shaping the armament of both cells and TEX. The impact of Tspan8 relies on clustering proteins in invagination prone cell membrane domains, which facilitates the transfer into TEX and the communication between TEX and targets. CD44v6 being one of the Tspan8-associated molecules, cellular activities of CD44v6 are efficiently transferred into TEX.

## 4. Discussion

CIC-TEX binding/uptake by non-CIC is suggested to be the initiating trigger towards a more malignant phenotype. Aiming for interrupting this crosstalk, we searched as a first step for tumor progression-facilitating features of the CD44v6 CIC-biomarker. The selection was based on CD44v6 associating and cooperating with a multitude of signaling molecules, its disputed engagement in shaping the miRNA profile, and, in gastrointestinal cancer, its partial recruitment into TEM, where it associates with Tspan8. As Tspan8 is involved in TEM-derived exosome biogenesis and binding, CIC-related activities of CD44v6 may efficiently be transferred into TEX via Tspan8. We will discuss our interpretation on the lead role of CD44v6 in promoting PaCa progression via TEX with emphasis on open questions waiting to be answered.

### 4.1. PaCIC-TEX Are Transferred into Non-CIC

Non-CIC are characterized besides other features by not or poorly expressing CIC-biomarkers including CD44v6 and Tspan8. Thus, message transfer from CIC-TEX requires target cell independence of CIC-biomarker expression. We already demonstrated that CIC-TEX binding is greatly facilitated by Tspan8 protein clusters in TEX which bind to clustered proteins on the target membrane. The latter can be TEM-independent [29]. This also accounts for CIC-TEX binding to CD44v6kd and Tspan8kd human PaCa lines. Thus, the prerequisite of CIC-TEX CD44v6 and Tspan8 contributing to the communication with non-CIC was fulfilled.

### 4.2. Links between the CIC-Markers CD44v6 and Tspan8 and CD44ICD Cotranscription Factor Activity

In PaCa Tspan8 is associated with several CIC markers like *α*6*β*1, CD104, EpCAM, CXCR4, and CD44v6 [6], a reduction of Tspan8 expression being frequently associated with reduced expression of the associated molecules. We recently noticed an additional link, CD44v6 promoting Tspan8 transcription [10]. The cytoplasmic tail of CD44 acting as a cotranscription factor [14], we elaborated the underlying mechanism. CD44v6-crosslinking promotes CD44 internalization and cleavage. However, transient transfection with the CD44ICD promoted CD44, but not Tspan8 transcription. As prohibiting CD44 cleavage was accompanied by increased Tspan8 transcription, CD44ICD does not act as a cotranscription factor for Tspan8. Similar findings accounted for CD44v6-promoted NOTCH1 transcription. Thus, we confirmed the cotranscription factor activity of the CD44ICD for CD44 but not for Tspan8 and NOTCH. Though at variance with reports describing nuclear translocation of the CD44ICD promoting stemness factor activation [14], one should keep in mind that EMT transcription factor activation is controlled at multiple levels with several not yet fully elucidated feedback loops [42, 43]. Nonetheless, the findings sustain membrane-anchored CD44ICD assisting Tspan8 and NOTCH transcription. With regard to the analysis of CD44v6 activities in tumor progression, the links between CD44v6 and Tspan8 can impede an unequivocal assignment. To cope with this drawback, Tspan8kd cells and TEX were included. This allowed, at least, defining CD44v6-independent but Tspan8-dependent activities.

### 4.3. Analyzing the Impact of CD44v6 on Tumor Progression by Coimmunoprecipitation, miRNA, and mRNA: Limits and Prospects

CD44v6 is a transmembrane molecule well described for its multitude of binding partners and binding-initiated activation and signal transduction. The focus of the presented data relies on the analysis of Nano-LC-ESI-MS/MS-defined coimmunoprecipitating proteins. The Nano-LC-ESI-MS/MS was performed with wt and CD44v6 kd cells and TEX of two PaCa lines (A818.4, Capan1) and a colon cancer line (SW480), data being deposited at Functional Proteome Analysis (files: ZW2612, ZW2484, SH: 2726), German Cancer Research Center, Heidelberg. We frequently show only the analysis of the A818.4 line for clarity of presentation. However, all proteome data were evaluated and revealed in >90% to 98% concordant results. For selected proteins confirmation is provided by flow cytometry and WB including coimmunoprecipitation and/or sucrose density gradient fractionation, experiments being performed 3 times.

Stimulated by an unexpected recovery of some proteins in coimmunoprecipitates with CD44 in CD44v6kd cells and TEX but not in coimmunoprecipitates with CD44v6 in wt cells or TEX, we proceeded with DS miRNA analysis of A818- and Capan1-wt, -CD44v6kd, and -Tspan8kd cells and TEX. Like for the proteome analysis, mostly the evaluation of A818.4 cells and TEX is shown, the results largely overlapping with that of Capan1 cells and TEX (deposited: ENA database, accession no. PRJEB25446). Selected examples of qRT-PCR (triplicates, 2 repetitions) confirmed the validity of the DS analysis.

Finally, a DS mRNA analysis of A818-wt, -CD44v6kd, and -Tspan8kd cells and A818.4-TEX (deposited: ENA database, accession no. PRJEB25446) served defining relevant targets out of the pool of predicted targets of distinctly regulated miRNA. So far, this topic was only approached for miRNA targeting PaCa-relevant signaling molecules. Thus, mRNA DS waits to be analyzed and controlled for additional topics of interest. This last constraint also accounts for lncRNA that according to the DS mRNA analysis were distinctly recovered in A818.4-wt versus -CD44v6kd cells or -wt cells versus TEX. We include the above-mentioned screening as a helpful hint for clarifying open questions particularly for the integration of miRNA in regulatory circuits.

Briefly, the starting material will be appropriate and sufficient for an in-depth elaboration of most aspects of CD44v6- and Tspan8-promoted tumor progression in cells and TEX, only some of which are hit by our analysis so far.

### 4.4. The Contribution of CD44v6-Associated Proteins to the Malignant Phenotype-Supporting CIC-TEX

These analyses are based on the proteins coimmunoprecipitating with CD44v6 and are discussed integrating the current state of knowledge on Exo biogenesis.

There has been an abundance of proteins coimmunoprecipitating with CD44v6 but not with the CD44 standard isoform (CD44s). We suggest this relying on the association of CD44v6 with Tspan8 in TEM [44, 45]. TEM are prone for internalization [22, 23, 46, 47] and the TEM complexes, which include CD44v6 but not CD44s [44], are maintained during TEX-biogenesis [9, 22, 23, 48]. This has consequences on the association of CD44v6 with Tspan8-associated transmembrane proteins, most prominently integrins [49]. Sucrose-gradient fractionation of selected examples of CD44v6-associated proteins in wt, CD44v6kd, and Tspan8kd cell lysates confirmed the engagement of Tspan8 by a partial shift towards heavier fractions in Tspan8kd lysates. Furthermore, co-IP of CD44v6kd and Tspan8kd cell and TEX lysates indicated a minor contribution of Tspan8 to EphA4 precipitation and of CD44v6 to FPRP precipitation in TEX. These findings strengthen the assumption that the composition of the Exo membrane forces protein interactions. However, not all proteins coimmunoprecipitating with CD44v6 in TEX are Tspan8-associated. A notable example are drug transporters, which are CD44- [16] but not Tspan8-associated. Nonetheless, MDR1 was shifted towards higher density fractions in Tspan8kd cells. We interpret the finding that outside of TEM located CD44v6 is also recruited into TEX but via different internalization-prone membrane microdomains [23, 25], which use distinct pathways for the traffic of EE towards MVB [23–25, 50]. Regardless of CD44/CD44v6, awareness of one cell delivering distinct Exo populations is an essential requisite in approving Exo activities [25].

Transport-engaged proteins are also highly enriched in CD44v6 coimmunoprecipitates of TEX lysates. Beside the engagement in internalization, Tspan8 contributes to the guidance of EE towards MVB [45, 50–53]. In fact, many vesicle biogenesis- and transport-engaged proteins coimmunoprecipitating with CD44v6 in cells and TEX are tetraspanin-associated [9, 11, 29]. This implies that coimmunoprecipitation with CD44v6 could be a sequel of the CD44v6-Tspan8 association in TEM [44]. However, some proteins engaged in vesicle exocytosis also coimmunoprecipitate with CD44. To our knowledge, Tspan8 assists the traffic of EE towards MVB but not vesicle exocytosis [46]. Fittingly, exocytosis-engaged proteins mostly are CD44s-associated. Their suggested origin from non-TEM microdomains remains to be elaborated.

Of particular importance for the contribution of TEX-CD44v6 to tumor progression was the recovery of signal transduction molecules. The CD44v6 exon product binds several growth factors such that RTK are recruited and become activated via the CD44ICD [11]. We largely missed coimmunoprecipitation with chemokines/cytokines and growth factors. This may be due to the association with these soluble factors becoming disrupted even under mild lysis conditions. Instead, the proteome analysis confirmed the association with several integrins and unraveled their activation state as evident by the coimmunoprecipitation of integrin signaling components that only become phosphorylated by activated integrins like src, FAK, and paxillin [54]. Integrin activation by associating with both CD44v6 [10] and tetraspanins were repeatedly described [22, 55, 56]. Notably, too, integrins and tetraspanins take central node positions only in CD44v6- but not CD44-precipitates. This was seen in cells but preferentially in TEX. Additional proteins selectively or preferentially coimmunoprecipitating with CD44v6 that could assist integrin activation are CLTC (clathrin heavy chain 1) and CSPG4 (chondroitin sulfate proteoglycan 4) [57, 58]. CD44v6 also associates with LGR5/LGR6, which supports Wnt binding and downstream signaling activation [12, 36]. We demonstrate that CD44v6 crosslinking, besides contributing to NOTCH1 transcription, promotes *β*-catenin-independent NOTCH and Nanog nuclear translocation. Activation of these transcription factors might be supported by CD44v6-associated MYH3 (Myosin-3) and YBX3 (Y-box-binding protein 3), which can contribute to EMT induction [14, 59–61]. Via its linkage to ERM proteins CD44 fosters cytosolic signaling molecule activation and cytoskeleton reorganization [62, 63]. Furthermore, Tspan8-associated CD44v6 encounters a large range of cytosolic signaling molecules, which attach to the inner membrane of TEM due to the particular lipid organization [55, 64, 65]. A few additional proteins, where pronounced coimmunoprecipitation with CD44v6 suggested facilitating signaling pathway activation, are MOV10 (Putative helicase MOV-10) engaged in EPH-Ephrin and Wnt signaling [66] as well as MYO1D (Unconventional myosin-Id) and SPTBN1 (Spectrin beta chain, nonerythrocytic 1), which both are involved in GPCR activation [67, 68]. Taken together, the association of CD44v6 with signal transducing molecules could significantly assist CIC-TEX-promoted modulation of non-CIC. We want to stress three features of selective CD44v6 coimmunoprecipitating molecules. (i) The TEM microenvironment and the TEM transfer into TEX strongly expand the range of selectively CD44v6-associated signal transducing molecules. (ii) A strong preponderance of GPCR and EPH as well as of junction-engaged signaling molecules should be taken into account considering a blockade of TEX-CD44v6 as a therapeutic option. (iii) As demonstrated in Figures 5(b) and 5(c), most of the CD44v6-associated molecules are components of signaling pathways. With all caution, we suggest that this is an important notice, which might indicate that functional consequences are becoming likely by altered expression of network-integrated rather than single molecules.

A last aspect of the coimmunoprecipitation analysis, the striking differences between wt and CD44v6kd cells and TEX in the association with proteins engaged in RNA processing require further attention. The findings are in line with the reported CD44v6 association with Dicer [68] and the linkage of CD44v6 to mRNA processing proteins. The predominant recovery in TEX fits to CD44 supporting ILV loading and the cargo being transferred via MVB into TEX [69, 70]. The analysis of CD44v6 versus CD44 coimmunoprecipitating proteins allowed for the first time unraveling the selective contribution of CD44v6. We are confident that the data provide a solid ground uncovering the features predestinating CD44v6 for the cooperation with the RNA processing machinery. Reaching beyond our question on the contribution of the CIC marker CD44v6 in PaCa progression and its relevance for TEX-mediated message transfer, progress in enlightening the components of the RNA processing machinery and their mode of interaction will facilitate achieving an answer.

The proteome analysis of proteins coimmunoprecipitating with CD44v6 unraveled a wide spectrum of protein families that share a possible contribution to PaCa progression. This includes proteins engaged in vesicle formation and transport, the majority being also linked to Tspan8, signal transduction molecules, some of which (integrins) becoming activated and recruited into TEM and associate with Tspan8, while the association with others, demonstrated for Tspan8 and NOTCH1, initiates transcription. Furthermore, CD44v6 is liaised with RNA processing proteins. Finally, as CD44v6 is engaged in Tspan8 transcription and is associated with Tspan8 in TEM, which is important in EE formation and transport, the majority of deficits noted in CD44v6kd cells are also seen in CD44v6kd-TEX. Few exceptions likely rely on non-TEM-derived TEX, the biogenesis of which following distinct routes [50].

### 4.5. CD44v6, miRNA Processing, and the Contribution of Tspan8 to TEX-Loading

TEX miRNA may have a share in target reprogramming [2, 6]. Previous work [32] and the reduced recovery of some proteins in CD44v6 immunoprecipitates suggested a selective contribution of CD44v6 to miRNA processing, which we controlled for miRNA described to be engaged in PaCa growth and progression

The PaCa lines A818.4 and Capan1 as well as the colon cancer line SW480 (unpublished) and TEX derived thereof display similar miRNA profiles, which correspond to published profiles [71], confirming the reliability of the DS analysis. Instead, the recovery of some miRNA differed between cells and TEX [32, 72], indicating a nonrandom recruitment. Searching for a suggested contribution of CD44v6 to miRNA processing revealed reduced recovery of 28 miRNA in CD44v6kd cells, only 7 miRNA being also reduced in Tspan8kd but none only in Tspan8kd cells. The impact of CD44v6 on miRNA recovery [32] is in line with the CD44v6-Dicer and -Argonaute (Ago) associations [73, 74]. Furthermore, reduced miRNA expression in CD44v6kd, but not Tspan8kd cells, argues against Tspan8 engagement in miRNA processing. Distinct to cells, a high number of miRNA were reduced in CD44v6kd and Tspan8kd TEX. Reduced miRNA recovery in Tspan8kd TEX likely relies on the engagement of Tspan8 in vesicle traffic [52], the concomitantly reduced recovery in CD44v6kd TEX being supported by the association of CD44v6 with Tspan8. Reduced recovery of few miRNA selectively in CD44v6kd TEX may be a sequel of the described contribution of CD44 in recruiting the mRNA processing machinery into TEX [75]. The latter implies miRNA processing within TEX. However, this was not observed in our tumor model (unpublished).

CIC-TEX promoting a shift in the mass of nonmetastatic tumor cells towards tumor dissemination, we exclusively searched for predicted mRNA of miRNA engaged in tumor cell-ECM interactions, cancer-related proteoglycans, and stem cell-/cancer cell-related signaling. First to note, miRNA with an impact on PaCa are more abundant in TEX than cells, and the vast majority of miRNA higher in A818.4-wt than -CD44v6kd cells or TEX is engaged in at least one of these processes. In view of the abundance of predicted target mRNA, we only listed predicted signaling-engaged targets for miRNA reduced in CD44v6kd cells and their engagement in 33 signaling pathways, most mRNA being predicted targets of more than one miRNA and most mRNA being engaged in several pathways. Though this accounts for miRNA higher in wt than CD44v6kd cells and TEX, the numbers of predicted signaling- and signaling pathways-engaged targets as well as the connectivity of several predicted targets with a range of signaling pathways is higher in TEX than cells. It is also interesting that predicted targets of miRNA with reduced expression in CD44v6kd cells confirm the particular engagement of CD44v6 in proteoglycan but also in stem cell maintenance and signaling pathways in cancer. Finally, it is worthwhile commending on predicted targets of some miRNA recovered at a reduced level. Downregulation of Smad4 (SMAD family member 4), c-Myb (MYB protooncogene), Muc4 (cell surface glycoprotein MUC4), or Deptor (DEP domain containing MTOR interacting protein) would hamper invasion and apoptosis resistance [76–79].

The results of these screenings require approval. As a first step, we controlled for predicted target cell recovery of signaling-engaged miRNA enriched in wt compared to CD44v6kd cells. From the 123 predicted targets, 47 mRNA were recovered at a higher level in CD44v6kd cells. Expression of the majority of predicted mRNA was unchanged and, opposing expectation, 15 mRNA were recovered at a lower level in CD44v6kd cells. Though awaiting confirmation by qRT-PCR and functional studies, the present state of knowledge does not allow a solid interpretation. One could speculate that tumor lines are not well suited by missing the stress situation. However, TEX were collected from cells cultured in the absence of FCS, which mimics a stress situation. Nonetheless, mRNA and miRNA recovery in TEX mostly corresponded to the recovery in cells. We favor as a possible explanation that pathway deviations and activation of feedback loops are of particular concern in the regulation of signaling cascades and miRNA. One of the miRNA regulators are lncRNA, some of which can sponge miRNA.

The multifaceted roles of lncRNA only recently received intense attention [80–83]. Though many questions remain to be answered, (competing endogenous) lncRNA can contribute to mRNA liberation by sponging miRNA, but lncRNA also can contain sequences that act as miRNA [84]. We listed those lncRNA in which expression differs between wt and CD44v6kd cells or between wt TEX and cells. This list may become a guide for detailed analyses after further progress on functional relevance of noncoding RNA [83, 84]. Nonetheless, the more frequently observed reduced lncRNA recovery in CD44v6kd cells might be in favor of reduced miRNA levels still affecting mRNA recovery, whereas high level lncRNA expression in CD44v6kd cells could account for unaltered mRNA recovery by sponging miRNA. Both features may in part explain the apparently random correlation between miRNA and mRNA expression in CD44v6kd cells. A precise answer requires further studies on the regulation of miRNA.

Despite remaining questions, CD44v6 affects miRNA processing in cells and may contribute to miRNA processing in TEX. A minor contribution of Tspan8 relies on the efficient ILV cargo transfer into TEX. Though the majority of CD44v6-regulated miRNA have predicted mRNA targets engaged in tumor progression, the mRNA analyses pointed towards a contribution by additional regulators, one of which could be lncRNA, links between CD44 and lncRNA in gastrointestinal cancer being described [85–88]. However, in view of the current state of knowledge on the interplay between CD44 and the components of the RNA processing machinery and the even less progressed state on the multiple functions of lncRNA, an interpretation of these last screenings would be premature but may help guiding step by step towards a profound elucidation.

## 5. Conclusion and Outlook

A CD44v6kd and a Tspan8kd are accompanied by a loss in metastatic capacity of PaCa [10, 21], which prompted us speculating that these CIC markers jointly influence crucial CIC activities. We show that CD44v6 is directly engaged in Tspan8 transcription, associates with RTK, EMT transcription factors, and molecules important for mRNA and miRNA processing. Tspan8 is an essential contributor to TEX biogenesis and binding. Thereby, defects associated with a CD44v6kd are transferred into TEX. This implies a central role of CD44v6 in shaping CIC and, beyond our hypothesis, via Tspan8 in TEX-mediated message transfer.

Meanwhile we made some progress related to the functional impact of TEX CD44v6 and Tspan8 on CD44v6kd tumor progression that confirmed a central role of Tspan8 in non-CIC targeting and an efficient CD44v6-dependent reprogramming of the TEX target, TEX acting as a hub in target cell-autonomous program activation [89]. The confirmation at the functional level of the utmost importance of TEX CD44v6 is a driving stimulus for answering the remaining questions. Besides elaborating the role of tetraspanins/Tspan8 in routing EE towards MVB and the suggested deviation from degradation in the proteasome, the central importance of CD44v6 in furnishing TEX to cover multiple aspects of tumor progression demands clarification. This includes (i) unraveling the contribution of CD44 to transcription, which is independent of the cotranscription factor activity of the CD44ICD, (ii) the mode of interaction with the mRNA translation machinery allowing for selected miRNA recruitment into ILV, and, (iii) last but not least, which TEX components recruited via CD44v6 account for target reprogramming. Attacking these tasks is crucial in view of the efficient prevention of tumor progression by a CD44v6kd. The answers may also shed light on the mode of the TEX crosstalk with nontransformed targets, expecting an adjuvant therapy interfering with TEX CD44v6 expression well exceeding the power of a therapeutic blockade of individual RTK or miRNA. A concomitant blockade of Tspan8 may potently hamper CIC message transfer. Thus, the two main messages are the efficacy of the CIC-biomarker CD44v6 in driving tumor progression at several levels and the urgent need for further studies on regulatory circuit.

## Data Availability

DS analyses are deposited at ENA database (accession no. PRJEB25446); proteome analysis is available at Functional Proteome Analysis (files: ZW2612, ZW2484, and SH2726), German Cancer Research Center, Heidelberg.

## Abbreviations

Ago: Argonaute
Akt: Rac-alpha serine/threonine-protein kinase AKT
Alix: PDCD6IP, programmed cell death 6 interacting protein
*β*-cat: *β*-catenin
CD44s: CD44 standard isoform
CD44v6: CD44 variant 6 isoform
ceRNA: Competing endogenous RNA
CIC: Cancer initiating cells
CLTC: Clathrin heavy chain 1
c-Myb: MYB protooncogene
CSPG4: Chondroitin sulfate proteoglycan 4
DEAD: DEAD-box helicase
Deptor: DEP domain containing MTOR interacting protein
Dicer: Endoribonuclease Dicer
Dio/SP-DioC_18_(3): 3,3′-dioctadecyl-5,5′-di(4-sulfophenyl)oxacarbocyanine
DS: Deep sequencing
ECL: Enhanced chemiluminescence
EMT: Epithelial-mesenchymal transition
EPH: Ephrin receptor
Ephrin: Ephrin
Exo: Exosomes
FPRP: Prostaglandin F2 receptor negative regulator
GLI1: GLI family zinc finger 1
GPCR: G protein-coupled receptor
ICD: Intracellular domain
IP: Immunoprecipitation
ILV: Intraluminal vesicle
IPA: Ingenuity program analysis
kd: Knockdown
Lamp1: Lysosomal associated membrane protein 1
LB: Latex beads
LGR5: Leucine rich repeat containing G protein-coupled receptor 5
lncRNA: Long noncoding RNA
LRP6: LDL receptor related protein 6
MAPK: Mitogen activated protein kinase
MDR1: Multidrug resistance 1
MFGE8: Lactadherin
MOV10: Putative helicase MOV-10
MUC4: Cell surface glycoprotein MUC4
MVB: Multivesicular bodies
MYO1D: Unconventional myosin-Id
MYH3: Myosin-3
PaCa: Pancreatic cancer
PI3K: Phosphoinositide-3-kinase
SC: Stem cells
qRT-PCR: Quantitative real-time polymerase chain reaction
RISC: RNA-induced silencing complex
RQ: Relative quantification
RTK: Receptor tyrosine kinase
SMAD4: SMAD family member 4
SPTBN1: Spectrin beta chain, nonerythrocytic 1
TEM: Tetraspanin-enriched membrane domains
TEX: Tumor/CIC exosomes
TSG101: Tumor susceptibility gene 101 protein
WB: Western blot
wt: Wild type
YBX3: Y-box-binding protein 3.

## Supplementary Materials

Supplementary Material The supplementary information contains 2 tables for Methods and 5 tables and 5 figures for Results in PDF format. Table S1: primers. Table S2: antibodies. Table S3: CD44v6-associated proteins in A818.4 cells and TEX. Table S4: abundant miRNA in cells and TEX: comparison between A818.4 and Capan1. Table S5: the impact of CD44v6 and Tspan8 on miRNA recovery. Table S6: full names of protein and mRNA synonyms. Table S7: full names of lncRNA and information on function. Figure S1: classification of CD44- and CD44v6-associated molecules in cells and TEX. Figure S2: miRNA profile of PaCa cells and TEX. Figure S3: predicted targets of miRNA higher in A818.4 wt than CD44v6kd cells or TEX. Figure S4: impact of CD44v6 and Tspan8 on the mRNA profile in cells and TEX. Figure S5: distinct lncRNA recovery.

## References

[1] Ajani J. A., Song S., Hochster H. S., Steinberg I. B. (2015). Cancer stem cells: the promise and the potential. *Seminars in Oncology*.

[2] Greening D. W., Gopal S. K., Mathias R. A. (2015). Emerging roles of exosomes during epithelial–mesenchymal transition and cancer progression. *Seminars in Cell & Developmental Biology*.

[3] Hannafon B. N., Ding W. Q. (2015). Cancer stem cells and exosome signaling. *Stem Cell Investig*.

[4] Zöller M. (2016). Exosomes in Cancer Disease. *Methods in Molecular Biology*.

[5] Kumar D., Gupta D., Shankar S., Srivastava R. K. (2015). Biomolecular characterization of exosomes released from cancer stem cells: possible implications for biomarker and treatment of cancer. *Oncotarget*.

[6] Wang H., Rana S., Giese N., Büchler M. W., Zöller M. (2013). Tspan8, CD44v6 and alpha6beta4 are biomarkers of migrating pancreatic cancer-initiating cells. *International Journal of Cancer*.

[7] Giovannetti E., van der Borden C., Frampton A., Ali A., Firuzi O., Peters G. (2017). Never let it go: Stopping key mechanisms underlying metastasis to fight pancreatic cancer. *Seminars in Cancer Biology*.

[8] Zöller M. (2011). CD44: can a cancer-initiating cell profit from an abundantly expressed molecule?. *Nature Reviews Cancer*.

[9] Andreu Z., Yáñez-Mó M. (2014). Tetraspanins in extracellular vesicle formation and function. *Frontiers in Immunology*.

[10] Wang Z., von Au A., Schnölzer M., Hackert T., Zöller M. (2016). CD44v6-competent tumor exosomes promote motility, invasion and cancer-initiating cell marker expression in pancreatic and colorectal cancer cells. *Oncotarget*.

[11] Morath I., Jung C., Lévêque R. (2018). Differential recruitment of CD44 isoforms by ErbB ligands reveals an involvement of CD44 in breast cancer. *Oncogene*.

[12] Schmitt M., Metzger M., Gradl D., Davidson G., Orian-Rousseau V. (2015). CD44 functions in Wnt signaling by regulating LRP6 localization and activation. *Cell Death & Differentiation*.

[13] Natsuizaka M., Whelan K. A., Kagawa S. (2017). Interplay between Notch1 and Notch3 promotes EMT and tumor initiation in squamous cell carcinoma. *Nature Communications*.

[14] Cho Y., Lee H., Kang H., Kim H., Kim S., Chun K. (2015). Cleaved CD44 intracellular domain supports activation of stemness factors and promotes tumorigenesis of breast cancer. *Oncotarget*.

[15] Safa A. R. (2016). Resistance to cell death and its modulation in cancer stem cells. *Critical Reviews in Oncogenesis*.

[16] Misra S., Ghatak S., Toole B. P. (2005). Regulation of MDR1 expression and drug resistance by a positive feedback loop involving hyaluronan, phosphoinositide 3-kinase, and ErbB2. *The Journal of Biological Chemistry*.

[17] Ferrer V. P., Moura Neto V., Mentlein R. (2018). Glioma infiltration and extracellular matrix: key players and modulators. *Glia*.

[18] Lin L., Jiang H., Huang M., Hou X., Sun X., Jiang X. (2015). Depletion of histone deacetylase 1 inhibits metastatic abilities of gastric cancer cells by regulating the miR-34a/CD44 pathway. *Oncology Reports*.

[19] Chen L., Bourguignon L. Y. (2014). Hyaluronan-CD44 interaction promotes c-Jun signaling and miRNA21 expression leading to Bcl-2 expression and chemoresistance in breast cancer cells. *Molecular Cancer*.

[20] Fang T., Lin J., Wang Y. (2016). Tetraspanin-8 promotes hepatocellular carcinoma metastasis by increasing ADAM12m expression. *Oncotarget*.

[21] Yue S., Mu W., Zöller M. (2013). Tspan8 and CD151 promote metastasis by distinct mechanisms. *European Journal of Cancer*.

[22] Zöller M. (2009). Tetraspanins: push and pull in suppressing and promoting metastasis. *Nature Reviews Cancer*.

[23] Tauro B. J., Greening D. W., Mathias R. A. (2012). Comparison of ultracentrifugation, density gradient separation, and immunoaffinity capture methods for isolating human colon cancer cell line LIM1863-derived exosomes. *Methods*.

[24] Van Deun J., Mestdagh P., Agostinis P., Akay Ö., Anand S., Anckaert J. (2017). EV-TRACK: transparent reporting and centralizing knowledge in extracellular vesicle research. *Nature Methods*.

[25] Xu R., Simpson R. J., Greening D. W. (2016). A protocol for isolation and proteomic characterization of distinct extracellular vesicle subtypes by sequential centrifugal ultrafiltration. *Methods in Molecular Biology*.

[26] Guo B. B., Bellingham S. A., Hill A. F. (2015). The Neutral Sphingomyelinase Pathway Regulates Packaging of the Prion Protein into Exosomes. *The Journal of Biological Chemistry*.

[27] Janas T., Janas M. M., Sapoń K., Janas T. (2015). Mechanisms of RNA loading into exosomes. *FEBS Letters*.

[28] Villarroya-Beltri C., Baixauli F., Gutiérrez-Vázquez C., Sánchez-Madrid F., Mittelbrunn M. (2014). Sorting it out: regulation of exosome loading. *Seminars in Cancer Biology*.

[29] Rana S., Yue S., Stadel D., Zöller M. (2012). Toward tailored exosomes: the exosomal tetraspanin web contributes to target cell selection. *The International Journal of Biochemistry & Cell Biology*.

[30] Li J., Bonifati S., Hristov G. (2013). Synergistic combination of valproic acid and oncolytic parvovirus H-1PV as a potential therapy against cervical and pancreatic carcinomas. *EMBO Molecular Medicine*.

[31] Vlachos I. S., Zagganas K., Paraskevopoulou M. D. (2015). DIANA-miRPath v3.0: deciphering microRNA function with experimental support. *Nucleic Acids Research*.

[32] Rana S., Malinowska K., Zöller M. (2013). Exosomal tumor microRNA modulates premetastatic organ cells. *Neoplasia*.

[33] Wu K., Xing F., Wu S., Watabe K. (2017). Extracellular vesicles as emerging targets in cancer: Recent development from bench to bedside. *Biochimica et Biophysica Acta (BBA) - Reviews on Cancer*.

[34] Wang Z., Zhao K., Hackert T., Zöller M. (2018). CD44/CD44v6 a reliable companion in cancer-initiating cell maintenance and tumor progression. *Frontiers in Cell and Developmental Biology*.

[35] Claas C., Wahl J., Orlicky D. J., Karaduman H., Schnölzer M., Kempf T. (2005). The tetraspanin D6.1A and its molecular partners on rat carcinoma cells. *Biochemical Journal*.

[36] Zeilstra J., Joosten S. P. J., van Andel H. (2014). Stem cell CD44v isoforms promote intestinal cancer formation in Apc(min) mice downstream of Wnt signaling. *Oncogene*.

[37] Apostolou P., Toloudi M., Ioannou E. (2013). Study of the interaction among Notch pathway receptors, correlation with stemness, as well as their interaction with CD44, dipeptidyl peptidase-IV, hepatocyte growth factor receptor and the SETMAR transferase, in colon cancer stem cells. *Journal of Receptors and Signal Transduction*.

[38] Ibrahim S. A., Gadalla R., El-Ghonaimy E. A. (2017). Syndecan-1 is a novel molecular marker for triple negative inflammatory breast cancer and modulates the cancer stem cell phenotype via the IL-6/STAT3, Notch and EGFR signaling pathways. *Molecular Cancer*.

[39] Senbanjo L. T., Chellaiah M. A. (2017). CD44: A multifunctional cell surface adhesion receptor is a regulator of progression and metastasis of cancer cells. *Frontiers in Cell and Developmental Biology*.

[40] Mukohyama J., Shimono Y., Minami H., Kakeji Y., Suzuki A. (2017). Roles of microRNAs and RNA-binding proteins in the regulation of colorectal cancer stem cells. *Cancers*.

[41] Saitoh M. (2018). Involvement of partial EMT in cancer progression. *The Journal of Biochemistry*.

[42] Zheng H., Kang Y. (2014). Multilayer control of the EMT master regulators. *Oncogene*.

[43] Simeone P., Trerotola M., Franck J., Cardon T., Marchisio M., Fournier I. (2018). The multiverse nature of epithelial to mesenchymal transition. *Seminars in Cancer Biology*.

[44] Kuhn S., Koch M., Nübel T. (2007). A complex of EpCAM, claudin-7, CD44 variant isoforms, and tetraspanins promotes colorectal cancer progression. *Molecular Cancer Research*.

[45] Heiler S., Wang Z., Zöller M. (2016). Pancreatic cancer stem cell markers and exosomes - the incentive push. *World Journal of Gastroenterology*.

[46] Rana S., Claas C., Kretz C. C., Nazarenko I., Zoeller M. (2011). Activation-induced internalization differs for the tetraspanins CD9 and Tspan8: impact on tumor cell motility. *The International Journal of Biochemistry & Cell Biology*.

[47] Viswanathan K., Verweij M. C., John N., Malouli D., Früh K. (2017). Quantitative membrane proteomics reveals a role for tetraspanin enriched microdomains during entry of human cytomegalovirus. *PLoS ONE*.

[48] van Deventer S. J., Dunlock V. E., van Spriel A. B. (2017). Molecular interactions shaping the tetraspanin web. *Biochemical Society Transactions*.

[49] Berditchevski F. (2001). Complexes of tetraspanins with integrins: more than meets the eye. *Journal of Cell Science*.

[50] Van Niel G., D'Angelo G., Raposo G. (2018). Shedding light on the cell biology of extracellular vesicles. *Nature Reviews Molecular Cell Biology*.

[51] Stuffers S., Sem Wegner C., Stenmark H., Brech A. (2009). Multivesicular endosome biogenesis in the absence of ESCRTs. *Traffic*.

[52] Edgar J. R., Eden E. R., Futter C. E. (2014). Hrs- and CD63-dependent competing mechanisms make different sized endosomal intraluminal vesicles. *Traffic*.

[53] Canfrán-Duque A., Pastor Ó., Quintana-Portillo R. (2014). Curcumin promotes exosomes/microvesicles secretion that attenuates lysosomal cholesterol traffic impairment. *Molecular Nutrition & Food Research*.

[54] Legate K. R., Wickström S. A., Fässler R. (2009). Genetic and cell biological analysis of integrin outside-in signaling. *Genes & Development*.

[55] Termini C. M., Gillette J. M. (2017). Tetraspanins function as regulators of cellular signaling. *Frontiers in Cell and Developmental Biology*.

[56] Yu J., Lee C., Changou C. A., Cedano-Prieto D. M., Takada Y. K., Takada Y. (2017). The CD9, CD81, and CD151 EC2 domains bind to the classical RGD-binding site of integrin *α*v*β*3. *Biochemical Journal*.

[57] Shimizu H., Shimoda M., Mochizuki S. (2018). Hyaluronan-binding protein involved in hyaluronan depolymerization is up-regulated and involved in hyaluronan degradation in human osteoarthritic cartilage. *The American Journal of Pathology*.

[58] Iida J., Dorchak J., Clancy R. (2015). Role for chondroitin sulfate glycosaminoglycan in NEDD9-mediated breast cancer cell growth. *Experimental Cell Research*.

[59] Li Y. Y., Zhou C. X., Gao Y. (2014). Snail regulates the motility of oral cancer cells via RhoA/Cdc42/p-ERM pathway. *Biochemical and Biophysical Research Communications*.

[60] Nagase H., Nakayama K. (2013). *γ*-Secretase-regulated signaling typified by Notch signaling in the immune system. *Current Stem Cell Research & Therapy*.

[61] Zhang C., Mao H.-L., Cao Y. (2017). Nuclear accumulation of symplekin promotes cellular proliferation and dedifferentiation in an ERK1/2-dependent manner. *Scientific Reports*.

[62] Wang Y., Yago T., Zhang N. (2014). Cytoskeletal Regulation of CD44 Membrane Organization and Interactions with E-selectin. *The Journal of Biological Chemistry*.

[63] Park J., Han Y., Jeong M. (2017). Synthetic 8-hydroxydeoxyguanosine inhibited metastasis of pancreatic cancer through concerted inhibitions of ERM and Rho-GTPase. *Free Radical Biology & Medicine*.

[64] Yunta M., Lazo P. A. (2003). Tetraspanin proteins as organisers of membrane microdomains and signalling complexes. *Cellular Signalling*.

[65] Florin L., Lang T. (2018). Tetraspanin assemblies in virus infection. *Frontiers in Immunology*.

[66] Wang W., Snyder N., Worth A. J., Blair I. A., Witze E. S. (2015). Regulation of lipid synthesis by the RNA helicase Mov10 controls Wnt5a production. *Oncogenesis*.

[67] Amcheslavsky A., Wang S., Fogarty C. E., Lindblad J. L., Fan Y., Bergmann A. (2018). Plasma membrane localization of apoptotic caspases for non-apoptotic functions. *Developmental Cell*.

[68] Li N., Hu P., Xu T. (2017). iTRAQ-based proteomic analysis of APPSw,Ind Mice provides insights into the early changes in alzheimer's disease. *Current Alzheimer Research*.

[69] Ameyar-Zazoua M., Rachez C., Souidi M. (2012). Argonaute proteins couple chromatin silencing to alternative splicing. *Nature Structural & Molecular Biology*.

[70] Geis-Asteggiante L., Belew A. T., Clements V. K. (2018). Differential content of proteins, mRNAs, and miRNAs suggests that MDSC and their exosomes may mediate distinct immune suppressive functions. *Journal of Proteome Research*.

[71] Melo S. A., Sugimoto H., O’Connell J. T. (2014). Cancer exosomes perform cell-independent microRNA biogenesis and promote tumorigenesis. *Cancer Cell*.

[72] Madhavan B., Yue S., Galli U. (2015). Combined evaluation of a panel of protein and miRNA serum-exosome biomarkers for pancreatic cancer diagnosis increases sensitivity and specificity. *International Journal of Cancer*.

[73] Subramani R., Gangwani L., Nandy SB., Arumugam A., Chattopadhyay M., Lakshmanaswamy R. (2015). Emerging roles of microRNAs in pancreatic cancer diagnosis, therapy and prognosis. *International Journal of Oncology*.

[74] Shah K., Patel S., Modi B., Shah F., Rawal R. (2018). Uncovering the potential of CD44v/SYNE1/miR34a axis in salivary fluids of oral cancer patients. *Journal of Oral Pathology & Medicine*.

[75] Harel-Bellan A., Ameyar-Zazoua M., Rachez C., Muchardt C., Batsché E. (2014). 10-million-years AGO: argonaute on chromatin in yeast and human, a conserved mode of action?. *Transcription*.

[76] Kuo W. T., Yu S. Y., Li S. C., Lam H. C., Chang H. T., Chen W. S. (2016). MicroRNA-324 in human cancer: miR-324-5p and miR-324-3p have distinct biological functions in human cancer. *Anticancer Research*.

[77] Yang K., He M., Cai Z. (2015). A decrease in miR-150 regulates the malignancy of pancreatic cancer by targeting c-Myb and MUC4. *Pancreas*.

[78] Quwaider D., Corchete L. A., Misiewicz-Krzeminska I. (2017). DEPTOR maintains plasma cell differentiation and favorably affects prognosis in multiple myeloma. *Journal of Hematology & Oncology*.

[79] Cheng D., Zhao S., Tang H. (2016). MicroRNA-20a-5p promotes colorectal cancer invasion and metastasis by downregulating Smad4. *Oncotarget*.

[80] Jarroux J., Morillon A., Pinskaya M. (2017). History, discovery, and classification of lncRNAs. *Advances in Experimental Medicine and Biology*.

[81] Quinn J. J., Chang H. Y. (2015). Unique features of long non-coding RNA biogenesis and function. *Nature Reviews Genetics*.

[82] Bhan A., Soleimani M., Mandal S. S. (2017). Long noncoding RNA and cancer: a new paradigm. *Cancer Research*.

[83] Kovalenko T. F., Patrushev L. I. (2018). Pseudogenes as functionally significant elements of the genome. *Biochemistry (Moscow)*.

[84] Grüll M. P., Massé E. (2019). Mimicry, deception and competition: the life of competing endogenous RNAs. *Wiley Interdisciplinary Reviews: RNA*.

[85] Hu Y., Wang J., Qian J. (2014). Long noncoding RNA GAPLINC regulates CD44-dependent cell invasiveness and associates with poor prognosis of gastric cancer. *Cancer Research*.

[86] Wu X., He X., Li S., Xu X., Chen X., Zhu H. (2016). Long non-coding RNA ucoo2kmd.1 regulates CD44-dependent cell growth by competing for miR-211-3p in colorectal cancer. *PLoS ONE*.

[87] Di Cecilia S., Zhang F., Sancho A. (2016). RBM5-AS1 is critical for self-renewal of colon cancer stem-like cells. *Cancer Research*.

[88] Yu X., Mi L., Dong J., Zou J. (2017). Long intergenic non-protein-coding RNA 1567 (LINC01567) acts as a ‘sponge’ against microRNA-93 in regulating the proliferation and tumorigenesis of human colon cancer stem cells. *BMC Cancer*.

[89] Wang Z., Sun H., Provaznik J., Hackert T., Zöller M. (2019). Pancreatic cancer-initiating cell exosome message transfer into noncancer-initiating cells: the importance of CD44v6 in reprogramming. *Journal of Experimental & Clinical Cancer Research*.

