# Supplementary material for: The Pancreatic Cancer-Initiating Cell Marker CD44v6 Affects Transcription, Translation, and Signaling: Consequences for Exosome Composition and Delivery
Source: J Oncol. 2019 Aug 7;2019:3516973. doi: 10.1155/2019/3516973 (PMC6702834; doi:10.1155/2019/3516973)
Supplement: Supplementary Material — The supplementary information contains 2 tables for Methods and 5 tables and 5 figures for Results in PDF format. Table S1: primers. Table S2: antibodies. Table S3: CD44v6-associated proteins in A818.4 cells and TEX. Table S4: abundant miRNA in cells and TEX: comparison between A818.4 and Capan1. Table S5: the impact of CD44v6 and Tspan8 on miRNA recovery. Table S6: full names of protein and mRNA synonyms. Table S7: full names of lncRNA and information on function. Figure S1: classification of CD44- and CD44v6-associated molecules in cells and TEX. Figure S2: miRNA profile of PaCa cells and TEX. Figure S3: predicted targets of miRNA higher in A818.4 wt than CD44v6kd cells or TEX. Figure S4: impact of CD44v6 and Tspan8 on the mRNA profile in cells and TEX. Figure S5: distinct lncRNA recovery. [file 3516973.f1.pdf]

Supplementary information

The pancreatic cancer-initiating cell marker CD44v6 affects transcription, translation and signaling: consequences for exosome composition and delivery

Hanxue Sun[a], Sanyukta Rana[a], Zhe Wang[a], Kun Zhao[a], Martina Schnölzer[b], Jan Provaznik[c], Thilo Hackert[d], Qingjie Lv[e], Margot Zöller[a]
[a]Tumor Cell Biology, University Hospital of Surgery, Heidelberg, [b]Functional proteome analysis, German Cancer Research Center, Heidelberg, [c]Gene Core Unit, EMBL Heidelberg, [d]Section Pancreas Research, University Hospital of Surgery, Heidelberg, Germany, [e]Department of Pathology, Shengjing Hospital of China Medical University, Shenyang, China

Table S1	Primers
Table S2	Antibodies
Table S3	CD44v6 associated proteins in A818.4 cells and TEX
Table S4	Abundant miRNA in cells and TEX: Comparison between A818.4 and Capan1
Table S5	The impact of CD44v6 and Tspan8 on miRNA recovery
Table S6	Full names of protein and mRNA synonyms
Table S7	Full names of lncRNA and information on function
Figure S1	Classification of CD44- and CD44v6-associated molecules in cells and TEX
Figure S2	miRNA profile of PaCa cells and TEX
Figure S3	Predicted targets of miRNA higher in A818.4 wt than CD44v6kd cells or TEX
Figure S4	Impact of CD44v6 and Tspan8 on the mRNA profile in cells and TEX
Figure S5	Distinct lncRNA recovery

TableS1

Primers

CD44v6kd primers
CD44v6 fw: ACCGGTGTAGTACAACCTGCGGAAGAACGAATTCTTCCGCAGGTTGTACTAC
CD44v6 rev: GAATTCGTAGTACAACCTGCGGAAGAATTCGTTCTTCCGCAGGTTGTACTAC

Tspan8kd primers
Tspan8 fw: 5'CCGGGCAATATGGGTACGAGTAAGCCTCGAGGCTTACTCGTACCCATATTGCTTTTTG 3'
Tspan8 rev: 5' AATTCAAAAAGCAATATGGGTACGAGTAAGCCTCGAGGCTTACTCGTACCCATATTGC 3'

CD44ICD primers
CD44ICD fw: 5'-5'-GTT TCTAGAATGAGTCGAAGAAGGTGTGGG-3''
CD44ICD rev: 5' 5'-GTT GGATCCTTACACCCCAATCTTCATGTC-3''

qRT-PCR mRNA primers

GAPDH fw	aggtcggagtcaacggattt
GAPDH rev	atctcgctcctggaagatgg
CD44ICD fw	gtttctagaatgagtcgaagaaggtgtggg
CD44ICD rev	gttggatccttacaccccaatcttcatgtc
Tspan8 fw	gcttgcttctgatcctgctc
Tspan8 rev	attgaccaaaccgcagcatt
CXCR4 fw	ctggccttcatcagtctgga
CXCR4 rev	tcatctgcctcactgacgtt
CD104 fw	ggataaggactgcgcctact
CD104 rev	tggtgtcaatctgggtctcc
CD151 fw	gcagcaacaactcacaggac
CD151 rev	atcatgccaaagacctgcac
Nanog fw	cagccaaattctcctgccag
Nanog rev	tcacacgtcttcaggttgca
Notch-1 fw	gtcaacgccgtagatgacc
Notch-1 rev	ttgttagccccgttcttcag
Notch-2 fw	tccacttcatactcacagtta
Notch-2 rev	tggttcagagaaaacataca
MET fw	ccaaccgagacaagcatc
MET rev	gttgagaggttctttccacca

qRT-PCR miRNA primers

let-7d-5p SL	GTTGGCTCTGGTGCAGGGTCCGAGGTATTCGCACCAGAGCCAACAACTAT
let-7d-5p fw	GGCGAGAGGTAGTAGGTTGCAT
let-7e-5p SL	GTTGGCTCTGGTGCAGGGTCCGAGGTATTCGCACCAGAGCCAACAACTAT
let-7e-5p fw	GCGTGAGGTAGGAGGTTGTAT
miR-10b-5p SL	GTTGGCTCTGGTGCAGGGTCCGAGGTATTCGCACCAGAGCCAACCCACAAA
miR-10b-5p fw	GGCGTACCCTGTAGAACCGAA
miR-103a-5p SL	GTTGGCTCTGGTGCAGGGTCCGAGGTATTCGCACCAGCCAACCAAGGC
miR-103a-5p fw	CAGGCTTCTTTACAGTGCTGC
miR-125a-5p SL	GTTGGCTCTGGTGCAATACCTCGGACCCTGCACCCAGAGCCAACTCACAG
miR-125a-5p fw	GGCTCCCTGAGACCCTTTAAC
miR-196a-5p SL	GTTGGCTCTGGTGCAATACCTCGGACCCTGCACCAGAGCCAACCCCAAC
miR-196a-5p fw	GGGCGTAGGTAGTTTCATGTTGT
miR-1246 SL	GTTGGCTCTGGTGCAGGTCCAGGTATTCGCACCAGAGCCAACCCTGCT
miR-1246 fw	GGGCTAATGGATTTTTGGAGC
miR-1290 SL	GTTGGCTCTGGTGCAGGGTCCGAGGTATTCGCACCAGAGCCAACATCCTG
miR-1290 fw	GGTGGATTTTTGGATCAGG
miR-4284 SL	GTTGGCTCTGGTGCAGGTCCAGGTATTCGCACAGAGCCAACTATGGGG
miR-4284 fw	CCTATTCCCAGCCAACGCACCA
miR-6089 SL	GTTGGCTCTGGTGCAGGGTCCGAGGTATTCGCACCAGAGCCAACCCCGCC
miR-6089 fw	TATTGGAGGCCGGGGTGG
miR-7975 SL	GTTGGCTCTGGTGCAGGTCCAGGTATTCGCACAGAGCCAACTGGTGC
miR-7975 fw	GGATCCTAGTCACGGCACCA
miR-7977 SL	GTTGGCTCTGGTGCAGGTCCAGGTATTCGCACAGAGCCAACTGGTTC
miR-7977 fw	CCTATTCCCAGCCAACGCACCA

TableS2

Antibodies

Antibody	origin	supplier
Actin	mouse	Becton Dickinson, HD, G
Alix	rabbit	Santa Cruz, HD, G
β-catenin	rabbit	Becton Dickinson, HD, G
CD9	mouse	ImmunoTools, Friesoythe, G
CD44 (HERMES3)	mouse	ref [1]
CD44ICD	rabbit	Cosmo Bio, Japan
CD44v6 (vFF18)	mouse	ref [2]
CD49f	mouse	Becton Dickinson, HD, G
CD63	mouse	Becton Dickinson, HD, G
CD81	mouse	Becton Dickinson, HD, G
CD104	rabbit	Becton Dickinson, HD, G
CD107a	mouse	Becton Dickinson, HD, G
CD151 (11B1)	mouse	ref [3]
CD184 (CXCR4)	mouse	Becton Dickinson, HD, G
E-cadherin	mouse	Becton Dickinson, HD, G
EpCAM (HEA125)	mouse	ref [4]
EphA4	rabbit	Santa Cruz, HD, G
Ezrin	rabbit	Sigma, Munich, G
FN	mouse	Becton Dickinson, HD, G
FPRB	rabbit	Abcam, Cambridge, England
Frizzled	rabbit	Abcam, Cambridge, England
Histone 3	rabbit	Abcam, Cambridge, England
LGR5	rabbit	Biolzol, Eching, Germany
MDR1	mouse	Becton Dickinson, HD, G
MET	mouse	Cell Signaling, Frankfurt, G
MFGE8	mouse	MERCK, Darmstadt, G
Nanog	rabbit	Santa Cruz, HD, G
N-Cadherin	mouse	Becton Dickinson, HD, G
NOTCH	mouse	Biolegend, San Diego, Ca, US
Rab7	rabbit	Santa Cruz, HD, G
Slug	rabbit	Santa Cruz, HD, G
Snail	rabbit	Santa Cruz, HD, G
Sox2	rabbit	Santa Cruz, HD, G
TSG101	rabbit	SantaCruz, HD, G
Tspan8 (CO029)	mouse	ref [5]
Twist	rabbit	Becton Dickinson, HD, G
vimentin	mouse	Becton Dickinson, HD, G
Wnt 1	rabbit	Santa Cruz, HD, G
Wnt5a	rabbit	Santa Cruz, HD, G
ZEB1	rabbit	Santa Cruz, Heidelberg, G
dye or biotin labeled secondary antibodies / Streptavidin	Dianova, Becton Dickinson, Amersham	

References

1. Picker LJ, De los Toyos J, Telen MJ, Haynes BF, Butcher EC. Monoclonal antibodies against the CD44 [In(Lu)-related p80], and Pgp-1 antigens in man recognize the Hermes class of lymphocyte homing receptors. J Immunol. 1989;142:2046-51.
2. Seiter S, Tilgen W, Herrmann K, Schadendorf D, Patzelt E, Möller P, Zöller M. Expression of CD44 splice variants in human skin and epidermal tumours. Virchows Arch. 1996;428:141-9.
3. Geary SM, Cambareri AC, Sincock PM, Fitter S, Ashman LK. Differential tissue expression of epitopes of the tetraspanin CD151 recognised by monoclonal antibodies. Tissue Antigens. 2001;58:141-53.
4. Momburg F, Moldenhauer G, Hämmerling GJ, Möller P. Immunohistochemical study of the expression of a Mr 34,000 human epithelium-specific surface glycoprotein in normal and malignant tissues. Cancer Res. 1987;47: 2883-91.
5. Sela BA, Steplewski Z, Koprowski H. Colon carcinoma-associated glycoproteins recognized by monoclonal antibodies CO-029 and GA22-2. Hybridoma. 1989;8:481-91.

Table S3

CD44v6 associated proteins in A818.4 cells and TEX

CD44v6-linked in cells and TEX

	**cells**				**TEX**				
**Synonym**	**A818.4**		**A818.4-v6kd**		**A818.4**		**A818.4-v6kd**		**full name**
	**prot.M.**	**pept.M**	**prot.M.**	**pept.M**	**prot.M.**	**pept.M**	**prot.M.**	**pept.M**	
ABCC3	20	9			69	20			Canalicular multispecific organic anion transporter 2
ACADVL	8	5			2	2			Very long-chain specific acyl-CoA dehydrogenase
AGR2	2	2			4	2			Anterior gradient protein 2 homolog
ARPC3	6	4	1	1	6	4			Actin-related protein 2/3 complex subunit 3
ARPC4	5	3			7	4			Actin-related protein 2/3 complex subunit 4
ATP1A1	101	23			176	34	6	3	Sodium/potassium-transporting ATPase subunit alpha-1
ATP1B1	10	2			23	6			Sodium/potassium-transporting ATPase subunit beta-1
CALX	7	4			6	3			Calnexin
CD44	149	10	9	3	167	12	8	3	CD44 antigen
CIRBP	19	9			3	3			Calcium homeostasis endoplasmic reticulum protein
CLD3	6	3			6	3			Claudin-3
COPA	16	10			5	4			Coatomer subunit alpha
CSNK2A1	3	2			5	4			Casein kinase II subunit alpha
CSPG4	80	39			167	50			Chondroitin sulfate proteoglycan 4
EFTU	4	2			25	16	6	4	Elongation factor Tu
EIF5A	21	7			10	3	3	2	Eukaryotic translation initiation factor 5A-1
FLNB	26	18	3	2	65	36	7	5	Filamin-B
FLNC	2	2			6	4			Filamin-C
FPRP	7	4			4	2			Prostaglandin F2 receptor negative regulator
GNAI2	5	3			14	8			Guanine nucleotide-binding protein G(i) subunit alpha-2
GSTP1	4	2			19	8			Glutathione S-transferase P
HADHA	2	2			5	3			Trifunctional enzyme subunit alpha
HMGCS2	7	5			25	10			Hydroxymethylglutaryl-CoA synthase
ITGA2	5	3			17	9			Integrin alpha-2
ITGA6	16	7			41	19			Integrin alpha-6
ITGAV	13	9	2	1	21	11			Integrin alpha-V
ITGB1	3	2			25	11			Integrin beta-1
ITGB4	15	9			28	16			Integrin beta-4
MUC18	7	5			18	10			Cell surface glycoprotein MUC18
MYH14	108	49			48	23			Myosin-14
MYO1C	83	37	6	5	39	22			Unconventional myosin-Ic
MYO1D	29	19	2	2	6	5			Unconventional myosin-Id
PDCD6	17	6	2	1	5	4	1	1	Programmed cell death protein 6
PGAM5	8	5			7	3			Serine/threonine-protein phosphatase PGAM5
PPIB	8	4			29	10	4	2	Peptidyl-prolyl cis-trans isomerase B
PRKDC	61	41	4	3	81	46	31	22	DNA-dependent protein kinase catalytic subunit
RBBP8NL	6	4			11	5			RBBP8 N-terminal-like protein
SLC12A2	47	12			76	18			Solute carrier family 12 member 2
SLC1A5	26	15	1	1	5	3			4F2 cell-surface antigen heavy chain
SLC2A1	31	6	5	1	9	4			Solute carrier family 2, facilitated glucose transporter memb
SPTAN1	76	43	3	2	62	37	19	12	Spectrin alpha chain, non-erythrocytic 1
SPTBN1	56	32	2	2	59	35	22	16	Spectrin beta chain, non-erythrocytic 1
ST14	3	2			8	5			Suppressor of tumorigenicity 14 protein
STX4	3	2			3	2			Syntaxin-4
TFRC	10	5			36	19	2	1	Transferrin receptor protein 1
TOP1	68	20	1	1	4	2			DNA topoisomerase 1
TRAP1	6	5			11	4			Heat shock protein 75 kDa
TSPAN8	2	2			4	2			Tetraspanin-8
VILI	8	3			4	2			Villin-1

Table S3 cont.

CD44v6-linked only in cells

	cells				TEX				
**Synonym**	**A818.4**		**A818.4-v6kd**		**A818.4**		**A818.4-v6kd**		**full name**
	**prot.M.**	**pept.M**	**prot.M.**	**pept.M**	**prot.M.**	**pept.M**	**prot.M.**	**pept.M**	
ATP2A2	10	5							Sarcoplasmic/endoplasmic reticulum calcium ATPase 2
BAG2	4	2							BAG family molecular chaperone regulator 2
BCAP31	5	3							B-cell receptor-associated protein 31
BRIX1	5	2							Ribosome biogenesis protein BRX1 homolog
C14orf166	5	4							UPF0568 protein C14orf166
CKAP4	5	3							Cytoskeleton-associated protein 4
CLTC	33	20	11	8	6	5	15	10	Clathrin heavy chain 1
CTCF	7	3							Transcriptional repressor CTCF
DBN1	7	5							Drebrin
DDOST	8	5							Dolichyl-diphosphooligosacch.protein glycosyltransf. 48kDa
DDX1	40	20			5	4	7	4	ATP-dependent RNA helicase DDX1
DDX21	94	26			3	2			Nucleolar RNA helicase 2
DDX23	35	17			9	6	3	2	Probable ATP-dependent RNA helicase DDX23
DDX3X	41	22	5	4	12	9	11	6	ATP-dependent RNA helicase DDX3X
DDX5	46	21	1	1	7	4	11	6	Probable ATP-dependent RNA helicase DDX5
DDX50	17	7							ATP-dependent RNA helicase DDX50
DDX54	18	11							ATP-dependent RNA helicase DDX54
DHX15	40	21							Pre-mRNA-splicing factor ATP-dependent RNA helicase DHX
DHX30	27	13							Putative ATP-dependent RNA helicase DHX30
DHX9	99	31			7	4	4	4	ATP-dependent RNA helicase A
DICER1	3	2							Endoribonuclease Dicer
DUSP12	5	3							Dual specificity protein phosphatase 12
EEF2	42	17	3	2	15	9	7	5	Elongation factor 2
EIF2AK2	3	2							Interferon-induced, double-stranded RNA-activated protein kir
ELAV1	7	5							ELAV-like protein 1
ETFA	2	2							Electron transfer flavoprotein subunit alpha, mitochondrial
FBRL	4	2							rRNA 2'-O-methyltransferase fibrillarin
FIP1L1	6	3							Pre-mRNA 3'-end-processing factor FIP1
FLII	3	2							Protein flightless-1 homolog
FMR1	12	7							Fragile X mental retardation protein 1
FXR1	17	10	1	1					Fragile X mental retardation syndrome-related protein 1
GNL3	9	5							Guanine nucleotide-binding protein-like 3
GTPBP4	28	14							Nucleolar GTP-binding protein 1
HSD17B2	10	6							Estradiol 17-beta-dehydrogenase 2
IGF2BP2	28	13							Insulin-like growth factor 2 mRNA-binding protein 2
IGF2BP3	42	17							Insulin-like growth factor 2 mRNA-binding protein 3
ILF2	15	7			6	4	7	4	Interleukin enhancer-binding factor 2
ILF3	45	20	1	1	9	5	11	8	Interleukin enhancer-binding factor 3
ITPR3	4	3							Inositol 1,4,5-trisphosphate receptor type 3
KI67	37	20							Antigen KI-67
KIAA1429	43	26							Protein virilizer homolog
LA	21	11	2	1	4	3	3	2	Lupus La protein =SSB
LARP1	17	8							La-related protein 1
LARP7	29	16							La-related protein 7
LC7L2	22	9							Putative RNA-binding protein Luc7-like 2
LLPH	4	2							Protein LLP homolog
LMAN2	5	3							Vesicular integral-membrane protein VIP36
LMO7	12	6	1	1					LIM domain only protein 7
LUC7L	7	3							Putative RNA-binding protein Luc7-like 1
LYAR	8	4							Cell growth-regulating nucleolar protein
MATR3	16	9			6	4	2	2	Matrin-3
MLEC	12	7							Malectin
MMTA2	5	3							Multiple myeloma tumor-associated protein 2
MOV10	27	15							Putative helicase MOV-10
MRTO4	37	15							mRNA turnover protein 4 homolog
MY18A	34	19							Unconventional myosin-XVIIIa
MYH3	8	2							Myosin-3
MYO6	7	5	2	2					Unconventional myosin-VI

Table S3 cont.

CD44v6-linked only in cells

	cells				TEX				
**Synonym**	**A818.4**		**A818.4-v6kd**		**A818.4**		**A818.4-v6kd**		**full name**
	**prot.M.**	**pept.M**	**prot.M.**	**pept.M**	**prot.M.**	**pept.M**	**prot.M.**	**pept.M**	
NCBP1	**3**	**2**							Nuclear cap-binding protein subunit 1
NDKA	**8**	**5**	**1**	**1**					Nucleoside diphosphate kinase A
NIPS1	**4**	**2**							Protein NipSnap homolog 1
NKRF	**10**	**6**							NF-kappa-B-repressing factor
NUCL	**113**	**23**	**14**	**8**					Nucleolin
PA2G4	**7**	**5**							Proliferation-associated protein 2G4
PABPC1	**39**	**16**	**2**	**1**					Polyadenylate-binding protein 1
PABPC4	**26**	**14**							Polyadenylate-binding protein 4
PCBP1	**11**	**5**			4	3	8	4	Poly(rC)-binding protein 1
PCBP2	**6**	**3**			3	2	4	2	Poly(rC)-binding protein 2
PHB2	**9**	**6**							Prohibitin-2
PLOD3	**4**	**3**			2	2	2	2	Procollagen-lysine,2-oxoglutarate 5-dioxygenase 3
POP1	**22**	**12**							Ribonucleases P/MRP protein subunit POP1
PPIA	**12**	**6**	**3**	**2**	35	9	21	7	Peptidyl-prolyl cis-trans isomerase A
PPP1CA	**8**	**5**	**2**	**1**	2	2	3	3	Serine/threonine-protein phosphatase PP1-alpha catalytic sub
PRKRA	**8**	**6**							Interferon-inducible double-stranded RNA-dep. kinase activato
PRPF8	**34**	**24**	**3**	**1**	22	14			Pre-mRNA-processing-splicing factor 8
PTCD3	**6**	**3**							Pentatricopeptide repeat domain-containing protein 3
PUF60	**20**	**10**	**4**	**2**					Poly(U)-binding-splicing factor PUF60
PURA	**8**	**4**							Transcriptional activator protein Pur-alpha
RACK1	**71**	**18**							Receptor of activated protein C kinase 1
RALY	**6**	**3**							RNA-binding protein Raly
RBM10	**8**	**5**							RNA-binding protein 10
RBM14	**4**	**3**			1	1	14	7	RNA-binding protein 14
RBM15	**10**	**6**							Putative RNA-binding protein 15
RBM39	**29**	**13**							RNA-binding protein 39
RBMX	**24**	**8**							RNA-binding motif protein, X chromosome
RPN1	**44**	**21**							Dolichyl-diphosphooligosacch.protein glycosyltransf.subunit 1
RPN2	**19**	**10**			3	2			Dolichyl-diphosphooligosacch. protein glycosyltransf.subunit 2
SAFB1	**18**	**6**	**1**	**1**					Scaffold attachment factor B1
SCAF1	**4**	**3**							Splicing factor, arginine/serine-rich 19
SEC11A	**7**	**5**							Signal peptidase complex catalytic subunit SEC11A
SEC61A1	**4**	**2**							Protein transport protein Sec61 subunit alpha isoform 1
SERBP1	**8**	**6**							Plasminogen activator inhibitor 1 RNA-binding protein
SF3B1	**6**	**4**			2	1	1	1	Splicing factor 3B subunit 1
SKIV2L2	**4**	**2**							Superkiller viralicidic activity 2-like 2
SMARCA5	**2**	**2**							Matrix-assoc.actin-dependent regul. of chromatin subfamily A5
SPATS2L	**9**	**5**							SPATS2-like protein
SPCS2	**6**	**3**							Signal peptidase complex subunit 2
SPCS3	**3**	**2**							Signal peptidase complex subunit 3
SPF45	**5**	**3**							Splicing factor 45
SRP19	**3**	**2**							Signal recognition particle 19 kDa protein
SRPK2	**3**	**2**							SRSF protein kinase 2
SRSF9	**8**	**6**							Serine/arginine-rich splicing factor 9
SSR1	**5**	**3**							Translocon-associated protein subunit alpha
SSR4	**10**	**3**							Translocon-associated protein subunit delta4
SSRP1	**10**	**6**	**1**	**1**					FACT complex subunit SSRP1
STAU1	**5**	**3**							Double-stranded RNA-binding protein Staufen homolog 1
STRBP	**12**	**7**							Spermatid perinuclear RNA-binding protein
STT3A	**5**	**3**							Dolichyl-diphosphooligosacch. glycosyltransf.subunit STT3A
SUB1	**7**	**4**	**1**	**1**					Activated RNA polymerase II transcriptional coactivator p15
SUPT16H	**13**	**7**							FACT complex subunit SPT16
TFB1M	**3**	**2**							Dimethyladenosine transferase 1
TMED10	**25**	**7**							Transmembrane emp24 domain-containing protein 10
TMED2	**2**	**2**							Transmembrane emp24 domain-containing protein 2
TMED4	**4**	**2**							Transmembrane emp24 domain-containing protein 4
TMED9	**6**	**3**							Transmembrane emp24 domain-containing protein 9
TMX1	**3**	**2**							Thioredoxin-related transmembrane protein 1

Table S3 cont.

CD44v6-linked only in cells

Synonym	cells				TEX				full name
	A818.4		A818.4-v6kd		A818.4		A818.4-v6kd		
	prot.M.	pept.M	prot.M.	pept.M	prot.M.	pept.M	prot.M.	pept.M	
TOP2B	30	16			4	2			DNA topoisomerase 2-beta
TSR1	19	11							Pre-rRNA-processing protein TSR1 homolog
U2AF1	14	8	6	3	3	2	2	1	Splicing factor U2AF 35 kDa subunit
VAPA	6	3							Vesicle-associated membrane protein-associated protein A
WTAP	3	2							Pre-mRNA-splicing regulator WTAP
XRCC5	5	3			8	5	9	8	X-ray repair cross-complementing protein 5
XRCC6	4	3			2	1	1	1	X-ray repair cross-complementing protein 6
XRN2	8	6							5'-3' exoribonuclease 2
YBX1	21	7	1	1	4	3	5	3	Nuclease-sensitive element-binding protein 1
YBX3	16	5							Y-box-binding protein 3
YLPM1	21	12	1	1			3	2	YLP motif-containing protein 1
YTHDC2	29	19							Probable ATP-dependent RNA helicase YTHDC2
ZC3H13	22	15							Zinc finger CCCH domain-containing protein 13
ZNF326	15	8							DBIRD complex subunit ZNF326

Table S3 cont.

CD44v6-linked only in TEX

	cells				TEX				
**Synonym**	**A818.4**		**A818.4-v6kd**		**A818.4**		**A818.4-v6kd**		**full name**
	**prot.M.**	**pept.M**	**prot.M.**	**pept.M**	**prot.M.**	**pept.M**	**prot.M.**	**pept.M**	
ABCC1					21	8			Multidrug resistance-associated protein 1
ACAT1					10	6	2	1	Acetyl-CoA acetyltransferase
AHNAK					21	11	7	3	Neuroblast differentiation-associated protein AHNAK
AKR1B10					8	4			Aldo-keto reductase family 1B10
ALDH18A1					3	3			Delta-1-pyrroline-5-carboxylate synthase
ANXA1					10	6			Annexin A1
ATP1A2					66	11			Sodium/potassium-transporting ATPase subunit alpha-2
ATP1B3					9	4			Sodium/potassium-transporting ATPase subunit beta-3
ATP2B1					20	7			Plasma membrane calcium-transporting ATPase 1
CALML5					17	7	1	1	Calmodulin-like protein 5
CD63					3	2			CD63 antigen
CD81					8	3			CD81 antigen
CD9					5	3	2	1	CD9 antigen
CKB					14	7			Creatine kinase B-type
CTSD					4	3			Cathepsin D
DNAJC10					11	7	3	2	DnaJ homolog subfamily C member 10
EFNB1					9	4			Ephrin-B1
EPHB4					4	2			Ephrin type-B receptor 4
EPPK1					12	9			Epiplakin
EZR					8	5	1	1	Ezrin
FABP5					21	8	2	1	Fatty acid-binding protein
FASN					20	12	6	5	Fatty acid synthase
FLOT2					13	8			Flotillin-2
GANAB					6	4	1	1	Neutral alpha-glucosidase AB
GCN1					11	7			eIF-2-alpha kinase activator GCN1
GNA13					9	5			Guanine nucleotide-binding protein subunit alpha-13
GNAI1					11	6			Guanine nucleotide-binding protein G(i) subunit alpha-1
GNAI3					12	7			Guanine nucleotide-binding protein G(k) subunit alpha
GNAS1					7	3			Guanine nucleotide-binding protein G(s) subunit alpha XLas
GNB1					2	2			Guanine nucleotide-binding protein G(I)/G(S)/G(T) subunit bet
GOLGA7					3	3			Golgin subfamily A member 7
GOT2					14	9	7	4	Aspartate aminotransferase, mitochondrial
HADHB					23	13	7	5	Trifunctional enzyme subunit beta
HLA-B15a					8	4			HLA class I histocompatibility antigen, B-15 alpha chain
HLA-B44a					7	4			HLA class I histocompatibility antigen, B-44 alpha chain
HSPB1					15	8	2	1	Heat shock protein beta-1
IDH2					17	9			Isocitrate dehydrogenase
ITGB5					5	3			Integrin beta-5
JAM1					6	3			Junctional adhesion molecule A
JUP					18	11			Junction plakoglobin
LGALS7					27	7			Galectin-7
MYH10	91	41	97	33	24	12			Myosin-10
MYH9	285	83	499	106	128	48	17	12	Myosin-9
PARP1	5	4	3	2	14	8			Poly [ADP-ribose] polymerase 1
PDHB					8	5			Pyruvate dehydrogenase E1 component subunit beta
PDIA1					18	10	7	5	Protein disulfide-isomerase
PDIA3					24	14	4	3	Protein disulfide-isomerase A3
PEBP1					4	3			Phosphatidylethanolamine-binding protein 1
PLXB2					4	3			Plexin-B2
POF1B					7	6			Protein POF1B
POTEI					42	7			POTE ankyrin domain family member I
PRDX5					9	4			Peroxiredoxin-5
PTBP1					6	4	1	1	26S proteasome non-ATPase regulatory subunit 11
PTPRF					9	5			Protein tyrosine phosphatase, receptor type F
PYGB					9	6			Glycogen phosphorylase B
RAB7A					6	5	1	1	RAB7A, member RAS oncogene family
RAP1B					7	5			RAP1B, member RAS oncogene family
RAP2B					4	3			RAP2B, member RAS oncogene family

Table S3 cont.

CD44v6-linked only in TEX

	cells				TEX				
**Synonym**	**A818.4**		**A818.4-v6kd**		**A818.4**		**A818.4-v6kd**		**full name**
	**prot.M.**	**pept.M**	**prot.M.**	**pept.M**	**prot.M.**	**pept.M**	**prot.M.**	**pept.M**	
RAP2C					5	3			RAP2C, member RAS oncogene family
RUVBL1					5	3			RuvB-like 1
S100A7					13	7	1	1	S100 calcium binding protein A7
S100A8					27	7	3	1	S100 calcium binding protein A8
S100A9					39	10	1	1	S100 calcium binding protein A9
SDHA					6	4	1	1	Succinate dehydrogenase [ubiquinone] flavoprotein subunit
SEC23B					4	4	1	1	Protein transport protein Sec23B
SERPINB12					5	4	2	1	Serpin B12
SERPINB3					69	24			Serpin B3
SERPINB4					68	23			Serpin B4
SET					7	4			Protein SET
SHMT2	3	2	4	3	12	7	2	2	Serine hydroxymethyltransferase
SLC3A2					44	18	6	4	solute carrier family 3 member 2
SLC7A5					7	3			solute carrier family 7 member 5
SND1					12	8	2	2	staphylococcal nuclease and tudor domain containing 1
SOD1					21	13	2	2	Superoxide dismutase
SPRR2F					7	3			Spectrin alpha chain, non-erythrocytic 1
TAF15	11	5	8	2	4	2			TATA-binding protein-associated factor 2N
TALDO1					7	5			Transaldolase
TGM3					5	4			Protein-glutamine gamma-glutamyltransferase E
TUBA1B					28	10			Tubulin alpha-1B chain
TUBA4A					13	8			Tubulin alpha-4A chain
U2AF2	33	9	11	6	4	3			Splicing factor U2AF 65 kDa subunit
UBA1					6	5			Ubiquitin-like modifier-activating enzyme 1
YWHAE	8	2	12	6	30	15	9	4	14-3-3 protein epsilon

Table S4

Abundant miRNA in cells and TEX: Comparison between A818.4 and Capan1

	signal strength cells				signal strength TEX	
**Name**	**A818.4**	**Capan1**		**Name**	**A818.4**	**Capan1**
**let-7a-5p**	17143	19617		**let-7a-5p**	29811	27188
**let-7b-5p**	10796	11191		**let-7b-5p**	17099	15273
**let-7c-5p**	2168	2237		**let-7c-5p**	2964	2876
**let-7d-5p**	5054	6660		**let-7d-5p**	10268	9698
**let-7e-5p**	1041	2301		**let-7e-5p**	9113	8945
**let-7f-5p**	14288	14871		**let-7f-5p**	25360	22762
**let-7g-5p**	5197	3974		**let-7g-5p**	8467	8642
**let-7i-5p**	3090	9883		**let-7i-5p**	8343	6622
**miR-103a-3p**	6406	6156		**miR-103a-3p**	8478	8968
**miR-106b-5p**	6657	4233		**miR-106b-5p**	14130	15538
**miR-107**	4109	3688		**miR-107**	7043	7238
**miR-10a-5p**	7820	2025		**miR-10a-5p**	12693	14924
**miR-1202**	2158	2376		**miR-1202**	3211	2641
**miR-1273g-3p**	5964	2797		**miR-1273g-3p**	3588	2290
**miR-141-3p**	11417	12318		**miR-141-3p**	17720	17364
**miR-15a-5p**	4013	2751		**miR-15a-5p**	3054	3114
**miR-15b-5p**	12314	10420		**miR-15b-5p**	10164	10091
**miR-16-5p**	23265	17242		**miR-16-5p**	16405	15662
**miR-17-5p**	6196	12822		**miR-17-5p**	7371	8546
**miR-19a-3p**	4383	8749		**miR-19a-3p**	4230	4685
**miR-19b-3p**	12282	20510		**miR-19b-3p**	10941	12913
**miR-200a-3p**	2795	1786		**miR-200a-3p**	8550	7877
**miR-200b-3p**	17234	9274		**miR-200b-3p**	16703	15660
**miR-200c-3p**	3569	5254		**miR-200c-3p**	8567	7898
**miR-20a-5p**	12911	23123		**miR-20a-5p**	18978	21370
**miR-20b-5p**	1853	3580		**miR-20b-5p**	2920	3384
**miR-21-5p**	43576	61528		**miR-21-5p**	68591	53546
**miR-22-3p**	1408	2592		**miR-22-3p**	4163	2861
**miR-23a-3p**	2552	2398		**miR-23a-3p**	10758	8701
**miR-23b-3p**	3405	527		**miR-23b-3p**	3136	2899
**miR-24-3p**	11391	6233		**miR-24-3p**	6193	5801
**miR-25-3p**	5742	3777		**miR-25-3p**	7805	8384
**miR-26a-5p**	2061	698		**miR-26a-5p**	3055	3570
**miR-26b-5p**	1082	551		**miR-26b-5p**	6160	6124
**miR-27a-3p**	5757	3861		**miR-27a-3p**	5494	5250
**miR-27b-3p**	11187	1266		**miR-27b-3p**	2950	2883
**miR-29a-3p**	34620	16463		**miR-29a-3p**	14113	15822
**miR-29b-3p**	20203	9577		**miR-29b-3p**	9648	9405
**miR-29c-3p**	2207	1934		**miR-29c-3p**	4232	4388
**miR-30b-5p**	3592	1790		**miR-30b-5p**	5821	6283
**miR-3162-5p**	3469	3586		**miR-3162-5p**	4043	3821
**miR-3960**	4943	1290		**miR-3960**	13841	16202
**miR-4281**	1021	404		**miR-4281**	5197	3994
**miR-4286**	5106	2277		**miR-4286**	6647	11086
**miR-429**	5083	3157		**miR-429**	5931	5838
**miR-4459**	3554	1582		**miR-4459**	16438	23230
**miR-4516**	4187	1103		**miR-4516**	12953	16268
**miR-4530**	2366	991		**miR-4530**	12265	18126
**miR-494-3p**	1964	1620		**miR-494-3p**	11645	12039
**miR-5100**	21591	28999		**miR-5100**	13014	15228
**miR-5739**	5765	7559		**miR-5739**	3673	4630
**miR-6087**	5880	2656		**miR-6087**	20534	22052
**miR-6089**	10263	4505		**miR-6089**	26631	25045
**miR-6090**	2357	734		**miR-6090**	10799	7038
**miR-6125**	1540	816		**miR-6125**	7639	8179
**miR-642a-3p**	1127	780		**miR-642a-3p**	8756	6914
**miR-6749-5p**	3461	4353		**miR-6749-5p**	3562	4024

Table S4 cont.

	signal strength cells			signal strength TEX	
Name	A818.4	Capan1	Name	A818.4	Capan1
**miR-6821-5p**	1159	397	**miR-6821-5p**	3912	4144
**miR-6869-5p**	3671	1601	**miR-6869-5p**	13042	13026
**miR-7-5p**	1245	3218	**miR-7-5p**	5863	5796
**miR-7641**	3676	4382	**miR-7641**	10037	9525
**miR-7975**	364157	398862	**miR-7975**	90693	85788
**miR-7977**	333533	333094	**miR-7977**	68987	72024
**miR-8069**	5871	4578	**miR-8069**	19023	14389
miR-1225-5p	1385	489	miR-1246	59235	54464
miR-1260a	6342	8925	miR-1268a	5693	14944
miR-1260b	1442	10472	miR-1287-5p	5980	3164
miR-130b-3p	1190	728	miR-1290	20828	22217
miR-135b-5p	2381	824	miR-151a-5p	3790	4088
miR-150-5p	1382	112	miR-185-5p	3596	3187
miR-1915-3p	1021	404	miR-188-5p	2909	2473
miR-196b-5p	1100	1574	miR-192-5p	21112	20888
miR-320b	1039	831	miR-194-5p	9526	10848
miR-320d	1013	764	miR-196a-5p	3451	3974
miR-324-3p	2242	2119	miR-215-5p	11754	11406
miR-34a-5p	1297	121	miR-31-5p	2953	3176
miR-3665	1137	410	miR-320c	2894	3113
miR-3934-5p	2162	455	miR-4306	6255	4770
miR-424-5p	2358	179	miR-4505	3848	3014
miR-4284	14126	13698	miR-451a	6086	3503
miR-499a-5p	3987	167	miR-5703	3149	4527
miR-6085	7137	9029	miR-574-5p	4835	8388
miR-6127	1516	1305	miR-5787	5748	9558
miR-6165	2354	3035	miR-6088	4253	4157
miR-6826-5p	2926	4761	miR-630	4105	6578
miR-6875-5p	2077	1499	miR-6800-5p	3002	3165
miR-8089	2955	602	miR-6879-5p	3092	1738
miR-92a-3p	1025	2046	miR-7107-5p	11224	2355
miR-93-5p	4072	3085	miR-7150	7103	6958
miR-96-5p	5303	1772	miR-7704	4326	6021

[a] abundant in cells and TEX**: bold**

Table S5

The impact of CD44v6 and Tspan8 on miRNA recovery

Table S5A

Reduced miRNA recovery in CD44v6kd and Tspan8kd cells

	rel. intensity			ratio	
Name	A818.4 cells	-v6kd cells	-Tsp8kd cells	wt:v6kd cells	wt:Tsp8kd cells
miR-17-5p	11889	7577	7046	1.57	1.69
miR-18a-5p	2360	786	801	3.00	2.95
miR-196a-5p	7517	2352	2120	3.20	3.55
miR-19a-3p	8079	5097	3680	1.59	2.20
miR-221-3p	1345	615	725	2.19	1.85
miR-31-3p	3456	214	1427	16.17	2.42
miR-455-3p	1069	400	467	2.67	2.29
let-7d-5p	8688	3620	5993	2.40	
let-7e-5p	6409	3179	6501	2.02	
let-7i-5p	7365	2932	6718	2.51	
miR-125a-5p	1435	419	1180	3.42	
miR-1260b	6493	1400	5086	4.64	
miR-151a-3p	481	172	405	2.80	
miR-151a-5p	1680	662	1794	2.54	
miR-185-5p	2803	1074	2842	2.61	
miR-192-5p	8649	4690	7250	1.84	
miR-194-5p	3613	1965	4294	1.84	
miR-22-3p	2714	1352	2841	2.01	
miR-31-5p	2178	1332	2340	1.64	
miR-324-5p	1075	491	990	2.19	
miR-331-3p	1422	958	1315	1.48	
miR-374a-5p	1580	864	1671	1.83	
miR-374b-5p	1240	428	1317	2.90	
miR-4306	1606	678	1537	2.37	
miR-590-5p	1187	592	1262	2.00	
miR-7-5p	3212	1584	2821	2.03	
miR-98-5p	1029	664	1013	1.55	
miR-99b-5p	858	480	729	1.79	

Table S5B

Reduced miRNA recovery in CD44v6kd and Tspan8kd TEX

Name	rel intensity A818.4 TEX	-v6kd TEX	-Tsp8kd TEX	ratio wt:v6kd TEX	wt:Tsp8kd TEX
let-7a-5p	29811	11927	9409	2.499	3.168
let-7b-5p	17099	9539	5560	1.792	3.076
let-7c-5p	2964	1611	889	1.840	3.332
let-7d-5p	10268	2738	2632	3.750	3.902
let-7e-5p	9113	1137	2843	8.018	3.205
let-7f-5p	25360	9262	7802	2.738	3.251
let-7g-5p	8467	3923	2142	2.158	3.952
let-7i-5p	8343	1638	5445	5.093	1.532
miR-101-3p	1330	363	354	3.666	3.753
miR-103a-3p	8478	3301	2584	2.569	3.281
miR-106b-5p	14130	6935	4449	2.038	3.176
miR-107	7043	2555	1770	2.756	3.978
miR-10a-5p	12693	6658	3745	1.906	3.389
miR-10b-5p	1499	490	396	3.062	3.788
miR-125a-5p	1849	360	663	5.144	2.790
miR-130b-3p	1508	752	369	2.004	4.083
miR-141-3p	17720	9050	8284	1.958	2.139
miR-148a-3p	1233	799	790	1.543	1.560
miR-151a-3p	1208	100	332	12.078	3.636
miR-151a-5p	3790	1668	1335	2.272	2.839
miR-151b	2786	1170	577	2.381	4.832
miR-17-5p	7371	2701	2192	2.729	3.363
miR-181a-5p	1626	318	572	5.112	2.842
miR-185-5p	3596	402	1224	8.946	2.938
miR-188-5p	2909	1094	1243	2.659	2.341
miR-192-5p	21112	1166	7487	18.106	2.820
miR-194-5p	9526	4012	4204	2.374	2.266
miR-196a-5p	3451	442	597	7.807	5.776
miR-196b-5p	1375	465	301	2.959	4.573
miR-19a-3p	4230	1131	992	3.739	4.265
miR-19b-3p	10941	4246	3032	2.577	3.609
miR-200a-3p	8550	5226	3690	1.636	2.317
miR-200c-3p	8567	4008	3244	2.138	2.641
miR-20a-5p	18978	7986	5683	2.376	3.339
miR-20b-5p	2920	1162	808	2.514	3.615
miR-210-3p	2231	1126	908	1.981	2.457
miR-215-5p	11754	3720	2810	3.160	4.184
miR-21-5p	68591	33932	40223	2.021	1.705
miR-22-3p	3512	1653	1512	2.125	2.323
miR-23a-3p	10758	7108	3285	1.514	3.275
miR-24-3p	6193	3642	1880	1.701	3.295
miR-25-3p	7805	2830	2055	2.758	3.799
miR-26b-5p	6160	3059	1956	2.014	3.150
miR-27a-3p	5494	3621	2238	1.517	2.455
miR-29c-3p	4232	1069	1623	3.958	2.607
miR-30b-5p	5821	2902	2007	2.006	2.901
miR-30c-5p	1507	728	502	2.069	3.003
miR-30d-5p	1743	800	691	2.179	2.522
miR-30e-5p	1597	638	532	2.502	3.004
miR-31-3p	1842	342	472	5.385	3.905

Table S5B cont.

Name	rel. intensity A818.4 TEX	-v6kd TEX	-Tsp8kd TEX	ratio wt:v6kd TEX	wt:Tsp8kd TEX
miR-31-5p	2953	723	816	4.084	3.619
miR-331-3p	1472	221	607	6.660	2.426
miR-34a-5p	1858	1069	794	1.739	2.340
miR-374a-5p	2487	786	863	3.164	2.880
miR-374b-5p	1661	385	558	4.313	2.976
miR-375	1429	239	201	5.967	7.103
miR-425-5p	1449	782	577	1.853	2.511
miR-429	5931	2813	1736	2.109	3.418
miR-4306	6255	1646	2061	3.800	3.035
miR-494-3p	11645	4396	2024	2.649	5.753
miR-5703	3149	713	1512	4.418	2.083
miR-590-5p	1902	301	421	6.315	4.517
miR-630	4105	924	1340	4.444	3.063
miR-6769b	1651	809	871	2.041	1.897
miR-6875-5p	1208	454	360	2.662	3.355
miR-7150	7031	3482	2630	2.019	2.674
miR-7-5p	5863	1176	2342	4.985	2.504
miR-7641	10037	4491	2585	2.235	3.882
miR-92a-3p	2531	1128	630	2.243	4.017
miR-93-5p	2430	727	391	3.343	6.210
miR-96-5p	2639	906	664	2.913	3.976
miR-98-5p	1147	204	250	5.622	4.592
miR-99b-5p	1312	217	492	6.045	2.666
miR-1287-5p	5980	2755	4762	2.171	
miR-4455	1029	384	857	2.679	
miR-574-5p	4835	1702	3860	2.841	
miR-7107-5p	11224	1415	9737	7.932	
miR-135b-5p	1066	1853	389		2.738
miR-15a-5p	3054	2432	1013		3.016
miR-15b-5p	10164	7214	3369		3.017
miR-16-5p	16405	14956	6705		2.447
miR-183-5p	1169	1080	315		3.712
miR-200b-3p	16703	16245	5269		3.170
miR-23b-3p	3017	2516	1093		2.760
miR-26a-5p	3055	2316	1174		2.603
miR-27b-3p	2950	3041	1031		2.860
miR-29a-3p	14113	10113	4410		3.200
miR-29b-3p	9648	7119	3710		2.601
miR-320c	2894	2032	974		2.971
miR-320e	1231	1406	399		3.088
miR-4299	1269	1008	519		2.445
miR-4721	1349	2002	674		2.003
miR-642a-3p	8756	8777	3504		2.499

Table S6
Full names of synonyms (alphabetic)

Synonym	Full name
A2M	Alpha-2-macroglobulin
ABCC1	Multidrug resistance-associated protein 1
ABCC3	Canalicular multispecific organic anion transporter 2
ABL1	Tyrosine-protein kinase ABL1
ACTB	Actin, cytoplasmic 1
ACTC1	Actin, alpha cardiac muscle 1
ACTN4	Alpha-actinin-4
ADCY1	Adenylate cyclase type 1
ADCY6	Adenylate cyclase type 6
ADCY9	Adenylate cyclase type 9
AKT1	Rac-alpha serine/threonine-protein kinase AKT1
AKT2	Rac-alpha serine/threonine-protein kinase AKT2
AKT3	Rac-alpha serine/threonine-protein kinase AKT3
ALB	Albumin
ANXA1	Annexin A1
APAF1	Apoptotic protease-activating factor 1
APC	Adenomatous polyposis coli protein
APH1A	Gamma-secretase subunit APH-1A
ARF1	ADP-ribosylation factor 1
ARHGEF1	Rho guanine nucleotide exchange factor 1
ARHGEF10	Rho guanine nucleotide exchange factor 10
ARHGEF11	Rho guanine nucleotide exchange factor 11
ARHGEF12	Rho guanine nucleotide exchange factor 12
ARHGEF15	Rho guanine nucleotide exchange factor 15
ARHGEF16	Rho guanine nucleotide exchange factor 16
ARHGEF18	Rho guanine nucleotide exchange factor 18
ARHGEF2	Rho guanine nucleotide exchange factor 2
ARHGEF3	Rho guanine nucleotide exchange factor 3
ARHGEF4	Rho guanine nucleotide exchange factor 4
ARHGEF6	Rho guanine nucleotide exchange factor 6
ARHGEF7	Rho guanine nucleotide exchange factor 7
ARPC3	Actin-related protein 2/3 complex subunit 3
ARPC4	Rho guanine nucleotide exchange factor 4
ATM	ATM serine/threonine kinase
ATP1A1	Sodium/potassium-transporting ATPase subunit alpha-1
ATP1A2	Sodium/potassium-transporting ATPase subunit alpha-2
ATP1B1	Sodium/potassium-transporting ATPase subunit beta-1
ATP1B3	Sodium/potassium-transporting ATPase subunit beta-3
ATP2A2	Sarcoplasmic/endoplasmic reticulum calcium ATPase 2
ATP2B1	Sodium/potassium-transporting ATPase subunit beta-3
BAG2	BAG family molecular chaperone regulator 2
BAK1	Bcl-2 homologous antagonist/killer
BBC3	Bcl-2-binding component 3
BCAP31	B-cell receptor-associated protein 31
BCL2	Apoptosis regulator BCL2
BCL2L1	Bcl-2-like protein 1
BCL2L11	Bcl-2-like protein 11
BCL9	B-cell CLL/lymphoma 9 protein
BMP2	Bone morphogenetic protein 2
BMP7	Bone morphogenetic protein 7
BMPR1B	Bone morphogenetic protein receptor type-1B
BMPR2	Bone morphogenetic protein receptor type-2
C14orf166	UPF0568 protein C14orf166
CALX	Calnexin
CAMK2D	Calcium/calmodulin-dependent protein kinase type II subunit delta
CAMK2G	Calcium/calmodulin-dependent protein kinase type II subunit gamma
CASP10	Caspase-10
CASP6	Caspase-&
CASP7	Caspase-/
CBL	E3 ubiquitin-protein ligase CBL
CCND1	Cyclin D1
CCND2	Cyclin D2
CCNE1	G1/S-specific cyclin-E1
CD44	CD44 antigen
CD81	CD81 antigen
CDC25A	Cell division control protein “% homolog
CDC42	Cell division control protein 42 homolog
CDH2	Cadherin 2, Ncad
CDK2	cyclin dependent kinase 2
CDK6	Cyclin dependent kinase 6
CDKN1A	Cyclin dependent kinase inhibitor 1A
CDKN1B	Cyclin dependent kinase inhibitor 1B
CDKN2A	Cyclin dependent kinase inhibitor 2A
CDKN2B	Cyclin dependent kinase inhibitor 2B
CFL1	Cofilin-1

Table S6 continued

Synonym	Full name
CLD3	Claudin-3
CLTC	Clathrin heavy chain 1
COPA	Coatomer subunit alpha
CPSF6	Cleavage and polyadenylation specificity factor subunit 6
CRABP2	Cellular retinoic acid-binding protein 2
CRK	Adapter molecule crk
CSNK2A1	Casein kinase II subunit alpha
CSPG4	Chondroitin sulfate proteoglycan 4
CTCF	Transcriptional repressor CTCF
DDX1	ATP-dependent RNA helicase DDX1
DDX17	ATP-dependent RNA helicase DDX17
DDX21	ATP-dependent RNA helicase DDX21
DDX23	Probable ATP-dependent RNA helicase DDX23
DDX5	Probable ATP-dependent RNA helicase DDX5
DHX15	Pre-mRNA-splicing factor ATP-dependent RNA helicase DHX15
DHX9	ATP-dependent RNA helicase A
DIABLO	Diablo homolog
DICER1	Endoribonuclease Dicer
DIRAS3	GTP-binding protein Di-Ras3
DLAT	Dihydrolipoyllysine-residue acetyltransferase component of pyruvate dehydrogenase complex
DUSP12	Dual specificity protein phosphatase 12
DVL3	Dishevelled segment polarity protein 3
E2F1	Transcription factor E2F1
E2F2	Transcription factor E2F2
E2F3	Transcription factor E2F3
E2F5	Transcription factor E2F5
E2F6	Transcription factor E2F6
EGFR	Epidermal growth factor receptor
EIF2AK2	Interferon-induced, double-stranded RNA-activated protein kinase
ELAV1	ELAV-like protein 1
ELK1	ETS domain-containing protein Elk-1
EP300	Histone acetyltransferase p300
ERBB2	erb-b2 receptor tyrosine kinase 2
ETS1	Protein C-ets-1
FABP5	Fatty acid-binding protein
FBRL	rRNA 2'-O-methyltransferase fibrillarin
FGF12	Fibroblast growth factor 12
FGF14	Fibroblast growth factor 14
FGF16	Fibroblast growth factor 16
FGF18	Fibroblast growth factor 18
FGF2	Fibroblast growth factor 2
FGF4	Fibroblast growth factor 4
FGF5	Fibroblast growth factor 5
FGF7	Fibroblast growth factor 7
FGF9	Fibroblast growth factor 9
FGFR1	Fibroblast growth factor receptor 1
FGFR2	Fibroblast growth factor receptor 2
FGFR3	Fibroblast growth factor receptor 3
FGFRL1	Fibroblast growth factor receptor-like 1
FIGF / VEGFD	vascular endothelial growth factor D
FIP1L1	Pre-mRNA 3'-end-processing factor FIP1
FLNA	Filamin-A
FLNB	Filamin-B
FLOT2	Flotillin-2
FOS	Proto-oncogene c-Fos
FOXO1	Forkhead box protein O1
FRS2	Fibroblast growth factor receptor substrate 2
FUS	RNA-binding protein FUS
FZD10	Frizzled-10
FZD2	Frizzled-2
FZD3	Frizzled-3
FZD4	Frizzled-4
FZD5	Frizzled-5
FZD6	Frizzled-6
FZD7	Frizzled-7
FZD8	Frizzled-8 Frizzled-8
GAB1	GRB2-associated-binding protein 1
GAB2	GRB2-associated-binding protein 2
GANT	Gli family zinc finger 1 = GLI1
GNA13	Guanine nucleotide-binding protein subunit alpha-13
GNAI1	G protein subunit alpha i1
GNAI2	Guanine nucleotide-binding protein G(i) subunit alpha-2
GNAI3	Guanine nucleotide-binding protein G(k) subunit alpha
GNAT1	Guanine nucleotide-binding protein G(t) subunit alpha-1
GNB1	Guanine nucleotide-binding protein G(I)/G(S)/G(T) subunit beta
GSK3B	Glycogen synthase kinase-3 beta
GSN	Gelsolin

Table S6 continued

**Synonym**	**Full name**
HBEGF	heparin binding EGF like growth factor
HGF	Hepatocyte growth factor
HIF1A	Hypoxia-inducible factor 1-alpha
HIPK2	Homeodomain-interacting protein kinase 2
HMGA2	High mobility group AT-hook 2
HMOX1	heme oxygenase 1
HRAS	GTPase HRAS
HSP90AA1	Heat shock HSP 90-alpha 1
HSP90B1	Heat shock HSP 90-beta 1
HSPA8	Heat shock cognate 71 kDa protein
ID2	Inhibitor of DNA binding 2, HLH protein
IGF2BP2	Insulin-like growth factor 2 mRNA-binding protein 2
IGF2BP3	Insulin-like growth factor 2 mRNA-binding protein 3
IRS1	Insulin receptor substrate 1
IRS2	Insulin receptor substrate 2
ITGA2	Integrin alpha-2
ITGA3	Integrin alpha-3
ITGA4	Integrin alpha-4
ITGA5	Integrin alpha-5
ITGA6	Integrin alpha-6
ITGAV	Integrin alpha-V
ITGB1	Integrin beta-1
ITGB4	Integrin beta-4
ITPR3	Inositol 1,4,5-trisphosphate receptor type 3
JAG1	Jagged 1
JAK1	Tyrosine-protein kinase JAK1
JAK2	Tyrosine-protein kinase JAK2
KRAS	GTPase KRas
LA	Lupus La protein =SSB
LEF1	Lymphoid enhancer-binding factor 1
LMAN2	Vesicular integral-membrane protein VIP36
LMNA	Prelamin-A/C
LOX	Protein-lysine 6-oxidase
MAML1	Mastermind-like protein 1
MAP2K3	Mitogen-activated protein kinase kinase 3
MAP2K4	Dual specificity mitogen-activated protein kinase kinase 4
MAP2K6	Dual specificity mitogen-activated protein kinase kinase 6
MAP2K7	Dual specificity mitogen-activated protein kinase kinase 7
MAP3K5	Mitogen-activated protein kinase kinase kinase 5
MAPK1	Mitogen-activated protein kinase 1
MAPK10	Mitogen-activated protein kinase 10
MAPK12	Mitogen-activated protein kinase 12
MAPK14	Mitogen-activated protein kinase 14
MAPK9	Mitogen-activated protein kinase 9
MAX	Protein max
MDM2	MDM2 proto-oncigene
MET	MET proto-oncogene receptor tyrosine kinase
MMP2	Matrix metalloproteinase-2
MOV10	Putative helicase MOV-10
MRAS	Ras-related protein M-Ras
MY18A	Unconventional myosin-XVIIIa
MYH10	Myosin-10
MYH14	Myosin-14
MYH3	Myosin-3
MYH9	Myosin-9
MYO1C	Unconventional myosin-Ic
MYO1D	Unconventional myosin-Id
MYO6	Unconventional myosin-VI
NAPEPLD	N-acyl phosphatidylethanolamine phospholipase D
NBN	Nibrin
NCBP1	Nuclear cap-binding protein subunit 1
NCSTN	Nicastrin
NLK	Serine/threonine-protein kinase NLK
NOTCH1	Neurogenic locus notch homolog protein 1
NOTCH2	Neurogenic locus notch homolog protein 2
NPM1	Nucleophosmin
NRAS	GTPase NRas
PABPC1	Polyadenylate-binding protein 1
PAK1	Serine/threonine-protein kinase PAK1
PAK2	Serine/threonine-protein kinase PAK2
PAK6	Serine/threonine-protein kinase PAK6
PAK7	Serine/threonine-protein kinase PAK7
PCBP1	Poly(rC)-binding protein 1
PCBP2	Poly(rC)-binding protein 2
PDCD6	Programmed cell death protein 6
PDHB	Pyruvate dehydrogenase E1 component subunit beta
PGAM5	Serine/threonine-protein phosphatase PGAM5

Table S6 continued

**Synonym**	**Full name**
PGF	placental growth factor
PIK3C2A	Phosphatidylinositol 4-phosphate 3-kinase C2 domain-containing subunit alpha
PIK3C2B	Phosphatidylinositol 4-phosphate 3-kinase C2 domain-containing subunit beta
PIK3CD	Phosphatidylinositol 4,5-bisphosphate 3-kinase catalytic subunit delta isoform
PIK3R1	Phosphoinositide-3-kinase regulatory subunit alpha
PIK3R3	Phosphoinositide-3-kinase regulatory subunit 3
PIK3R5	Phosphoinositide-3-kinase regulatory subunit 5
PLCB1	1-phosphatidylinositol 4,5-bisphosphate phosphodiesterase beta-1
PLD2	phospholipase D2
PLD3	phospholipase D family member 3
PMAIP1	Phorbol-12-myristate-13-acetate-induced protein 1
POP1	Ribonucleases P/MRP protein subunit POP1
PPP1CA	Serine/threonine-protein phosphatase PP1-alpha catalytic subunit
PRKACB	cAMP-dependent protein kinase catalytic subunit beta
PRKAR1A	cAMP-dependent protein kinase type I-alpha regulatory subunit
PRKAR2A	cAMP-dependent protein kinase type II-alpha regulatory subunit
PRKD3	Serine/threonine-protein kinase D3
PRKRA	Interferon-inducible double-stranded RNA-dependent protein kinase activator A
PRPF8	Pre-mRNA-processing-splicing factor 8
PSEN1	Presenilin-1
PTGS2	prostaglandin-endoperoxide synthase 2
PTPN11	Protein tyrosine phosphatase, non-receptor type 11
PUF60	Poly(U)-binding-splicing factor PUF60
PYGO2	Pygopus homolog 2
RAB2B	RAB2B, member RAS oncogene family
RAB7A	RAB7A, member RAS oncogene family
RAC1	Rac family small GTPase 1
RAC3	Ras-related C3 botulinum toxin substrate 3
RACK1	Receptor of activated protein C kinase 1
RAF1	RAF proto-oncogene serine/threonine-protein kinase
RALA	RAS like proto-oncogene A
RALBP1	RalA-binding protein 1
RAP1B	RAP1B, member RAS oncogene family
RAP2B	RAP2B, member RAS oncogene family
RAPGEF1	Rap guanine nucleotide exchange factor 1
RAPGEF3	Rap guanine nucleotide exchange factor 3
RASGRF1	Ras protein specific guanine nucleotide releasing factor 1
RASGRF2	Ras-specific guanine nucleotide-releasing factor 2
RASGRP1	RAS guanyl-releasing protein 1
RB1	Retinoblastoma-associated protein
RBMX	RNA-binding motif protein, X chromosome
RELA	Transcription factor p65
RHOA	Transforming protein RhoA
RHOC	Rho-related GTP-binding protein RhoC
RHOG	Rho-related GTP-binding protein RhoG
RHOQ	Rho-related GTP-binding protein RhoQ
RHOT2	Mitochondrial Rho GTPase 2
RND2	Rho-related GTP-binding protein RhoN
RND3	Rho-related GTP-binding protein RhoE
RRAS2	Ras-related protein R-Ras2
SEC23B	Protein transport protein Sec23B
SF3B1	Splicing factor 3B subunit 1
SFPQ	Splicing factor, proline- and glutamine-rich
SIN3A	SIN3 transcription regulator family member A
SKIV2L2	Mtr4 exosome RNA helicase = MTRX
SLC12A2	Solute carrier family 12 member 2
SLC1A5	4F2 cell-surface antigen heavy chain
SLC2A1	Solute carrier family 2, facilitated glucose transporter member
SLC3A2	solute carrier family 3 member 2
SLC7A5	solute carrier family 7 member 5
SMAD2	Mothers against decapentaplegic homolog 2
SMAD4	Mothers against decapentaplegic homolog 4
SMAD5	Mothers against decapentaplegic homolog 5
SMAD6	Mothers against decapentaplegic homolog 6
SMAD7	Mothers against decapentaplegic homolog 7
SMO	Smoothened homolog
SMURF1	E3 ubiquitin-protein ligase SMURF1
SNAI1	Zinc finger protein SNAI1
SNRNP200	U5 small nuclear ribonucleoprotein 200 kDa helicase
SOS1	Son of sevenless homolog 1
SOS2	Son of sevenless homolog 2
SPF45	Splicing factor 45
SPTAN1	Spectrin alpha chain, non-erythrocytic 1
SPTBN1	Spectrin beta chain, non-erythrocytic 1
SRSF9	Serine/arginine-rich splicing factor 9
SSR1	Translocon-associated protein subunit alpha
SSR4	Translocon-associated protein subunit delta4

Table S6 continued

**Synonym**	**Full name**
STAT3	Signal transducer and activator of transcription 3
STX4	Syntaxin-4
SUFU	Suppressor of fused homolog
SUV39H1	Histone-lysine N-methyltransferase SUV39H1
SYNGAP1	Ras/Rap GTPase-activating protein SynGAP
TAB1	TGF-beta-activated kinase 1 and MAP3K7-binding protein
TCF4	Transcription factor 4
TCF7L1	Transcription factor 7-like 1
TCF7L2	Transcription factor 7-like 2
TFB1M	Dimethyladenosine transferase 1
TFDP1	transcription factor Dp-1
TFRC	Transferrin receptor protein 1
TGFA	transforming growth factor alpha
TGFB2	Transforming growth factor beta-2
TGFBR1	TGF-beta receptor type-1
TGFBR2	TGF-beta receptor type-2
TMED10	Transmembrane emp24 domain-containing protein 10
TMED2	Transmembrane emp24 domain-containing protein 2
TMED4	Transmembrane emp24 domain-containing protein 4
TMED9	Transmembrane emp24 domain-containing protein 9
TP53	Cellular tumor antigen p53
TSPAN8	Tetraspanin-8
TSR1	Pre-rRNA-processing protein TSR1 homolog
TUBA1B	Tubulin alpha-1B chain
TUBA4A	Tubulin alpha-4A chain
TUBA4B	Tubulin alpha-4B chain
TWIST1	Signal transducer and activator of transcription 1, 3
U2AF1	Splicing factor U2AF 35 kDa subunit
U2AF2	Splicing factor U2AF 65 kDa subunit
VAPA	Vesicle-associated membrane protein-associated protein A
VEGFA	vascular endothelial growth factor A
WNT1	Proto-oncogene Wnt, wingless-type MMTV integration site family 1
WNT10A	Protein Wnt-10a
WNT16	Protein Wnt-16
WNT2B	Protein Wnt-2b
WNT3A	Protein Wnt-3a
WNT4	Protein Wnt-4
WNT5A	Protein Wnt-5a
WNT5B	Protein Wnt-5b
WTAP	Pre-mRNA-splicing regulator WTAP
XIAP	E3 ubiquitin-protein ligase XIAP
XRN2	5'-3' exoribonuclease 2
YBX1	Nuclease-sensitive element-binding protein 1
YBX3	Y-box-binding protein 3
YWHAE	14-3-3 protein epsilon
YWHAZ	14-3-3 protein zeta/delta
ZEB1	Zinc finger E-box-binding homeobox 1
ZEB2	Zinc finger E-box-binding homeobox 2

Table S7

Noncoding RNA: full names of synonyms (alphabetic) and functional annotation

Symbol	Gene Name[a]	available functional informations[b]
ADIRF-AS1	ADIRF antisense RNA 1	none
AFAP1-AS1	AFAP1 antisense RNA 1	invasion, proliferation, EMT, c-myc, cyclin D1, cemiR-181a -> RAP1B up
APCDD1L-AS1	APCDD1L antisense RNA 1 (hh))	cancer
ARRDC1-AS1	ARRDC1 antisense RNA 1	none
BAIAP2-AS1	BAIAP2 antisense RNA 1 (hh)	none
CTBP1-AS2	CTBP1 antisense RNA 2 (hh)	none
CUTALP	cutA divalent cation tolerance homolog-like pseudogene	none
DBH-AS1	DBH antisense RNA 1	cancer, induced by HBX -> MAPK activation
DHRS4-AS1	DHRS4 antisense RNA 1	controls 3 DHRS4 genes, physically interacts with promoter through chromosomal looping
DLGAP1-AS1	DLGAP1 antisense RNA 1	none
DLGAP1-AS2	DLGAP1 antisense RNA 2	none
EIF3J-AS1	EIF3J antisense RNA 1 (hh)	none
ELFN1-AS1	ELFN1 antisense RNA 1	Myclo-2, myc-regulated lncRNA, regulates myc target genes (CDKN1A, CDKN2B), RNA binding proteins HuR and hbRNPK, transformation and tumorigenesis
EPB41L4A-AS1	EPB41L4A antisense RNA 1	calcification
FAM83H-AS1	FAM83H antisense RNA 1 (hh)	regulates prolif.& invasion through NOTCH, MET/EGFR signaling
FBXL19-AS1	FBXL19 antisense RNA 1 (hh)	sponges miR-203
FGD5-AS1	FGD5 antisense RNA 1	none
FIRRE	firre intergenic repeating RNA element	interacts with hrnU and coats chromosomes (maintenance of repressive chromatin), posttransciptional stability mRNA during innate immune response
FOXD2-AS1	FOXD2 antisense RNA 1 (hh)	regulates EMT, NOTCH, Wnt/β-catenin, interacts with miR-185-5p and -363-5p/S100A1
GATA2-AS1	GATA2 antisense RNA 1	binds GATA family of zink finger transcription factors
GOLGA2P5	golgin A2 pseudogene 5	proliferation and TERT transcription
HAGLR	HOXD antisense growth-associated lnc RNA	JAK2/STAT3 pathway, targets miR-133a-3p -> Wnt/β-catenin
HOTAIRM1	HOXA transcript antisense RNA, myeloid-specific 1	transcription induced by RA, regulate HOXA gene and b2-integrins
HOTTIP	HOXA distal transcript antisense RNA	regulates HOXA gene transcription, HIF1α/HOTTIP/miR-101/ZEB1 complex
HOXA11-AS	HOXA11 antisense RNA	associates with chromatin factors (Polycomb repressive complex), sponges miRNAmiR125a-5p, 124-3p, 1297
HOXA-AS2	HOXA cluster antisense RNA 2	interact with enhancer of zeste homolog 2 Polycomb repressive complex
HOXA-AS3	HOXA cluster antisense RNA 3	competitively binds hnRNP A1 antagonizing PKM splicing, critical in cancer metabolic reprogramming
HOXB-AS3	HOXB cluster antisense RNA 3	CoCa suppression
ILF3-AS1	ILF3 antisense RNA 1 (hh)	sponges miR-200b/a/429
JHDM1D-AS1	JHDM1D antisense RNA 1 (hh)	none
KCNQ1OT1	KCNQ1 opposite strand/antisense transcript 1	interacts with chromatin regulating transcription of many genes
KRT8P12	keratin 8 pseudogene 12	none
LBX2-AS1	LBX2 antisense RNA 1	none
LHFPL3-AS2	LHFPL3 antisense RNA 2	none
LINC00152	long intergenic non-protein coding RNA 152	binds enhancer of zeste homolog 2, silencing of tumor suppressor genes, sponge miR-16, -103a, -199-5p, -138

Table S7 continued

Symbol	Gene Name[a]	available functional informations[b]
LINC00239	long intergenic non-protein coding RNA 239	none
LINC00261	long intergenic non-protein coding RNA 261	inhibitory, regulates NOTCH1, Hes-1, FOXO1
LINC00467	long intergenic non-protein coding RNA 467	promotes survival, downregulated by N-Myc
LINC00511	long intergenic non-protein coding RNA 511	binds EZH2, suppresses p57
LINC00649	long intergenic non-protein coding RNA 649	none
LINC00659	long intergenic non-protein coding RNA 659	none
LINC00662	long intergenic non-protein coding RNA 662	none
LINC00665	long intergenic non-protein coding RNA 665	cell cycle pathway
LINC00667	long intergenic non-protein coding RNA 667	none
LINC00847	long intergenic non-protein coding RNA 847	none
LINC00868	long intergenic non-protein coding RNA 868	none
LINC00896	long intergenic non-protein coding RNA 896	none
LINC00920	long intergenic non-protein coding RNA 920	none
LINC00941	long intergenic non-protein coding RNA 941	sponges miR-34a, Snail upregulation, EMT
LINC00963	long intergenic non-protein coding RNA 963	sponges miR-6608 -> NACC1, PGK1/mTOR pathway, Foxo pathway
LINC01124	long intergenic non-protein coding RNA 1124	none
LINC01137	long intergenic non-protein coding RNA 1137	tumor suppressive nexus
LINC01184	long intergenic non-protein coding RNA 1184	stress response
LINC01278	long intergenic non-protein coding RNA 1278	none
LINC01468	LNCAROD, long intergenic non-protein coding RNA 1468	activates DKK1
LINC01578	long intergenic non-protein coding RNA 1578	none
LINC-PINT	long intergenic non-protein coding RNA, p53 induced transcript	inhibits invasion
LOC100288175	uncharacterized LOC100288175	none
LOC100506098	uncharacterized LOC100506098	none
LOC100506688	uncharacterized LOC100506688	none
LOC103344931	uncharacterized LOC103344931	none
LOC648987	uncharacterized LOC648987	none
LOC728554	THO complex 3 pseudogene	none
LOC728730	MAP4K3 divergent transcript	none
LRRC37A16P	leucine rich repeat containing 37 member A16, pseudogene	none
SNHG29	small nucleolar RNA host gene 29	none
MCM3AP-AS1	MCM3AP antisense RNA 1	ce-RNA network in glioblastoma
MIR4435-2HG	MIR4435-2 host gene	regulates MDM2/p53 signaling
MIR4458HG	MIR4458 host gene	antagonizes DHX36, inhibits proliferation and migration
MIR600HG	MIR600 host gene	none
MNX1-AS1	CCAT5, MNX1 antisense RNA 1 (hh)	antagonizes DHX36, inhibits proliferation and migration, MYC-regulated
NNT-AS1	NNT antisense RNA 1	regulates miR-129-5p, miR-142-3p/ZEB1, miR-363/CDK6, Wnt/β-catenin signaling

Table S7 continued

**Symbol**	**Gene Name**[a]	**available functional informations**[b]
NOP14-AS1	NOP14 antisense RNA 1	none
NORAD	non-coding RNA activated by DNA damage	sponges miR-125a, -373, -615-3p, -590-3pp/SIP1, binds PUM1 & PUM2, upregulates TGFβ-signaling inEMT, RhoA expr.
NUTM2A-AS1	NUTM2A antisense RNA 1	none
OIP5-AS1	OIP5 antisense RNA 1	inversely correlated with miR-4110, which targets KLLF10 that activates the PTEN/PI3K/AKT pathway
OLMALINC	oligodendrocyte maturation-associated long intergenic non-coding RNA	controls proliferation
OTUD6B-AS1	OTUD6B antisense RNA 1 (hh)	none
PAX8-AS1	PAX8 antisense RNA 1	increased cancer risk
PAXIP1-AS1	PAXIP1 antisense RNA 1 (hh)	none
PCBP1-AS1	PCBP1 antisense RNA 1	none
PIK3CD-AS2	PIK3CD antisense RNA 2	none
PPP1R26-AS1	PPP1R26 antisense RNA 1	oncogenic
PROX1-AS1	PROX1 antisense RNA 1	gllucose homeostasis, association with type2 diabetes, Alzheimer, AIDS
PRR34-AS1	PRR34 antisense RNA 1	none
PRSS3P1	protease, serine 3 pseudogene 1	none
PSMA3-AS1	PSMA3 antisense RNA 1	none
PTOV1-AS1	PTOV1 antisense RNA 1	none
PVT1	Pvt1 oncogene (non-protein coding)	regulates p53, sponges miR-424-5p -> release of INCENP (inner centromer protein)
RNASEH1-AS1	RNASEH1 antisense RNA 1	antagonizes DHX36, CoCa migration
RNF157-AS1	RNF157 antisense RNA 1	none
RP9P	retinitis pigmentosa 9 pseudogene	interferes with ubiquitin ligase PRP119
RPARP-AS1	RPARP antisense RNA 1	none
RPL10P3	ribosomal protein L10 pseudogene 3	none
RPL13AP5	ribosomal protein L13a pseudogene 5	none
RPL13P12	ribosomal protein L13 pseudogene 12	none
RPL14P1	ribosomal protein L14 pseudogene 1	none
RPL18AP3	ribosomal protein L18a pseudogene 3	none
RPL32P3	ribosomal protein L32 pseudogene 3	splicing regulation in prostate cancer
RPL3P4	ribosomal protein L3 pseudogene 4	none
RPL4P4	ribosomal protein L4 pseudogene 4	none
RPS13P2	ribosomal protein S13 pseudogene 2	none
RPS23P8	ribosomal protein S23 pseudogene 8	none
RPS28P7	ribosomal protein S28 pseudogene 7	none
RPS4XP22	ribosomal protein S4X pseudogene 22	none
SLC25A25-AS1	SLC25A25 antisense RNA 1	low expression promotes proliferation, chemoresistance and EMT in CoCa
SLC2A1-AS1	SLC2A1 antisense RNA 1	none
SNHG1	small nucleolar RNA host gene 1	promotes apoptosis resistance & proliferation via PI3K/Akt activation, correlates with Wnt/β-catenin expression
SNHG10	small nucleolar RNA host gene 10	none
SNHG12	small nucleolar RNA host gene 12	inversely correlates with miR-320, promotes growth and apoptosis resistance -> increased CDK4, CDK6, CCND1 & reduced caspase 3 expression

Table S7 continued

**Symbol**	**Gene Name**[a]	**available functional informations**[b]
SNHG15	small nucleolar RNA host gene 15	promotes growth, tumorigenicity and apoptosis resistance, represses P15 and KLF2 by EZH2 binding (histone H3 methylation), correlates with invasiveness and metastasis by MMP2 & MMP9 regulation
SNHG16	small nucleolar RNA host gene 16	regulates Wnt transcription factors, act as ce-RNA via AGO/miRNA binding, engaged in lipid metabolism regulation
SNHG17	small nucleolar RNA host gene 17	correlates with tumor progression, epigenetically suppresses CDKN1C / P57 by binding to EZH2
SNHG19	small nucleolar RNA host gene 19	none
SNHG3	small nucleolar RNA host gene 3	silences KLF2 & p21, promotes proliferation, suppresses apoptosis, regulates energy metabolism through miRNAs & EIF4AIII
SNHG5	small nucleolar RNA host gene 5	promotes apoptosis resistance by sponging microRNAs, stabilizes mRNAs, including SPATS2 (spermatogenesis associated serine rich 2) blocking degradation by staufen double-stranded RNA binding protein 1
SNHG6	small nucleolar RNA host gene 6	sponges miR-26a/b, modulating TAK1 (transforming growth factor-β-activated kinase 1) expression, sponges miR-101-3p (release of ZEB1 from repression), silences p27 by recruiting EZH2 to the promoter of p27, promotes p21 transcription via the JNK pathway and EZH2 recruitment
SNHG7	small nucleolar RNA host gene 7	engaged in p15 and p16 regulation
SNHG8	small nucleolar RNA host gene 8	sponges miR-149-5p, pro-oncogenic in EBV-associated gastric cancer
SNHG9	small nucleolar RNA host gene 9	none
SVIL-AS1	SVIL antisense RNA 1	none
THAP9-AS1	THAP9 antisense RNA 1	none
THUMPD3-AS1	THUMPD3 antisense RNA 1	promotes PTEN expression
TMEM147-AS1	TMEM147 antisense RNA 1	none
TNRC6C-AS1	TNRC6C antisense RNA 1	regulates UNC5B through miR-129-5p sponging -> proliferation, migration and invasion
TRIM52-AS1	TRIM52 antisense RNA 1 (hh)	downregulation suppresses renal cell carcinoma
TRPM2-AS	TRPM2 antisense RNA	downregulation inhibits cisplatin resistance via p53-p66 shc pathway activation
UBA6-AS1	UBA6 antisense RNA 1 (hh)	none
VPS9D1-AS1	VPS9D1 antisense RNA 1	associates with HNRNPK (heterogeneous nuclear ribonucleoprotein K) stabilizing CDK6 expression, promotes G1-S transition, may act downstream of Wnt/Myc signaling
WAC-AS1	WAC antisense RNA 1 (hh)	none
ZBED5-AS1	ZBED5 antisense RNA 1	none
ZEB1-AS1	ZEB1 antisense RNA 1	binds KMTA2 (lysine methyltransferase 2A), promotes histone modifications, correlates with tumor progression regulating cell cycle inhibitors (cyclin D1, CDK2), regulates migration & invasion via activating EMT through up-regulating ZEB1, MMP2, MMP9, N-cadherin, & integrin β1 and decreasing E-cadherin, promotes apoptosis resistance by downregulating Bax and upregulating Bcl-2 expression
ZFAS1	ZNFX1 antisense RNA 1	oncogenic, regulatory network involving miR-329 & -27a sponging, enganged in Wnt/β-catenin signaling and KLF2 & NKD2 repression
ZNF670-ZNF695	ZNF670-ZNF695 readthrough (NMD candidate)	none
ZNF702P	zinc finger protein 702, pseudogene	none

[a] hh: head to head
[b] please search data bases for detailed information and updates

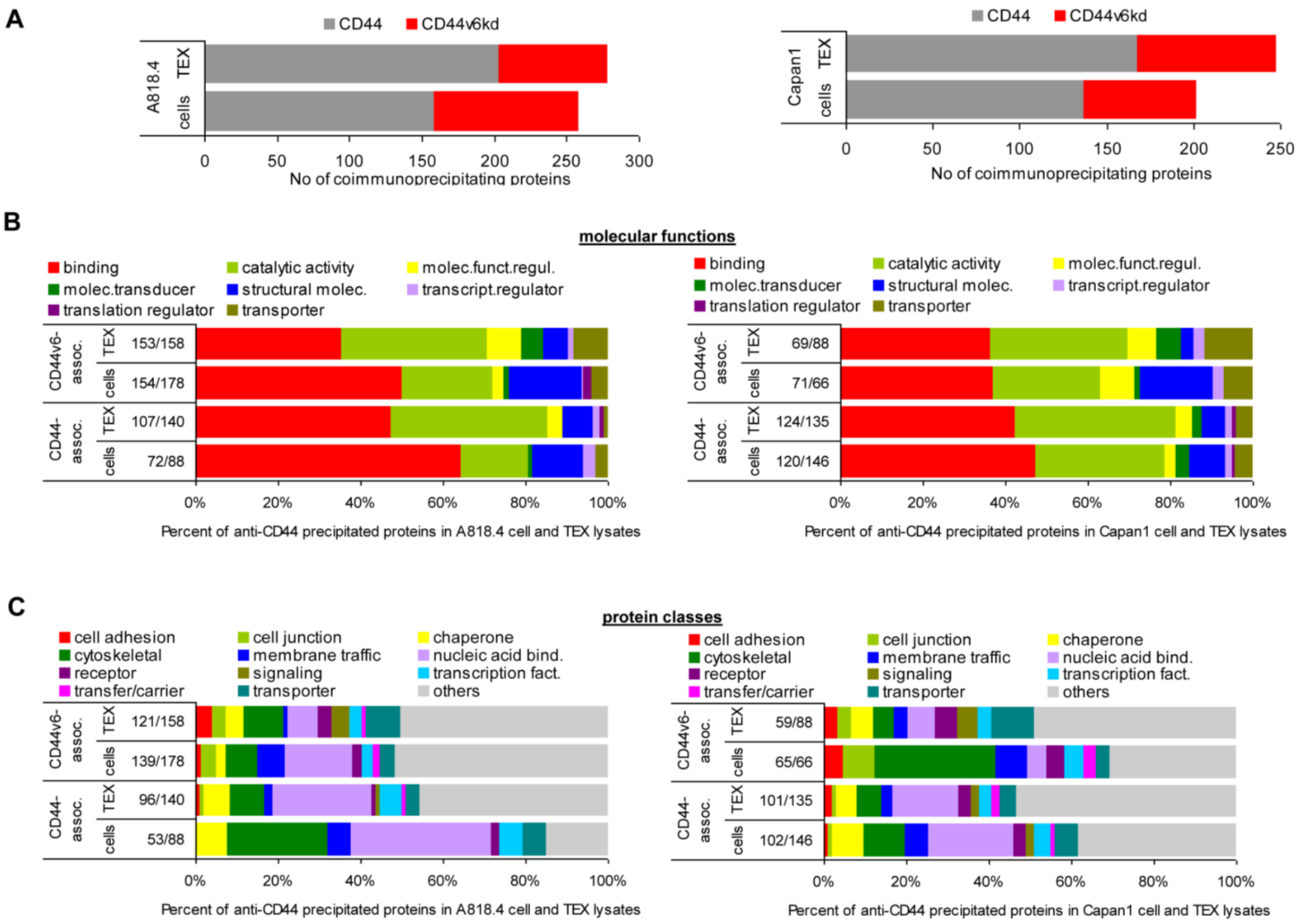

Figure S1 Classification of CD44- and CD44v6-associated molecules in cells and TEX. (A) A818.4- and Capan1-wt and -CD44v6kd cell and TEX were mildly lysed (Lubrol) and precipitated with anti-CD44v6 (wt) or anti-CD44 (CD44v6kd). Precipitates were analyzed by nanoLC-ESI-MS/MS. The number of proteins coimmunoprecipitating exclusively with anti-CD44 or anti-CD44v6 is shown; (B,C) Panther pathway analysis according to molecular functions and protein classes of proteins recovered in cell and/or TEX lysate precipitates (listed in Table S3 for A818.4 cells and TEX).
Recovery of a comparable number of proteins coimmunoprecipitating with CD44 or CD44v6 in cells and TEX of two PaCa lines supports the reliability of the nanoLC-ESI-MS/MS analysis. The corresponding recovery of CD44v6 coimmunoprecipitating molecules in cell and TEX lysates argues against an active contribution of CD44v6 in the TEX transfer. However, minor differences in molecular functions and protein classes of CD44- versus CD44v6-coimmunoprecipitating molecules in cells and TEX demanded for a detailed analysis.

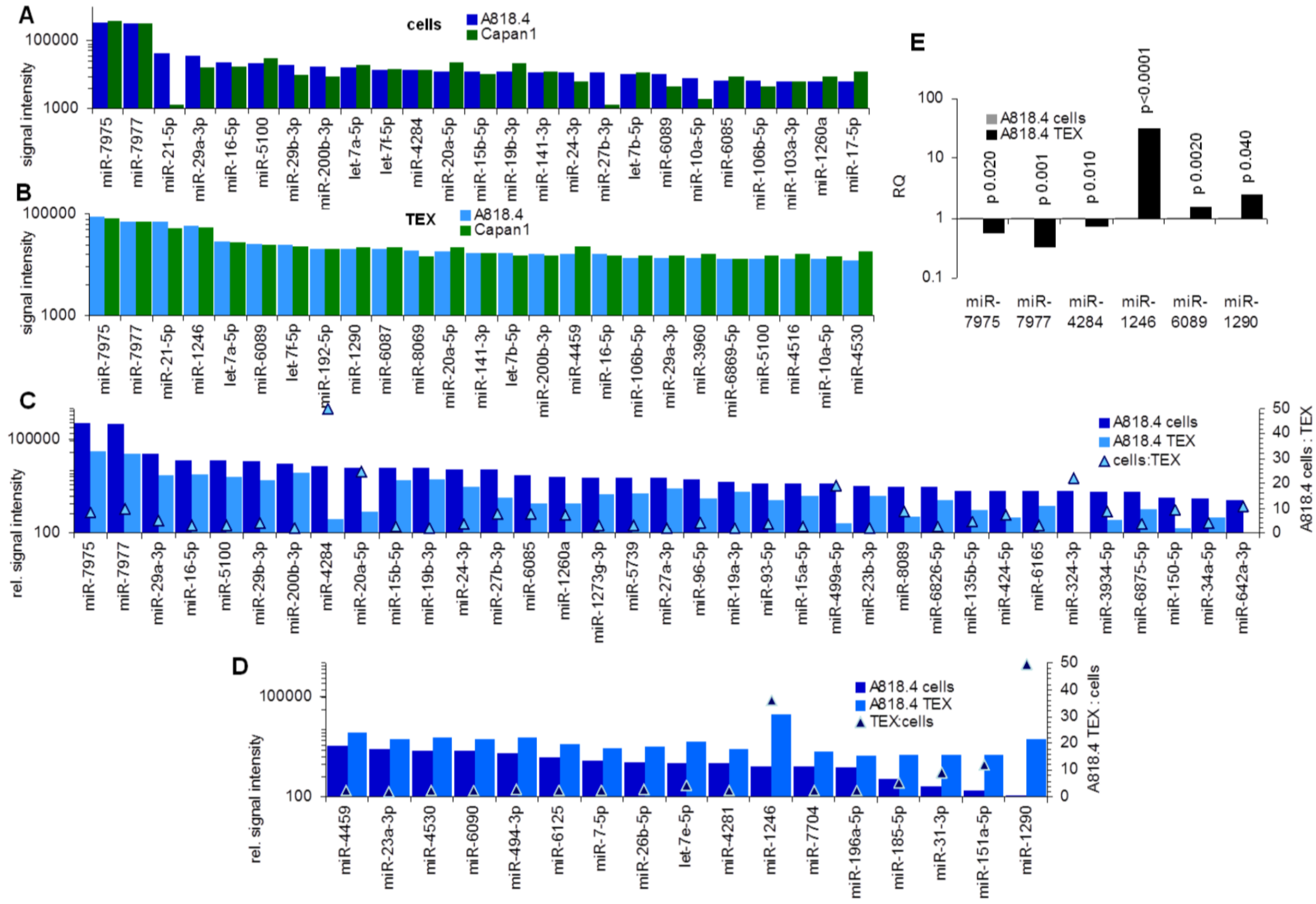
A
cells
A818.4
Capan1
signal intensity
100000
1000
miR-7975
miR-7977
miR-21-5p
miR-29a-3p
miR-16-5p
miR-5100
miR-29b-3p
miR-200b-3p
let-7a-5p
let-7f-5p
miR-4284
miR-20a-5p
miR-15b-5p
miR-19b-3p
miR-141-3p
miR-24-3p
miR-27b-3p
let-7b-5p
miR-6089
miR-10a-5p
miR-6085
miR-106b-5p
miR-103a-3p
miR-1260a
miR-17-5p
B
TEX
A818.4
Capan1
signal intensity
100000
1000
miR-7975
miR-7977
miR-21-5p
miR-1246
let-7a-5p
miR-6089
let-7f-5p
miR-192-5p
miR-1290
miR-6087
miR-8069
miR-20a-5p
miR-141-3p
let-7b-5p
miR-200b-3p
miR-4459
miR-16-5p
miR-106b-5p
miR-29a-3p
miR-3960
miR-6869-5p
miR-5100
miR-4516
miR-10a-5p
miR-4530
C
A818.4 cells
A818.4 TEX
cells:TEX
rel. signal intensity
100000
100
A818.4 cells : TEX
50
40
30
20
10
0
miR-7975
miR-7977
miR-29a-3p
miR-16-5p
miR-5100
miR-29b-3p
miR-200b-3p
miR-4284
miR-20a-5p
miR-15b-5p
miR-19b-3p
miR-24-3p
miR-27b-3p
miR-6085
miR-1260a
miR-1273g-3p
miR-5739
miR-27a-3p
miR-96-5p
miR-19a-3p
miR-93-5p
miR-15a-5p
miR-499a-5p
miR-23b-3p
miR-8089
miR-6826-5p
miR-135b-5p
miR-424-5p
miR-6165
miR-324-3p
miR-3934-5p
miR-6875-5p
miR-150-5p
miR-34a-5p
miR-642a-3p
D
A818.4 cells
A818.4 TEX
TEX:cells
rel. signal intensity
100000
100
A818.4 TEX : cells
50
40
30
20
10
0
miR-4459
miR-23a-3p
miR-4530
miR-6090
miR-494-3p
miR-6125
miR-7-5p
miR-26b-5p
let-7e-5p
miR-4281
miR-1246
miR-7704
miR-196a-5p
miR-185-5p
miR-31-3p
miR-151a-5p
miR-1290
E
A818.4 cells
A818.4 TEX
RQ
100
10
1
0.1
p 0.020
p 0.001
p 0.010
p<0.0001
p 0.0020
p 0.040
miR-7975
miR-7977
miR-4284
miR-1246
miR-6089
miR-1290

F

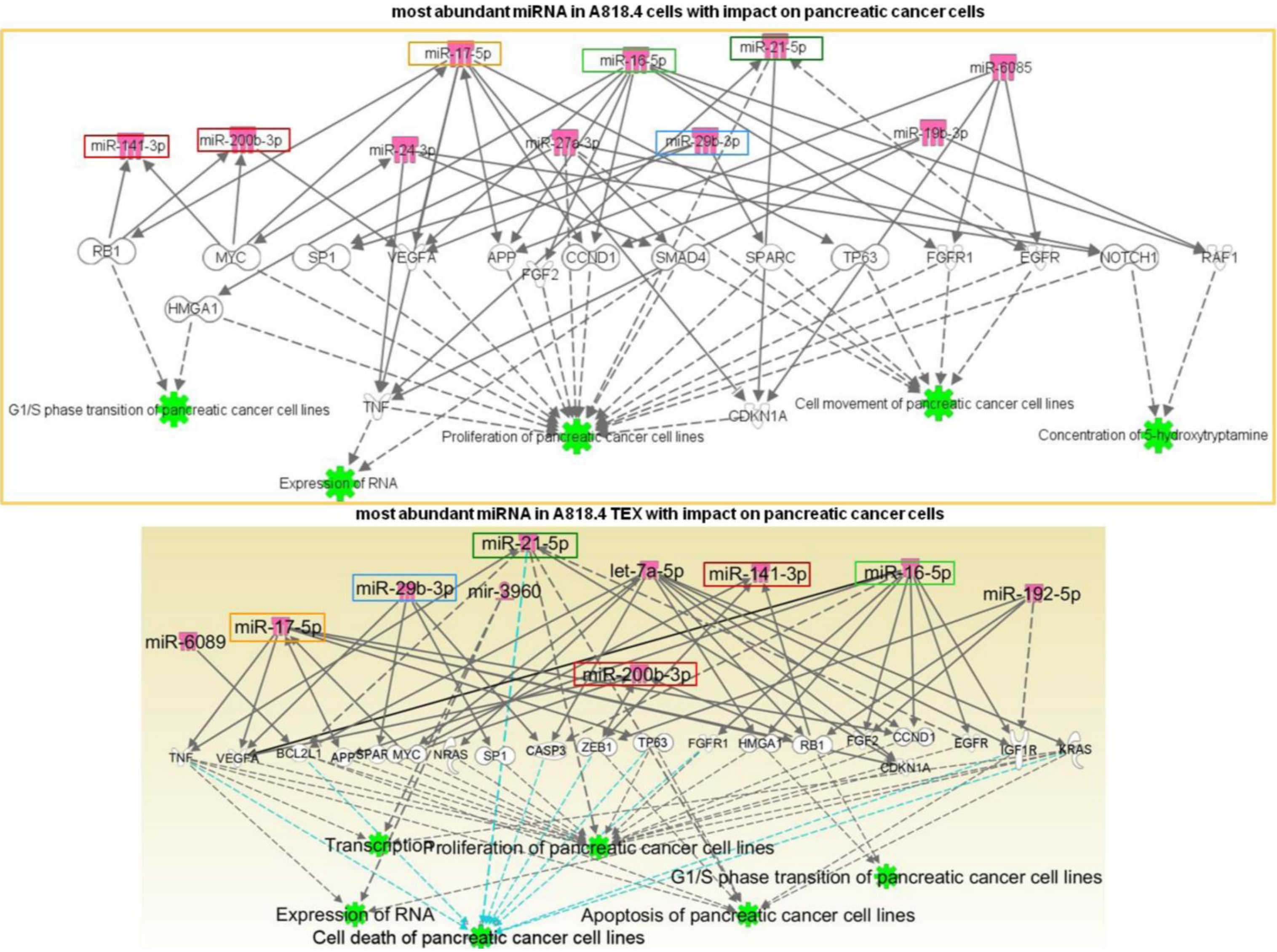
most abundant miRNA in A818.4 cells with impact on pancreatic cancer cells
miR-17-5p
miR-16-5p
miR-21-5p
miR-6085
miR-141-3p
miR-200b-3p
miR-24-3p
miR-27a-3p
miR-29b-3p
miR-19b-3p
RB1
MYC
SP1
VEGFA
APP
FGF2
CCND1
SMAD4
SPARC
TP63
FGFR1
EGFR
NOTCH1
RAF1
HMGA1
G1/S phase transition of pancreatic cancer cell lines
TNF
Proliferation of pancreatic cancer cell lines
CDKN1A
Cell movement of pancreatic cancer cell lines
Concentration of 5-hydroxytryptamine
Expression of RNA
most abundant miRNA in A818.4 TEX with impact on pancreatic cancer cells
miR-21-5p
let-7a-5p
miR-141-3p
miR-16-5p
miR-29b-3p
mir-3960
miR-192-5p
miR-17-5p
miR-6089
miR-200b-3p
TNF
VEGFA
BCL2L1
APP
SPARC
MYC
NRAS
SP1
CASP3
ZEB1
TP63
FGFR1
HMGA1
RB1
FGF2
CCND1
CDKN1A
EGFR
IGF1R
KRAS
Transcription
Proliferation of pancreatic cancer cell lines
G1/S phase transition of pancreatic cancer cell lines
Expression of RNA
Apoptosis of pancreatic cancer cell lines
Cell death of pancreatic cancer cell lines

G

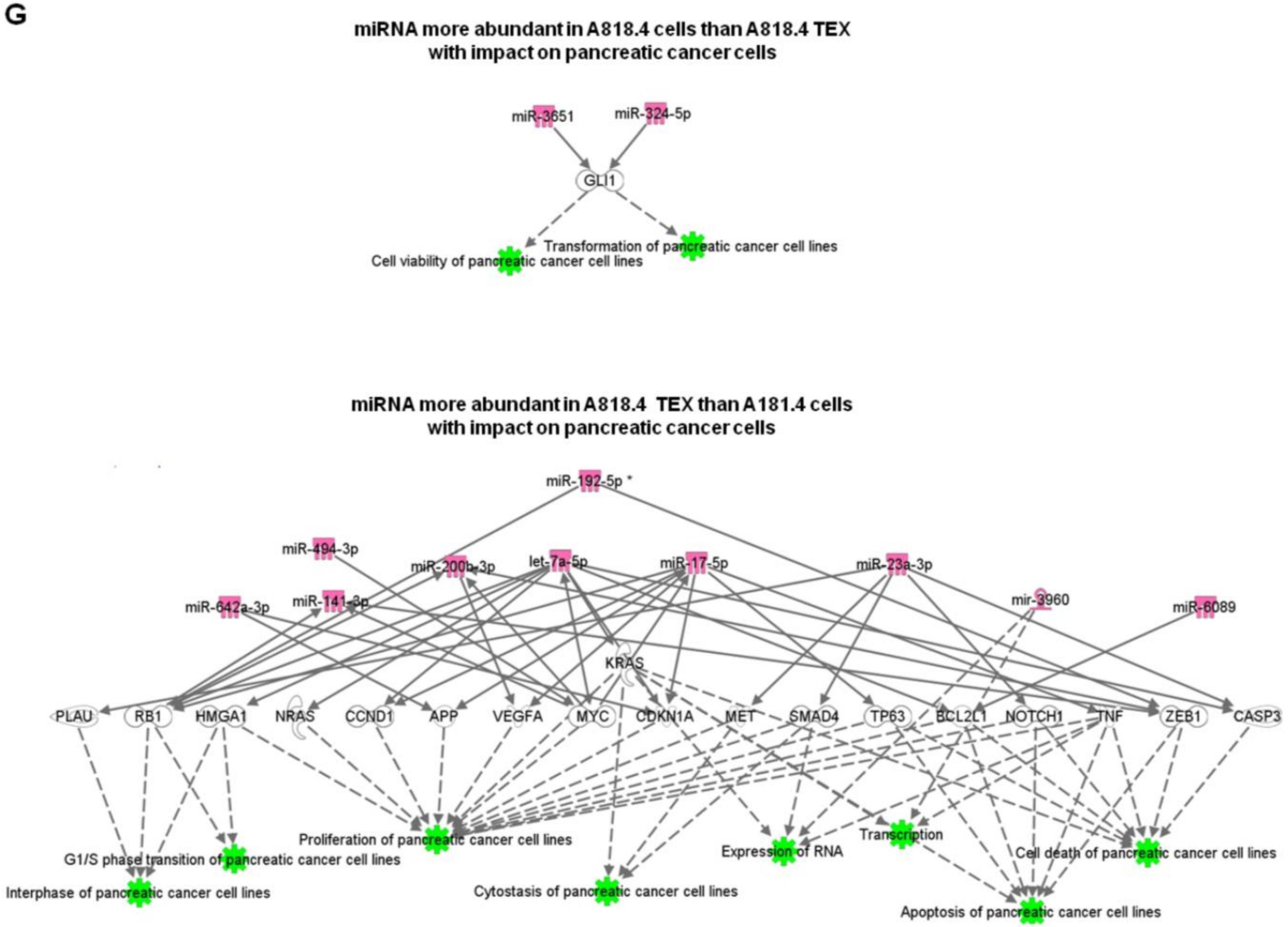

Figure S2 miRNA profile of PaCa cells and TEX. MiRNA was analyzed by DS. (A) Most abundant miRNA in two PaCa lines; (B) most abundant miRNA in two PaCa TEX; (C) miRNA that expression is significantly reduced in TEX compared to cells and (D) miRNA that expression is significantly higher in TEX than cells; (E) qRT-PCR examples of miRNA with significantly different expression in cells versus TEX; RQ values (mean±SD, triplicates) of TEX in comparison to cells; p-values are indicated; (F) predicted targets of most abundant miRNA in cells and/or TEX (high recovery in cells and TEX: framed) and (G) predicted targets of miRNA distinctly recovered in cells versus TEX were selected by the KEGG data base according to an impact on pancreatic cancer cells. mRNA predictions are based on miRNA and target scan databases. In (F and G) the pancreatic cancer related functions of the predicted targets are shown. (full name of synonyms in Table S6).
MiRNA recovery in two pancreatic lines and TEX rarely differs. Instead, miRNA recovery differed between cells versus TEX indicating nonrandom recruitment into TEX. Predicted targets engaged in proliferation are most abundant in cells and TEX. In TEX, a considerable number of miRNA targets are engaged in cell death and apoptosis. Only two abundant miRNA engaged in pancreatic cancer progression are higher in cells than TEX, both targeting the transcription factor GLI1. Instead, several miRNA that control targets engaged in PaCa proliferation, cell death, and apoptosis are recovered at a higher level in TEX than cells. The abundance of miRNA targets engaged in signaling including EMT-related transcription factors should be noted.

A

Predicted mRNA targets for miRNA higher in A818.4 than A818.4-CD44v6kd cells (cancer-related signaling)

miR-125a-5p	miR-151a-3p	miR-17-5p	miR-185-5p	miR-18a-5p	miR-192-5p	miR-194-5p	miR-196a-5p	miR-25-3p	miR-26b-5p	miR-31-5p	miR-324-5p	miR-331-3p	miR-374a/b-	miR-455-3p	miR-7-5p	miR-99b-5p
ABL1	AKT3	AKT3	AKT1	ATM	CBL	AKT2	ABL1	CDC42	E2F2	CDKN2A	AKT2	ERBB2	AKT1	FZD10	BCL2L1	ADCY1
ADCY1	APH1A	ARHGEF10	ARHGEF6	CDK2	PRKaR1A	BMPR1B	ADCY9	CDKN1A	PIK3C2A	E2F2	ARHGEF4	PGF	CCND1		EGFR	BMRP2
APC	WNT1	ARHGEF11	CAMK2D	CDKN1A	PRKD3	CAMK2G	ARHGEF15	E2F3	PIK3R3	HBEGF	DIABLO	PLD2	FGFR2		IRS1	FGF16
ARHGEF1		ARHGEF18	CCNE1	E2F1	ZEB1	E2F3	CDKN1B	FRS2	PTGS2	PIK3C2A	GANZ	RAF1	FRS2		IRS2	FGFR3
BAK1		ARHGEF3	CDC42	E2F2	ZEB2	FGFR3	NRAS	IRS2	RB1		GNAT1	RALA	MAP2K4		PIK3CD	FZD5
BBC3		ARHGEF7	E2F6	E2F3		FOXO1	RALBP1	MAP2K4	SMAD2		HIPK2		PTPN11		PIK3R3	FZD8
BCL2		BCL2	ELK1	FGFR3		FZD5	RASGRP1	NOTCH1	SMAD4		MAP2K3		TFDP1		RAF1	RAP1B
BMPR1B		BCL2LL11	GNA13	FRS2		FZD6		PIK3R3	TGFBR2		RAC3		TGFA		RB1	
BMPR2		BMP2	GSK3B	SMAD2		HBEGF		TGFB2			SMAD2		VEGFA		RELA	
CASP6		CCND1	NBN			MAPK1					TAB1				SIN3A	
CASP7		CCND2	PAK6			PAK2					WNT3A					
CDC25A		CDKN1A	PRKAR2A			PRKAR1A										
CDK6		CRK	RHOA			RAB2B										
CDKN2A		E2F1	RHOG			SUFU										
CDKN2B		E2F2	SYNGAP1													
E2F2		E2F3	VEGFA													
E2F3		E2F5														
FGFR1		FRS2														
FGFR2		FZD3														
GAB2		FZD4														
MAPK12		FZD7														
MAPK14		GAB1														
PIK3C2B		HIF1A														
PIK3CD		ITGA4														
PIK3R5		JAK1														
RAF1		MAP3K5														
RASGRF2		MAPK1														
RHOQ		MAPK9														
RHOT2		NNAPEPLD														
RNND2		PAK7														
SMAD2		PIK3R1														
SMAD4		PLLCB1														
SMO		PRKACB														
SUV39H1		PSEN1														
TP53		RAPGEF1														
VEGFA		RB1														
		RHOC														
		RND3														
		SMAD4														
		SMAD5														
		SMAD6														
		SMAD7														
		SOS1														
		STAT3														
		TCF4														
		TGFBR2														
		VEGFA														
		XIAP														

B **Predicted mRNA targets for miRNA higher in A818.4 than A818.4-CD44v6kd TEX (EMT-related*)**

let7a	miR-101-3p	miR-103a-3p	miR-130a-3p	miR-22-3p	miR-494-3p	miR-92a-3p
AKT2	AKT3	CCNE1	CDKN1A	AKT3	APC	CDC42
BCL2L1	BCL9	FGF18	E2F2	BCL9	FGF16	CDKN1A
CCND1	ETS1	FGF2	FZD3	CDKN1A	FGF7	E2F3
CDKN1A	FGFR3	FGF7	FZD6	MAP2K4	FGF9	FRS2
DVL3	FRS2	FGFRL1	MAML1	MAPK10	FGFR2	FZD10
E2F2	FZD4	FIGF	MAPK1	NRAS	FZD2	HMGA2
E2F5	FZD6	FZD4	MAPK10	PIK3CD	HMOX1	IRS2
E2F6	GAB1	IRS2	MET	SMAD4	IRS1	MAP2K4
FGF5	GSK3B	JAK1	PIK3C2A	SNAI1	MDM2	NOTCH1
FRS2	JAK2	MAML1	RALBP1	TGFBR1	PIK3R3	PIK3R3
FZD3	MAPK1	NOTCH2	SMAD4	TP53	PTPN11	SMURF1
FZD4	MET	PIK3R1	SOS2	WNT1	TCF7L2	SNAI1
HBEGF	PIK3C2A	SOS1	STAT3		WNT5A	TCF4
HMGA2	PIK3C2B	TGFBR2	SUV39H1			TGFB2
HMOX1	PTGS2	WNT16	TCF4			TWIST1
HRAS	RAC1	WNT3A	TGFA			WNT5A
IRS2	RALBP1	WNT4	TGFB2			ZEB2
KRAS	SMAD2		TGFBR1			
MAPK9	TCF4		TGFBR2			
NAPEPLD	TGFA		WNT1			
NRAS	TGFBR1		WNT10A			
PLD3	ZEB1		WNT2B			
PTGS2	ZEB2		ZEB1			
PYGO2			ZEB2			
Ras						
RB1						
SMAD2						
SMAD2						
TGFBR1						
TGFBR1						
TP53						
WNT1						

* predicted target for additional EMT-related miRNA
(miR-125a-5p, miR-151a-3p, miR-192-5p, miR-194-5p, miR196a-5p, miR26b-5p, miR-31-5p, miR331-3p, miR-374a-5p, miR-7-5p, miR-99b-5p) are shown in Fig.S3A)

C

D

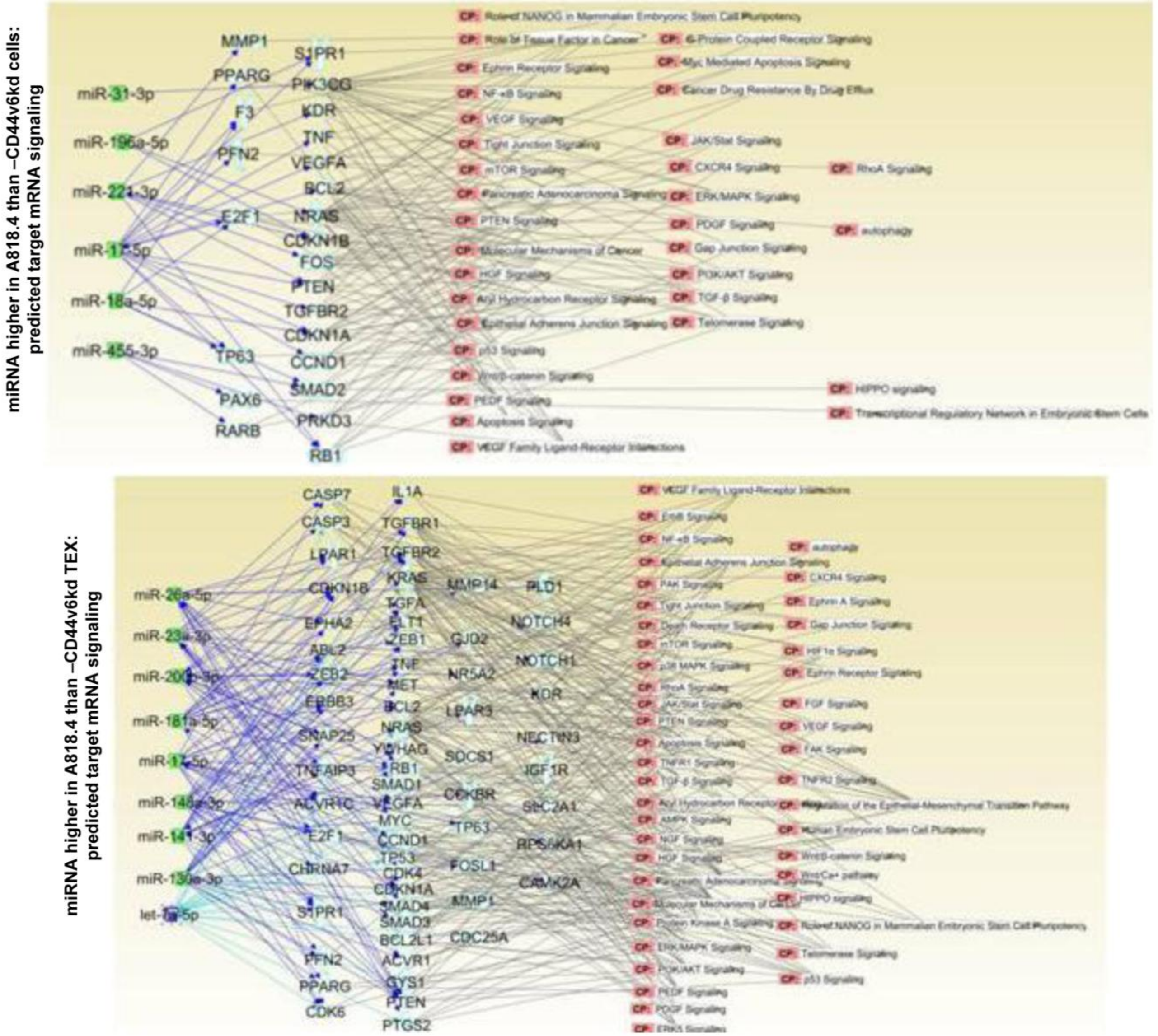

Figure S3 Predicted targets of miRNA higher in A818.4-wt than -CD44v6kd cells or TEX. mRNA predictions are based on miRNA and target scan databases. (A) Predicted mRNA for miRNA higher in A818.4-wt than -CD44v6kd cells were selected according to engagement in cancer-related signaling (KEGG program); (B) predicted mRNA for miRNA higher in A818.4-wt than -CD44v6kd TEX were selected according to engagement in EMT (KEGG program). (C,D) IPA-based KEGG analysis was used to search for predicted targets engaged in cancer-related signaling pathways of 6 miRNA higher in wt than CD44v6kd cells and 9 miRNA higher in wt than CD44v6kd TEX. (full names of synonyms: Table S6).

A high number of predicted mRNA targets of miRNA distinctly processed in cells depending on CD44v6 are engaged in cancer-related signaling and a high number of predicted mRNA targets of miRNA distinctly recovered in TEX depending on CD44v6 are engaged in EMT. These miRNA target molecules are engaged in a wide range of signaling pathways, connectivity in TEX exceeding that in cells.

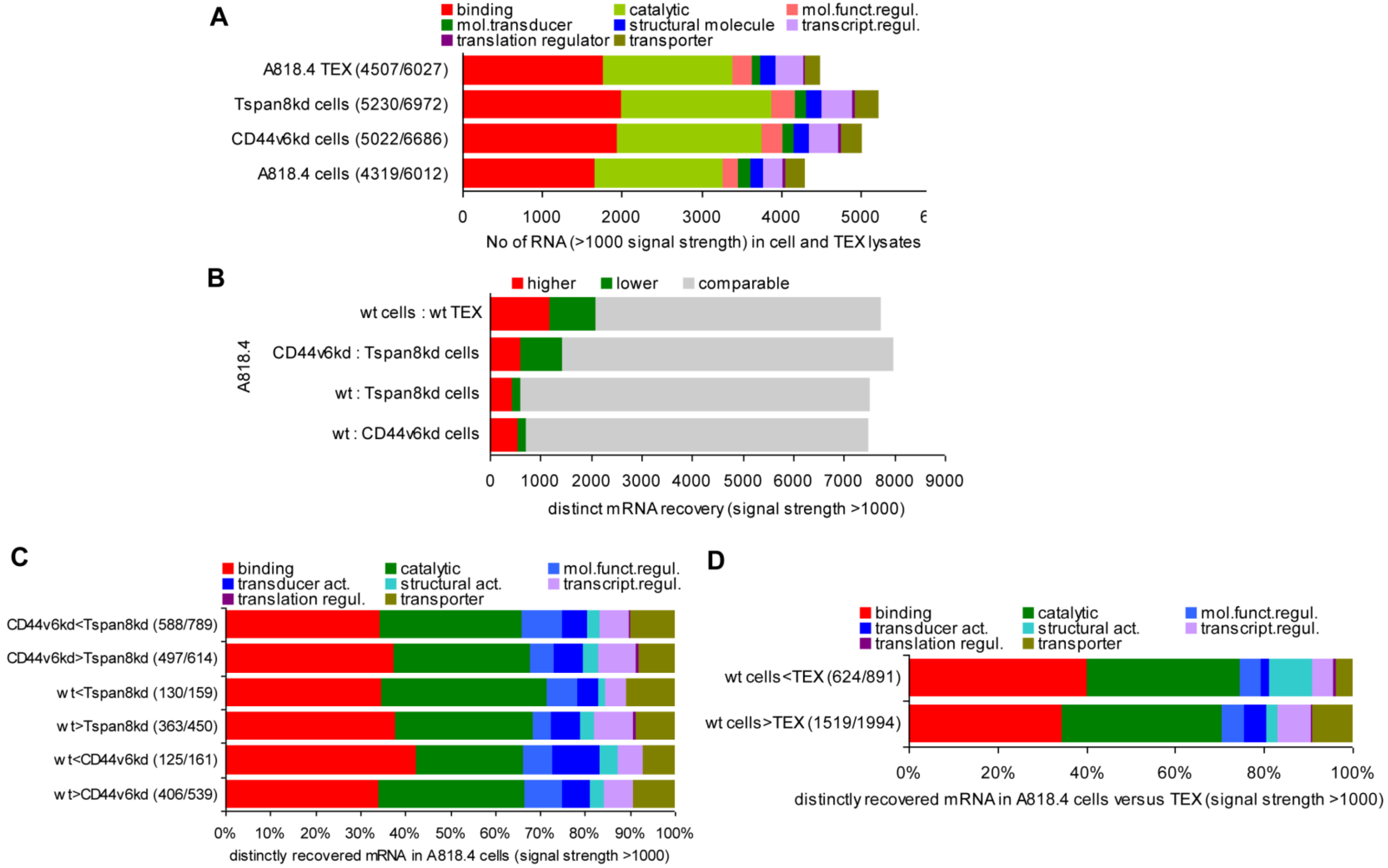

Figure S4 Impact of CD44v6 and Tspan8 on the mRNA profile in cells and TEX. mRNA in wt, CD44v6kd and Tspan8kd cells and TEX was evaluated by DS. (A) Overview on molecular functions (Panther pathway analysis) of RNA (signal strength >1000) revealed an abundance of binding and catalytic RNA in wt and kd cells and TEX; (B) mRNA was sorted according to ≥2-fold differences in signal strength between wt and kd cells and wt cells versus TEX; Comparison of molecular functions (Panther pathway analysis) of distinctly recovered mRNA in (C) wt versus kd cells and (D) wt cells versus TEX.

Molecular functions of wt and kd cell and TEX mRNA did not significantly differ. The vast majority of mRNA is recovered at a comparable level in wt and kd cells. A slight increase in distinctly recovered mRNA is seen comparing cells with TEX. Analyzing molecular function of distinctly recovered mRNA revealed only minor differences between wt versus kd cells and wt cells versus TEX.

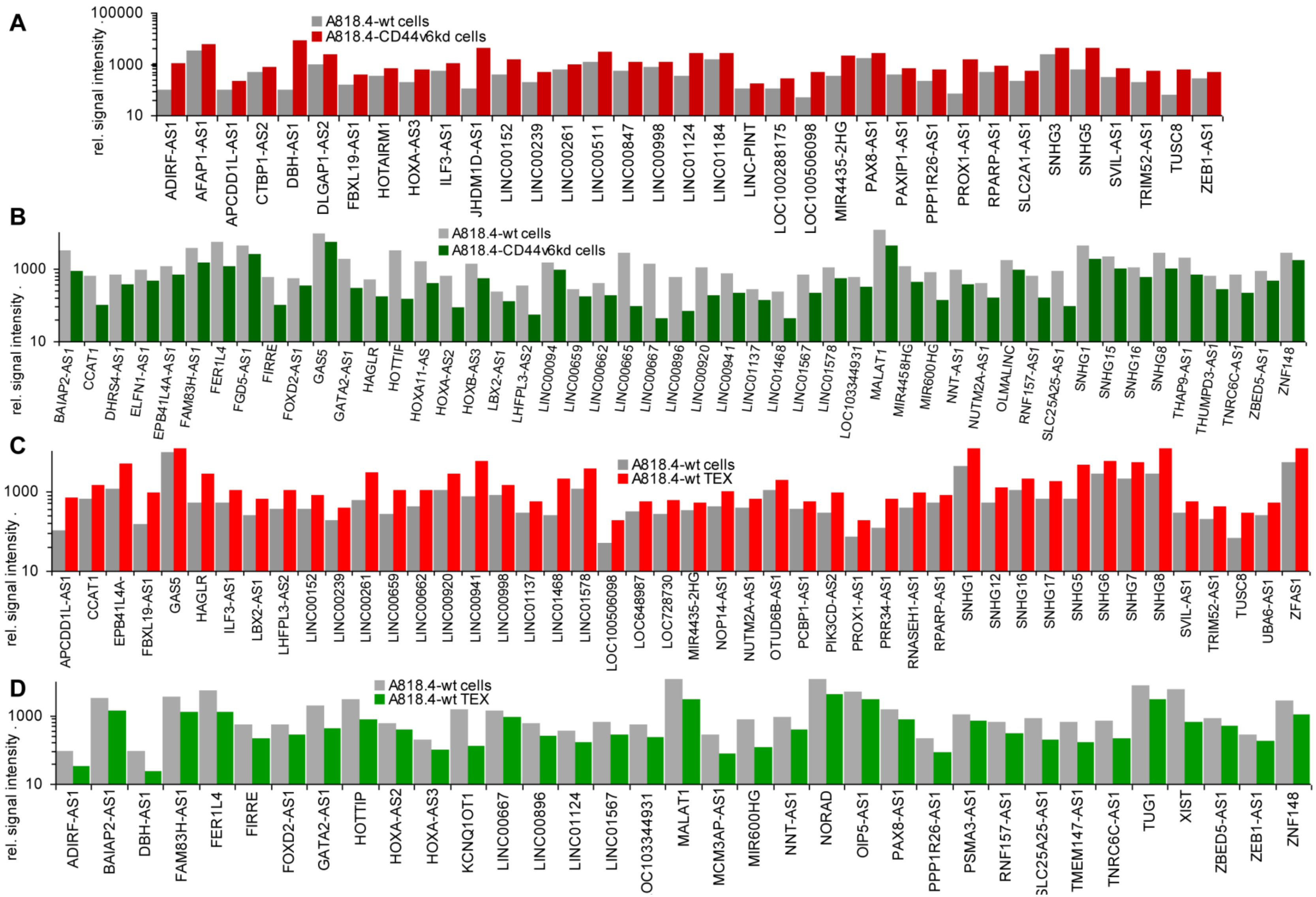

Figure S5 Distinct recovery of lncRNA. Recovery of lncRNA was evaluated by DS in A818.4 wt and CD44v6kd cells and in wt TEX. The relative signal strength of lncRNA being ≥1.5-fold (A) reduced or (B) increased in wt cells versus CD44v6kd cells and (C) reduced or (D) increased in wt cells versus TEX. (Full name of synonyms in Table S7)
A considerable number of lncRNA is distinctly recovered in wt cells compared to CD44v6kd cells or wt TEX, although signal strength was mostly low.
